# Identification of Three New Rugose Small Colony Variants from a *Pseudomonas aeruginosa* Biofilm

**DOI:** 10.3390/microorganisms13112550

**Published:** 2025-11-07

**Authors:** Benjamin K. Smartnick, Eric A. Carlson, Chase N. Morse, Taylor A. Dodson, Nathan C. Wamer, Avery M. Horne, Erin G. Prestwich

**Affiliations:** Department of Medicinal and Biological Chemistry, University of Toledo, Toledo, OH 43606, USA

**Keywords:** *Pseudomonas aeruginosa*, biofilms, rugose small colony variants, second messenger signaling, RNA sequencing, DNA sequencing, multi-disciplinary, pathogen

## Abstract

*Pseudomonas aeruginosa* is a Gram-negative, pathogenic, bacterium that produces biofilms comprising phenotypically distinct cell subpopulations. When separating and characterizing a single *P. aeruginosa* PA14 biofilm, three novel rugose small colony variants (RSCVs) (denoted RSCV_1, RSCV_2, and RSCV_3) were discovered. Characteristics of these stationary phase RSCVs differed between stationary phase wild-type (WT) PA14, between the PA14 biofilm subpopulations, and between the RSCVs themselves. The observed phenotypic changes in the RSCVs included differences in cellular morphology, exopolysaccharide production, biosynthesis of virulence factors, biofilm formation, and antibiotic tolerance. Stationary phase cell surface-associated molecules on the RSCVs were differently ionized as compared to WT PA14 using matrix-assisted laser desorption ionization (MALDI) mass spectrometry. Many RNA transcripts were differentially expressed between the RSCVs and WT PA14 as well as between RSCV_1 and RSCV_3. DNA sequencing revealed single-nucleotide deletions and single-nucleotide polymorphisms (SNPs) among the RSCVs and between the RSCVs and WT PA14. The levels of the intracellular signaling molecule bis-(3′,5′)-cyclic-dimeric-guanosine monophosphate (cyclic-di-GMP) were higher in the RSCVs compared to WT PA14 and significantly lower in RSCV_3 as compared to both RSCV_1 and RSCV_2. The detected differences in the RSCVs have significant implications for biofilm production, antibiotic tolerance, and virulence.

## 1. Introduction

*Pseudomonas aeruginosa* is a pathogenic, Gram-negative bacterium capable of forming biofilms in both environmental and medical settings, including within the lungs of individuals with cystic fibrosis (CF) [1]. Bacterial biofilms occur when cells attach to each other or a surface, aggregate, and become encased in a structural extracellular matrix [2]. Secreted polysaccharides, proteins, and extracellular DNA (eDNA) comprise the extracellular matrix [3,4]. The positively charged exopolysaccharide, Pel, is able to interact with the negatively charged eDNA in *P. aeruginosa* PA14 biofilms, adding to the structural integrity of the biofilm [5]. *P. aeruginosa* biofilms are more tolerant to a variety of antibiotics due to the physical barrier of the extracellular matrix and an increased expression of cellular antibiotic resistance genes, including multidrug efflux pumps [6,7,8,9]. *P. aeruginosa* biofilms are regulated by two-component regulatory systems, quorum sensing signaling molecules, and the intracellular signaling molecule bis-(3′,5′)-cyclic-dimeric-guanosine monophosphate (cyclic-di-GMP) [10,11,12,13]. Released metabolites of *P. aeruginosa*, such as phenazines and siderophores, serve multiple significant functions. Phenazines bind and transport metal ions, act as virulence factors, and regulate metabolic activity in oxygen-deficient environments, and their production is heavily influenced by the intracellular concentrations of cyclic-di-GMP [14,15,16,17,18]. Overall, *P. aeruginosa* biofilms are influenced by a number of different regulatory systems and signaling molecules.

Rugose small colony variants (RSCVs) differentiate themselves from the WT strain by their mutations; their small, wrinkled and rough colony morphologies; elevated levels of cyclic-di-GMP; and the fact that they are hyper-biofilm producers [19]. The hyper-biofilm-producing nature of the RSCVs have caused them to be implicated in unique medical challenges compared to WT *P. aeruginosa* cells, with RSCVs being detected as highly abundant in chronic infections [20,21]. Recent studies have shown that RSCVs contain unique genetic mutations in the wrinkly spreader phenotype (Wsp) that result in the over-production of the intracellular signaling molecule cyclic-di-GMP [20,21,22]. The Wsp signal transduction system is in a genomic island that begins with the membrane-bound WspA receptor and ends with the phosphorylation of the diguanylate cyclase WspR [23]. The loss of the methylesterase, WspF, locks WspR in an active state and correlates with the RSCV phenotype and hyper-biofilm production [24,25]. As well as biofilm formation, cyclic-di-GMP has also been shown to play significant roles in the regulation of bacterial systems [26]. Cyclic-di-GMP regulates bacterial motility and exopolysaccharide production [10]. For example, cyclic-di-GMP interacts with the flagellar motility transcriptional regulator FleQ and the polysaccharide biosynthesis protein PelD [26,27]. RSCVs display many phenotypes associated with an increased production of cyclic-di-GMP such as lowered motility, increased surface attachment, and hyper-biofilm formation [28].

Previously, we discovered three phenotypically distinct cell subpopulations in a *P. aeruginosa* PA14 biofilm [29]. While plating each of these biofilm cell subpopulations, small colony variants were observed that were morphologically distinct from the biofilm subpopulation colonies [29]. When the subpopulations were re-grown after plating, the morphologies were similar to wild-type PA14, suggesting these subpopulations were transient [29]. However, when re-growing the small colony variants, the morphologies remained constant, suggesting that they were genetically distinct. This resulted in the identification of three distinct RSCVs that isolated with one of the three biofilm subpopulations: RSCV_1 with the biofilm cells (BF), RSCV_2 with extracellular-matrix cells (ECM), and RSCV_3 with the supernatant cells (SP). These RSCVs, in stationary phase, displayed classical RSCV phenotypes but varied from each other and to stationary phase WT PA14 [21,22,30]. This study provides further information into the *P. aeruginosa* biofilm and identifies three phenotypically, transcriptionally, and genetically distinct novel RSCVs isolated from the same *P. aeruginosa* PA14 biofilm.

## 2. Materials and Methods

### 2.1. Materials

*Pseudomonas aeruginosa* UCBPP-PA14 was donated from the laboratory of Peter C. Dedon at MIT. Cells were propagated in Luria–Bertani (LB) broth, Miller (Fisher BioReagents, Pittsburgh, PA, USA), or LB agar, Miller (Fisher BioReagents, Pittsburgh, PA, USA), dissolved in MilliQ-H_2_O (Millipore, Burlington, MA, USA).

### 2.2. Cell Growth and Culture Conditions

The planktonic cell cultures and biofilms were grown, and the biofilm cell subpopulations were isolated according to the published protocol [29]. *P. aeruginosa* planktonic cultures were inoculated by picking a single colony from an LB agar plate and incubating at 37 °C for 16 to 18 h with constant shaking at 280 rpm. To make the biofilm, stationary phase WT PA14 cells were normalized to an OD_600_ of 0.3 ± 0.01 and were inoculated 1:500 into 50 mL of LB in a 150 mm glass petri dish. The inoculated biofilms were then incubated at 37 °C for 48 h without shaking. To separate the biofilm subpopulations, the biofilms were centrifuged at 11,000× *g* (Beckman J2-21M/E Centrifuge, JA-14 rotor) for 45 min, which resulted in a BF/ECM cell pellet. The supernatant was then transferred to a new centrifuge tube and centrifuged at 28,000× *g* (Beckman J2-21M/E Centrifuge, JA-14 rotor) for 3 h. The cell pellets containing both the BF and ECM cells were then separated by light shaking with the ECM cells then being transferred to an enzymatic digest. The ECM enzymatic digest consisted of 13 mL of 0.9% saline, 1 mM MgCl_2_ (99% ACS reagent, Acros Organics, Morris Plains, NJ, USA), 25 units of alginate lyase (Part number A1603, powder, ≥10,000 units/g), and 750 units of DNase I (Sigma-Aldrich, St. Louis, MO, USA). The ECM enzymatic digest was incubated with constant shaking at 280 rpm at 37 °C for 5 h and subsequently centrifuged at 12,000× *g* (Beckman J2-21M/E Centrifuge, JA-20 rotor) for 30 min. All of the isolated biofilm subpopulation cell pellets were washed twice with 0.9% saline prior to diluting and plating onto LB agar. The plated biofilm subpopulations were incubated for 16 to 18 h at 37 °C. One RSCV from each of the three biofilm subpopulations was picked and grown in LB broth at 37 °C for 16 to 18 h with orbital shaking at 280 rpm. Frozen glycerol stocks of each RSCV were then made for future experiments.

Single colonies of either WT PA14 or the RSCVs were picked into 3–4 mL of media and grown at 37 °C with orbital shaking. For the growth curve and Congo red experiments involving M9 minimal media, the composition of the minimal media was as follows: 0.4% (*w*/*v*) glucose (Fisher BioReagents, Pittsburgh, PA, USA), 2 mM MgSO_4_ (Fisher BioReagents, NJ, USA), 0.1 mM CaCl_2_ (Fisher BioReagents, NJ, USA), 24 mM Na_2_HPO_4_ (Research Products International, Prospect, IL, USA), 11 mM KH_2_PO_4_ (Fisher BioReagents, NJ, USA), 4.1 mM NaCl (Fisher BioReagents, NJ, USA), and 9.35 mM NH_4_Cl (Sigma-Aldrich, MO, USA). All stationary phase cells were grown for 16 to 18 h, and all mid-log phase cells were grown for 7 h. All turbidity measurements were performed with a Multiskan Spectrum microplate and cuvette reader with SkanIt RE for MSS 2.4.4 software (Thermo Electron Corporation, Waltham, MA, USA).

### 2.3. Zeta Potential and DLS Particle Size Analysis

Zeta potential and dynamic light scattering (DLS) measurements were taken using an Anton Paar Litesizer^TM^ 500 with Kalliope^TM^ software (version 3.4.5) (Anton Paar, Graz, Austria). For zeta potential measurements, cells were pelleted at 16,000× *g* (Hermle Z233 M-2 Centrifuge) and solubilized in 0.02 µm filtered saline (0.9% *w*/*v* NaCl), normalized to an OD_600_ 0.3 ± 0.01, and placed in gold electrode omega cuvettes (Anton 40 Paar, Graz, Austria). Measurements were performed using standard aqueous method settings.

Particle size was measured using DLS by taking normalized cells (OD_600_ 0.3 ± 0.01) and diluted 1:50 with 0.02 µm filtered saline in polystyrene cuvettes. Particle size analysis was carried out at room temperature with bacteria (refractive index 1.388) and the aqueous 154 mM NaCl (refractive index 1.3323).

### 2.4. Swarming and Twitching Assays

Swarming assays were performed as previously described with a few modifications [31]. Modified M9 minimal media containing 0.2% (*w*/*v*) glucose (Fisher BioReagents, Fair Lawn, NJ, USA); 1 mM MgSO_4_ (Fisher BioReagents, NJ, USA); 1 mM CaCl_2_ (Fisher BioReagents, NJ, USA); 0.05% (*w*/*v*) casamino acids (Bio Basic, Amherst, NY, USA); and 1X swarming salts (19.97 mM NH_4_Cl (Sigma-Aldrich, MO, USA), 11.99 mM Na_2_HPO_4_ (Research Products International, Prospect, IL, USA), 21.99 mM KH_2_PO_4_ (Fisher BioReagents, NJ, USA), and 8.59 mM NaCl (Fisher BioReagents, NJ, USA)) in 100 mm plates with 0.25% (*w*/*v*) agar were used in the swarming experiments [32]. The plates, immediately after being poured, were treated with UV for 20 min and afterwards air dried for 40 min. The plates were then inoculated with 5.0 µL of a normalized OD_600_ 0.3 ± 0.01 culture and incubated for 24 h at 37 °C. Plate images used for quantification were obtained using a 12 Mega Pixel (MP) iPhone 12 camera. ImageJ software (version 1.8.0) was used to quantify the total swarming area of each plate [33].

Twitching motility assays were conducted similarly to previously published methods [34,35]. A single colony was picked with a pipette tip and stabbed into a 1% (*w*/*v*) agar LB plate. Multiple individual colonies of each variant were stabbed for each plate. Twitching plates containing 15 µg/mL of gentamycin sulfate (part number J62834.09, Thermo Scientific, Waltham, MA, USA) were stabbed with a transposon mutant of *pilT* (PA14_05180) [36]. The twitching plates were then incubated at 37 °C for 24 h. After incubation, the agar was carefully removed from the twitching plate, and the bottom was stained with a 0.1% (*w*/*v*) crystal violet solution (Acros Organics, NJ, USA) for approximately 10 min. Pictures were taken using the Azure C500 (version 1.6.12.0224) equipped with an 8.3 MP camera, and the twitching areas were quantified using ImageJ software [33].

### 2.5. Crystal Violet Biofilm Assays

Biofilms were stained with crystal violet based on a published protocol [37]. From a cell culture normalized to an OD_600_ of 0.3 ± 0.01, 1 µL of that normalized culture was inoculated into 99 µL of LB broth in a 96-well plate, such that the initial average OD_600_ of 0.0014 started the assay. The 96-well plate was incubated at 37 °C for either 4 h or 24 h, based on the experiment being performed, without shaking. The 96-well plates were then submerged twice into water baths to wash out the wells. The biofilms were stained by adding 125 µL of a 0.1% crystal violet solution to each well and incubated at room temperature for approximately 10 min. Crystal violet solution was removed by dipping the plate twice into deionized water. Once the 96-well plate was air dried, 125 µL of 30% acetic acid (Sigma-Aldrich, MO, USA) was added to each well. The absorbance of each well containing the acetic acid solution was quantified at 550 nm as previously described [37]. Blank wells, which were LB broth that was not inoculated with bacteria and stained with crystal violet, were subtracted to adjust for any excess crystal violet.

Both the total biofilm antibiotic accumulation and the biofilm dissociation assays were based on a previous study [29]. Biofilm accumulation crystal violet assays were incubated for 24 h, while biofilm dissociation assays were incubated for a total of 48 h. Accumulation assays were performed identically as described above but with the addition of either colistin sulphate salt (part number 455390010, Acros Organics, NJ, USA) or tobramycin (part number T4014, Sigma-Aldrich, MO, USA). Five concentrations of both colistin (0.2 µg/mL, 0.4 µg/mL, 0.6 µg/mL, 0.8 µg/mL, and 1.0 µg/mL) and tobramycin (0.5 µM, 1.0 µM, 1.5 µM, 2.0 µM, and 3.0 µM) were tested for each biological replicate. For dissociation assays, after 24 h of growth with no antibiotic, the broth was replaced with new LB broth containing various concentrations of colistin (30 µg/mL, 50 µg/mL, 70 µg/mL, 100 µg/mL, and 150 µg/mL) and tobramycin (20 µM, 40 µM, 60 µM, 100 µM, and 150 µM). The dissociation assay 96-well plate was then incubated for an additional 24 h at 37 °C. All of the antibiotic crystal violet assays were normalized to the wells with no antibiotic.

### 2.6. Congo Red Assay

LB broth containing 40 µg/mL of Congo red dye (ACS reagent, Chem-Impex, 22807, Wood Dale, IL, USA) was used to indirectly quantify exopolysaccharide production. Cells were normalized to an OD_600_ of 0.3 ± 0.01, inoculated 1:1000 into the broth, and incubated for 18 h at 37 °C with constant shaking. Cells and precipitated Congo red dye were pelleted by centrifugation at 16,000× *g* (Hermle Z233 M-2 Centrifuge) for 2 min at 4 °C. The absorbance of the resulting supernatant was measured at 490 nm to quantify the remaining Congo red in solution as previously described [38].

### 2.7. Phenazine and Siderophore Isolation and Quantification

Cells were pelleted from stationary phase cultures of RSCV_1, RSCV_2, RSCV_3, and WT PA14 by centrifugation at 16,000× *g* (Fisher Scientific accuSpin Micro 17R Centrifuge) for 10 min at 4 °C. The supernatant was filtered through 0.2 µm polytetrafluoroethylene (PTFE) syringe filters, and 500 µL of chloroform was added to each filtered supernatant to extract the phenazines and siderophores. The samples were vortexed and centrifuged, and the organic layer was saved. Another 500 µL of chloroform was added to the aqueous layer, vortexed, centrifuged, and retained the organic layer to extract residual phenazines and siderophores. To remove residual aqueous solution, the chloroform solution was then incubated at −20 °C for 1 h, which allows the aqueous solution to freeze. The chloroform was then filtered through a 0.2 µm nylon syringe filter. The chloroform samples containing the isolated phenazines were dried down and solubilized in 100 µL of 40:60 acetonitrile/water (*v*/*v*) buffer for HPLC-UV analysis and filtered through a 0.2 µm nylon filter [39].

Phenazines were quantified through HPLC-UV using a Kinetex 1.7 µm EVO C18 100Å column (100 × 2.1 mm) (Phenomenex, Torrance, CA, USA) [39]. HPLC separation was performed on a Nexera XR HPLC (Shimadzu Scientific Instruments, Inc., Kyoto, Japan) using reverse phase chromatography with neutral pH MilliQ-H_2_O as mobile phase A and acetonitrile (HPLC-UV grade, Sigma-Aldrich, MO, USA) as mobile phase B, with a column oven temperature of 30 °C and a flow rate of 0.2 mL/min. HPLC gradients from 0 to 10 min of 10–20% B and 10 to 25 min of 20–40% B were utilized to separate pyocyanin, phenazine-1-carboxylic acid, phenazine-1-carboxamide, and hydroxyphenazine. One µL injections of the samples were quantified by scanning at 366 nm [14]. The column was washed and re-equilibrated between each sample. Commercially bought standards of pyocyanin, hydroxyphenazine (Cayman Chemicals, Ann Arbor, MI, USA), phenazine-1-carboxylic acid, and phenazine-1-carboxamide (ChemScene, Monmouth Junction, NJ, USA), were used for retention time testing and to generate HPLC-UV calibration curves. All standards were solubilized in MilliQ-H_2_O prior to HPLC separation.

To quantify the pyoverdine and pyochelin siderophores, cells from cultures grown to stationary phase were pelleted by centrifugation at 16,000× *g* (Fisher Scientific accuSpin Micro 17R Centrifuge) for 10 min at 4 °C. The supernatant was then filtered through 0.2 µm PTFE syringe filters. Filtered samples were placed into black 96-well plates, and the relative fluorescence was measured with a CLARIO star (MBG Labtech, Cary, NC, USA) with CLARIO star data analysis (version 3.01R). Pyoverdine and pyochelin were monitored through excitation–emission wavelengths of 400–447 nm and 355–430 nm, respectively [40].

### 2.8. MALDI Procedure

All MALDI experiments were carried out using a UltrafleXtreme MALDI-TOF/TOF with flexControl software (version 3.4) (Bruker, Billerica, MA, USA). Collection of unique ion lists was carried out in reflectron positive and negative modes with a frequency-tripled Nd: YAG laser (355 nm).

To determine ions unique to each of the RSCVs and WT PA14, cultures were grown for 16 to 18 h at 37 °C. A 600 µL aliquot of cells was removed and centrifuged at 16,000× *g* (Fisher Scientific accuSpin Micro 17R Centrifuge) for 10 min at 4 °C. Cell pellets were washed and resuspended in 30 µL of 0.9% saline, and 1 µL was spotted onto an indium-titanium oxide (ITO) glass slide (Bruker, Billerica, MA, USA). Six biological replicates were plated for each RSCV and WT PA14 and gold sputtered for 3 s (1 to 2 nm). All experiments were calibrated with elemental sulfur cluster S_11_ (*m*/*z* 351.693) [41,42]. Scans were performed at identical laser intensities in both positive and negative ionization mode. A total of 10,000 laser pulses were performed per sample with a mass range between 150 and 1000 *m*/*z.* All *m*/*z* peaks that were above a signal-to-noise ratio threshold of three were collected for unique ion comparisons. Ions that were in 50% or more of the replicates were compared between the RSCVs and WT PA14 to determine unique ions. Collision-induced dissociation (CID) with argon was performed to putatively identify specific unique ions [42].

### 2.9. DNA Isolation

DNA isolation methods were modified from previously published methods [43]. Cultures of RSCV_1, RSCV_2, RSCV_3, and WT PA14 were grown to stationary phase. Cell culture was removed and centrifuged at 16,000× *g* (Fisher Scientific accuSpin Micro 17R Centrifuge) for 10 min at 4 °C to pellet the cells. The supernatant was removed, and cells were washed twice with saline (0.9% *w*/*v* NaCl). The cells were suspended in 1 mL of DNA isolation buffer consisting of 100 mM Tris (Fisher BioReagents, NJ, USA), 1.4 M sodium chloride (Fisher BioReagents, NJ, USA), 20 mM ethylenediaminetetraacetic acid (EDTA) (Fisher BioReagents, NJ, USA), and 2% *w*/*v* hexadecyltrimethylammonium bromide (Acros Organics, NJ, USA). To lyse the cells, 50 mg/mL polyvinylpolypyrrolidone (PVPP) (Sigma-Aldrich, MO, USA) and 0.1 mL β-mercaptoethanol (Millipore Sigma, St. Louis, MO, USA) were added. The solution was vortexed briefly and then incubated at 55 °C for 30 min. An equal volume of 24:1 chloroform (Fisher Chemical, Hampton, New Hampshire): isoamyl alcohol (Bio Basic, Markham, Ontario, Canada) was added, vortexed, and centrifuged at 17,000× *g* for 25 min. The aqueous layer was removed and 24:1 chloroform: isoamyl alcohol extraction step was repeated. The aqueous layer was removed and placed in a new tube, and nucleic acid was precipitated by adding at least 2 volumes of 100% ethanol, incubated 60 minutes at −20 °C, and then centrifuged to pellet the nucleic acids. The nucleic acid was resolubilized in nuclease-free MilliQ-H_2_O. An amount of 3.5 Kunits of RNase (Millipore Sigma, MO, USA) were added to each sample and incubated at 37 °C for 1 h. Proteins were digested by adding 5.0 µL of 25 mg/mL proteinase K (Goldbio, P-480-1, St. Louis, MO, USA) and incubated at 50 °C for 1 h. The aqueous layer was extracted by an additional phenol/chloroform/isoamyl alcohol (25:24:1 *v*/*v*/*v*, pH 8), two chloroform extractions, and one diethyl ether liquid–liquid extraction with the aqueous phase retained each time [44]. The final aqueous solution was transferred to a new tube and the DNA was precipitated by adding 2 volumes of 100% ethanol, incubated at −20 °C for at least 1 h, and centrifuged. DNA was solubilized in nuclease-free MilliQ-H_2_O and quantified through UV-Vis at 260 nm [45].

### 2.10. Cyclic-Di-GMP Isolation

Isolation of the intracellular signaling molecule cyclic-di-GMP was adapted and modified from previous studies [46,47]. Single colonies of RSCV_1, RSCV_2, RSCV_3, and WT PA14 were inoculated into 4 mL of LB and incubated at 37 °C for 16 to 18 h with constant shaking. A total of 1 mL of cell culture was centrifuged at 16,000× *g* (Hermle Z233 M-2 Centrifuge) at 4 °C for 2 min to pellet the cells. Cell pellets were then washed with 0.9% (*w*/*v*) saline. An extraction buffer of 2:2:1 of acetonitrile/methanol/0.1 N formic acid in MilliQ-H_2_O was prepared and stored at −20 °C. An amount of 500 µL of the extraction buffer was added to each of the cell pellets, then briefly vortexed. A set amount of 8-bromoadenosine 3′,5′-cyclic monophosphate (Br-cAMP) (Sigma-Aldrich, MO, USA) was used as an internal standard and was added to each sample prior to the cyclic-di-GMP isolation. The cells with the extraction buffer were then incubated at −20 °C for 30 min. Following the incubation, 10 µL of 15% ammonium bicarbonate was added to each of the samples to neutralize the formic acid in the extraction buffer. The samples were again centrifuged at 16,000× *g* (Hermle Z233 M-2 Centrifuge) for 2 min at 4 °C to pellet the left-over cellular debris. The supernatant was then incubated at −80 °C for 20 min and then immediately extracted twice with equal volumes of 24:1 chloroform/isoamyl alcohol; twice with chloroform; and lastly twice with diethyl ether with the aqueous layer retained for each extraction. The final extracted aqueous layer was then frozen at −80 °C for 30 min before being dried under vacuum and resuspended in 20 µL sterile nuclease-free MilliQ-H_2_O. The resuspended samples were then stored at −80 °C prior to LC-MS/MS analysis. To normalize the cyclic-di-GMP to the number of CFUs in the isolation, each culture was diluted and plated on 3–6 LB agar plates. The LB agar plates were then incubated at 37 °C for 24 h. The number of colonies per plate were counted and used to calculate the average CFUs in each culture.

### 2.11. LC-MS/MS Cyclic-Di-GMP Analysis

The cyclic-di-GMP cell isolates were separated with a Shimadzu Nexera XR HPLC system equipped with a photodiode array detector (PDA). Cyclic-di-GMP was quantified using a Shimadzu 8050 LC-MS triple quadrupole mass spectrometer with LabSolutions software (version 5.85). A Thermo Hypersil gold C18 HPLC column (250 × 2.1 mm, 5 µm particle size) was used for the HPLC separation [47]. A solvent system of 0.1% formic acid (Sigma-Aldrich, MO, USA) in MilliQ-H_2_O (mobile phase A) and a 0.1% formic acid in acetonitrile (>99.9% purity Optima grade, Fisher Chemicals, Zurich, Switzerland) (mobile phase B) was used with a column oven temperature of 30 °C and a flow rate of 0.3 mL/min [47]. A multi-step binary gradient was utilized: an isocratic 0% B for 0–10 min, a 0–5% B gradient between 10 and 20 min, and a 5–10% B gradient between 20 and 25 min. The column was washed with 98% acetonitrile for 15 min and re-equilibrated with 100% mobile phase A for 30 min. Samples were quantified through mass spectrometry in positive ionization mode utilizing multiple reaction monitoring (MRM). The mass spectrometer parameters were as follows: a 3 L/min nebulizing gas flow, 3 L/min heating gas flow, interface temperature of 350 °C, a DL temperature of 200 °C, a drying gas flow of 17 L/min, a scan rate of 40 scans per second, and a heat block temperature of 500 °C. The 691 > 152 *m*/*z* and 691 > 248 *m*/*z* fragments were used as the quantifier and qualifier ions, respectively. Likewise, the 708 > 152 *m*/*z* and 708 > 596 *m*/*z* fragments were used as the quantifier and qualifier fragment ions, respectively, for 5′-phosphoguanylyl-(3′,5′)-guanosine (pGpG). Commercially bought standards of cyclic-di-GMP (Cayman Chemicals, MI, USA) and pGpG (Cayman Chemicals, MI, USA) were used for MRM optimization and calibration curves.

### 2.12. DNA Sequencing Analysis

All three RSCVs and WT PA14 were sequenced by GeneWiz (South Plainfield, NJ, USA) using SMRT sequencing technology on the PacBio Sequel System with de novo genome assemblies generated through the Canu genome assembler [48]. DNA sequences have been submitted to the NCBI GenBank database under BioProject PRJNA1244416 (accession numbers SAMN47652032, SAMN47652033, and SAMN47652034). Total genome lengths were 6,559,964 bp for stationary WT PA14, 6,552,049 bp for RSCV_1, 6,553,569 bp for RSCV_2, and 6,552,359 bp for RSCV_3. DNA variant detection was performed through GeneWiz (South Plainfield, NJ, USA) through their ‘variantCaller’ (version 2.1.0) algorithm, which generated GFF (general feature format) files containing the detected DNA variants. The Jalview genome browser (version 2.11.5.0) was then used to visualize and analyze the whole-genome sequence alignments [49]. DNA and amino acid alignments were performed through Jalview using the ClustalW computer program (version 2.1.0) for multiple sequence alignments with default settings [50].

### 2.13. RNA Extraction, Sequencing, and Bioinformatics

Cultures of RSCV_1, RSCV_3, and WT PA14 were grown for 16 to 18 h as similarly described before. The RNA isolation followed a similar procedure as the DNA isolation previously described but with minor alterations. No RNase treatment was utilized following cell lysis. Following proteinase K treatment, a phenol/chloroform/isoamyl alcohol (25:24:1) at pH 6.7 was used [45]. Isolated RNA was treated with DNase (Sigma-Aldrich, MO, USA) prior to sequencing. Total nucleic acid was solubilized in nuclease-free MilliQ-H_2_O and quantified via a UV-Vis spectrophotometer. Isolated RNA from RSCV_1, RSCV_3, and WT PA14 were sequenced by Microbial Genome Sequencing Center (MiGS) (currently SeqCenter LLC (Pittsburgh, PA, USA)) using an Illumina NovaSeq X Plus. Quality control, adapter trimming, read mapping (reference genome *P. aeruginosa* UCBPP-PA14, NCBI GenBank ID CP000438), and read quantifications were all performed by MiGS [51]. The RNA sequencing raw and processed files have been submitted to the NCBI Gene Expression Omnibus (GEO) under the accession number GSE293895. Differential gene expression analysis was performed in RStudio (version 2022.12.0.353) using R packages ‘edgeR’ (3.42.4) and ‘limma’ (3.56.2) [52,53]. Normalization was performed using Upper Quartile normalization. A generalized linear model fit was used to generate the table of top differentially expressed genes with significance thresholds set to *p*-value less than 0.05, |logFC| greater than 1, and false-discovery-rate (FDR) less than 0.05 unless otherwise stated. Batch correction was performed to remove batch effects from the RNA-seq raw counts using R package ‘sva’ (3.48.0) [54]. Volcano plots were generated using R packages ‘ggplot2’ (3.4.4) and ‘ggrepel’ (0.9.4) [55,56]. Heat maps were created using R package ‘pheatmap’ (1.0.12) [57]. KEGG pathway analysis was performed using R package ‘clusterProfiler’ (4.8.3) [58]. Fuzzy clustering analysis on the RNA-seq normalized raw counts was conducted using R packages ‘ggfortify’ (0.4.17) and ‘cluster’ (2.1.8) [59,60]. Principal component analysis (PCA) plot of the normalized raw counts with the 95% confidence intervals was conducted using R package ‘car’ (3.1.3) [61].

### 2.14. SEM Imaging

Cell cultures of WT PA14, RSCV_1, RSCV_2, and RSCV_3 were grown overnight and harvested as previously described. Prior to SEM imaging, 2% (*w*/*v*) paraformaldehyde and 2.5% (*w*/*v*) glutaraldehyde in 0.1 M sodium cacodylate buffer was added to fix the cells overnight (Electron Microscopy Sciences, PA, USA). The cells were then washed and chemically dried as previously described [29]. The cell samples were then coated with a total of 5 to 7 nm of gold, and a JEOL JSM-7500 F Cold Cathode Field Emission Microscope (JEOL Ltd. Tokyo, Japan) was used to capture the SEM images.

### 2.15. Light Microscopy Imaging

Cell cultures of the biofilm cell subpopulations, the RSCVs, or WT PA14 were grown overnight as previously noted and normalized to an OD_600_ of 0.3 ± 0.01. Prior to plating on LB agar, the normalized cells were further diluted 1:10,000 for stationary phase WT PA14, 1:10,000 for WT BF, 1:10,000 for WT ECM, 1:5000 for WT SP, and 1:1000 for the RSCVs. LB agar plates were incubated at 37 °C for 16 to 18 h prior to taking light microscopy images. Light microscopy images were taken using an Olympus SZX7 Stereoscopic Light Microscope with WHSZ 10x eyepieces and an Olympus SC100 camera (Olympus Scientific Solutions American Corp., Center Valley, PA, USA) using the OLYMPUS cellSens Standard 1.18 software. The pixel size of all images is 2 µm.

### 2.16. Statistics

Welch’s unpaired two-tailed t-tests or one-way ANOVA with Tukey’s post hoc test for multiple comparisons were applied for most statistical analyses depending on the experiment performed. Two-way ANOVA with Tukey’s post hoc test for multiple comparisons was also used for the statistical analysis on the crystal violet antibiotic accumulation and dissociation assays. When applicable, Grubb’s test was performed to detect and remove outliers. *p*-values less than 0.05 were considered statistically significant, while *p*-values greater than 0.05 were considered non-significant (ns). GraphPad Prism (version 9.4.0) and Microsoft Excel software were used to perform all statistical analyses.

## 3. Results

### 3.1. Isolation and Initial Identification of Three Distinct RSCVs from a WT PA14 Biofilm

Biofilm cell subpopulations from a *P. aeruginosa* PA14 biofilm were isolated and characterized [29]. *P. aeruginosa* biofilm cell subpopulations were separated by size and cell density through a series of centrifugations that result in pellets of BF cells, ECM cells, and SP cells [29]. The swarming motility, growth patterns, and antibiotic tolerance of the subpopulation cells were different between each other and the stationary phase PA14 [29]. When the cells from the biofilm layers were separated and plated, the colony morphologies were different and included small colonies. When the colonies were re-grown and replated, the morphologies of the biofilm subpopulations reverted to wild-type PA14, but the small colonies retained their small colony morphologies [29]. It was initially observed that approximately 10% of the BF and ECM cell colonies and 20% of the SP cell colonies were smaller than the other colonies [29]. The centrifugation properties of the small colony variants implied differences in their cell size, shape, and density. The small colonies were isolated and grown to stationary phase for future experiments (Figure S1). Due to the small colony size and wrinkled colony morphology, the variants were suspected to be RSCVs (Figure S2). The RSCVs that were observed with BF cells are referred to as “RSCV_1”; the RSCVs observed with ECM cells are referred to as “RSCV_2”; the RSCVs observed with SP cells are referred to as “RSCV_3”.

**Figure 1:**
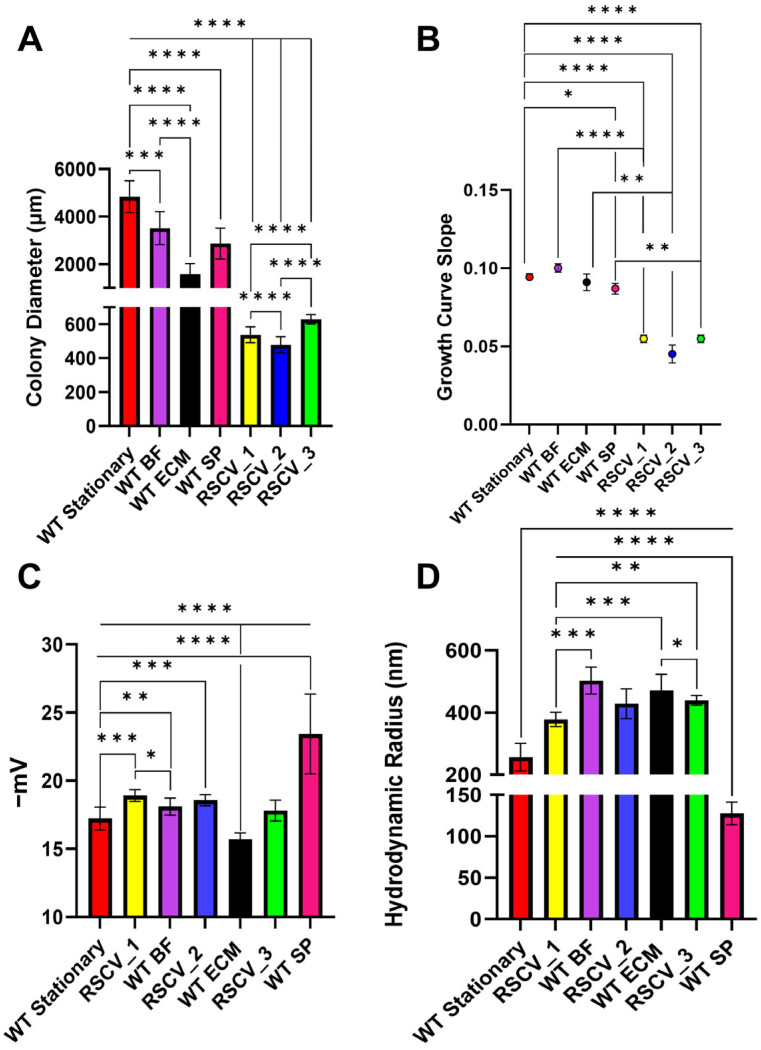
Colony diameters, growth curve slopes, dynamic light scattering (DLS), and zeta potential differences between RSCV_1, RSCV_2, RSCV_3, the WT biofilm subpopulations, and stationary phase WT PA14. The RSCVs had significantly altered cell phenotypes compared to the biofilm subpopulations and stationary phase WT PA14. (**A**) An analysis of the LB agar colony diameters for RSCV_1, RSCV_2, RSCV_3, WT BF, WT ECM, WT SP, and stationary WT PA14 (n = 9–77). (**B**) Growth curve slopes during exponential growth for WT stationary PA14, WT BF, WT ECM, WT SP, RSCV_1, RSCV_2, and RSCV_3 (n = 3–4). (**C**) Zeta potential cell surface charge measurements of RSCV_1 (n = 4), RSCV_2, RSCV_3, WT BF, WT ECM, WT SP, and stationary phase WT PA14 (n = 4–22). (**D**) DLS hydrodynamic radii measurements of stationary phase WT PA14, WT BF, WT ECM, WT SP, RSCV_1, RSCV_2, and RSCV_3 (n = 3–33). Welch’s unpaired two-tailed t-tests were used for all statistical analyses shown. ****, *p* < 0.0001; ***, *p* < 0.001, **, *p* < 0.01, *, *p* < 0.05. See values on Table S1.

Two of the most established hallmarks of RSCVs are a smaller colony size and slower growth rates compared to WT cells [20,22]. When comparing the colony diameters, all three RSCVs had significantly reduced colony diameters when compared to the WT stationary phase colonies (Figure 1A, Table S1) [39]. The WT biofilm subpopulation colonies were significantly larger than the RSCVs; all of the RSCV colonies were three to five times smaller than any of the subpopulation colonies. (Figure 1A, Table S1). The RSCVs had significantly different colony diameters when directly compared to each other with an average of 538 ± 47 µm for RSCV_1, 479 ± 47 µm for RSCV_2, and 629 ± 27 µm for RSCV_3 (Figure 1A, Table S1). All of the RSCV colonies had wrinkled morphologies, irregular colony form, and a crateriform colony elevation (Figure S2) [62,63]. The colony morphologies of the RSCVs were also compared to the morphologies of the WT biofilm subpopulation colonies. While all of the biofilm subpopulations were previously observed to have either wrinkled or hyper-wrinkled colony morphologies [29], significant other differences in colony morphology were noticed between the biofilm subpopulations and the RSCVs. None of the biofilm subpopulations had crateriform colony elevations, which was common to all of the RSCVs (Figures S2 and S3) [62,63]. Colony morphology differences were also observed to exist between the RSCVs. It was noticed that RSCV_1 and RSCV_3 colony margins were undulate, while the RSCV_2 colonies had an erose colony margin (Figure S2) [62,63]. These colony morphologies differ greatly from WT PA14 colonies, which all had circular colony forms and convex colony elevations (Figure S3) [62,63]. Lastly, all three of the RSCVs had significantly reduced growth rates measured during exponential growth phase when compared to stationary phase WT PA14 and the three WT biofilm subpopulations (Figure 1B, Table S1) [39]. The slower growth of the RSCVs compared to stationary WT PA14 was also consistent when the cells were grown in M9 minimal media (Figure S4).

The cellular surface charges (zeta potential) and hydrodynamic radii were compared between the RSCVs, the WT biofilm subpopulation cells, and stationary phase WT PA14 cells (Figure 1C,D, Table S1) [39]. Both RSCV_1 (−18.91 ± 0.43 mV) and RSCV_2 (−18.10 ± 0.41 mV) had a more negative zeta potential when compared to stationary phase WT PA14 cells (Figure 1C, Table S1). The zeta potentials of RSCV_1 and RSCV_2 were significantly more negative as compared to BF cells and ECM cells, respectively. In contrast, RSCV_3 (−17.81 ± 0.76 mV) had a less negative zeta potential as compared to SP cells (−23.42 ± 2.93 mV) (Figure 1C, Table S1). Zeta potentials of the RSCVs and WT cells were also measured during the mid-log phase of growth. A similar trend was observed in the mid-log phase cells with the mid-log phase RSCVs having a significantly more negative surface charge than both stationary phase and mid-log phase WT PA14 (Figure S5). The hydrodynamic radii of the RSCVs were significantly larger compared to stationary phase WT PA14 (256.78 ± 44.48 nm) (Figure 1D, Table S1). When comparing cell sizes that were isolated, the hydrodynamic radii of RSCV_1 cells (377.83 ± 22.71 nm) were significantly smaller than BF cells (502.90 ± 43.14 nm), and the hydrodynamic radii of RSCV_3 (439.29 ± 15.67 nm) was significantly smaller than SP cells (127.52 ± 13.65 nm) (Figure 1D, Table S1). However, the hydrodynamic radius of RSCV_2 (428.78 ± 47.89 nm) was not significantly different from the WT ECM cells (Figure 1D, Table S1). When comparing the hydrodynamic radii of the RSCVs to each other, RSCV_1 was observed to have a lower hydrodynamic radius (377.83 ± 22.71 nm) than RSCV_3 (439.29 ± 15.67 nm) (Figure 1D, Table S1).

### 3.2. Altered Amounts of the Signaling Molecule Cyclic-Di-GMP Between the RSCVs and WT PA14

**Figure 2:**
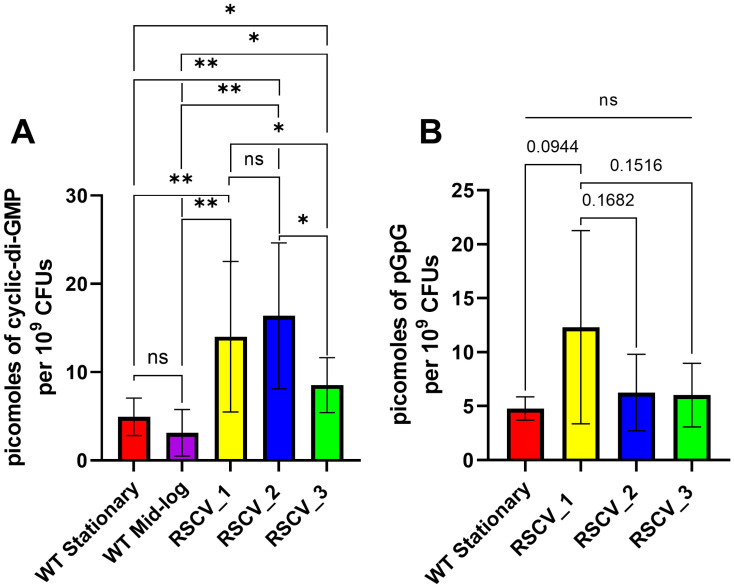
A comparison of the amounts of cyclic-di-GMP and pGpG quantified in stationary WT PA14, RSCV_1, RSCV_2, and RSCV_3. RSCV_3 has significantly reduced cyclic-di-GMP levels compared to RSCV_2 and RSCV_3. (**A**) The quantification of intracellular levels of cyclic-di-GMP normalized per colony forming unit (CFU) for stationary WT, mid-log WT, RSCV_1, RSCV_2, and RSCV_3 (n = 4–14). Picomoles of cyclic-di-GMP per 10^9^ CFUs represented on the *y*-axis. (**B**) The quantification of the intracellular levels of 5′-phosphoguanylyl-(3′,5′)-guanosine (pGpG) normalized per colony forming unit (CFU) for stationary WT PA14, RSCV_1, RSCV_2, and RSCV_3 (n = 6–7). Picomoles of pGpG per 10^9^ CFU are reported on the *y*-axis. Welch’s two-tailed unpaired t-tests were performed for all statistical analyses shown. **, *p* < 0.01, *, *p* < 0.05. A comparison with “ns” means a non-significant difference. See values on Table S2.

A defining aspect of RSCVs is the increased levels of the intracellular signaling molecule cyclic-di-GMP [10,20]. The levels of cyclic-di-GMP in the RSCVs and the stationary phase WT PA14 were quantified. The cyclic-di-GMP levels were normalized to the number of colony forming units (CFUs) for each sample. The non-naturally occurring cyclic nucleotide, 8-bromoadenosine-3′,5′-cyclic monophosphate (Br-cAMP), was used as an internal standard to quantify the amount of sample lost during the isolation. All three RSCVs had significantly increased levels of cyclic-di-GMP compared to stationary phase WT PA14 and mid-log phase WT PA14 (Figure 2A, Table S2). The quantified amount of cyclic-di-GMP in RSCV_3 was significantly lower than both RSCV_1 and RSCV_2 (Figure 2A, Table S2). Additionally, the primary metabolite of cyclic-di-GMP, 5′-phosphoguanylyl-(3′,5′)-guanosine (pGpG) [64], was also quantified in the same samples. RSCV_1 appeared to have higher levels of pGpG than RSCV_2 and RSCV_3 but was not statistically significant due to the high variability between biological replicates for RSCV_1 (Figure 2B, Table S2). No significant differences were detected in pGpG levels in any of the WT or RSCV samples (Figure 2B, Table S2).

### 3.3. RSCVs and WT PA14 Had Differing Levels of Biofilm Formation, Exopolysaccharide Production, and Antibiotic Tolerance

**Figure 3:**
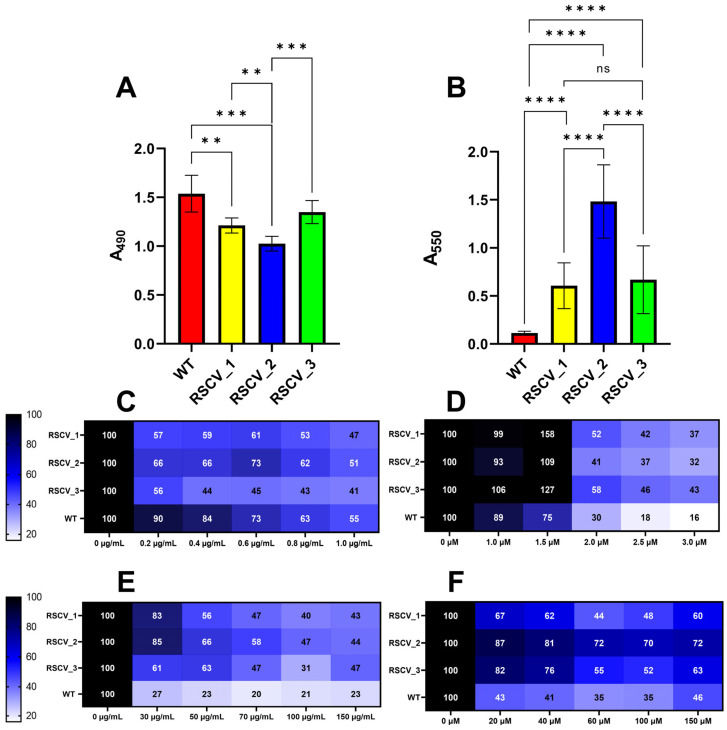
Polysaccharide, biofilm formation, and biofilm antibiotic tolerance quantifications of the three RSCVs and stationary WT PA14. RSCV_2 has the highest exopolysaccharide production and biofilm formation among the RSCVs. (**A**) Congo red measurements at 490 nm for stationary WT PA14, RSCV_1, RSCV_2, and RSCV_3 (n = 5–22). (**B**) Crystal violet 24 h biomass accumulation assay of stationary WT PA14, RSCV_1, RSCV_2, and RSCV_3 (n = 18). The observed values are in Table S3. (**C**) Crystal violet biomass accumulation assay using the antibiotic colistin for stationary WT PA14, RSCV_1, RSCV_2, and RSCV_3. Concentrations shown on the *x*-axis are in µg/mL. (**D**) Crystal violet biofilm accumulation assay using the antibiotic tobramycin for stationary WT PA14, RSCV_1, RSCV_2, and RSCV_3. Concentrations shown on the *x*-axis are in µM. (**E**) Crystal violet biofilm dissociation assay using the antibiotic colistin for stationary WT PA14, RSCV_1, RSCV_2, and RSCV_3. Concentrations shown on the *x*-axis are in µg/mL. (**F**) Crystal violet biofilm dissociation assay using the antibiotic tobramycin for stationary WT PA14, RSCV_1, RSCV_2, and RSCV_3. Concentrations shown on the *x*-axis are in µM. The number of biological replicates for the crystal violet accumulation and dissociation assays is five or six. Numbers in each cell represent the percent remaining biomass relative to the amount of biomass with no antibiotic. Welch’s two-tailed unpaired t-tests were used to calculate statistical significance for the Congo red and crystal violet assays. ****, *p* < 0.0001; ***, *p* < 0.001, **, *p* < 0.01. A comparison with “ns” means a non-significant difference.

RSCVs are hyper-biofilm producers that overproduce exopolysaccharides as compared to WT cells [20,21]. Exopolysaccharide production was quantified with the Congo red assay [38]. RSCV_2 had the highest levels of exopolysaccharide production when compared to stationary WT PA14, RSCV_1, and RSCV_3 (Figure 3A, Table S3). In contrast, RSCV_3 produced significantly less exopolysaccharides than RSCV_2 and did not significantly make more exopolysaccharides than stationary phase WT PA14 (Figure 3A, Table S3). Exopolysaccharide production was also quantified when the cells were propagated using M9 minimal media. Similar differences were observed when using M9 minimal media, with RSCV_2 still being the highest exopolysaccharide producer (Figure S6). This was further supported by the scanning-electron-microscopy (SEM) images of the three RSCVs and WT PA14. The SEM images appeared to show RSCV_2 with the highest number of visible exopolysaccharides (Figure S7). The accumulation of biomass of the RSCVs and WT PA14 was quantified by crystal violet staining (Figure 3B, Table S3) [37]. All of the RSCVs produced significantly more biomass than stationary phase WT PA14, and RSCV_2 produced more biofilms than RSCV_1 and RSCV_3 (Figure 3B, Table S3). The levels of biomass accumulation were not significantly different between RSCV_1 and RSCV_3 (Figure 3B, Table S3).

To evaluate whether the RSCVs react differently to antibiotic treatment, colistin and tobramycin were given separately to each RSCV in the absence or presence of biomass (Figure 3C–F and Figures S8 and S9) [29,39]. Colistin and tobramycin were administered to the three RSCVs and WT PA14 at the start of the crystal violet biomass accumulation assay (Figure 3C,D and Figure S8) [39]. Surprisingly, stationary WT PA14 accumulated significantly more biomass than all three RSCVs at 0.2 µg/mL and 0.4 µg/mL concentrations of colistin (Figure 3C and Figure S8A). All three RSCVs accumulated significantly more biomass at the 1.5 µM concentration of tobramycin than stationary phase WT PA14 (Figure 3D and Figure S8B). Biomass accumulation differences were also detected between the RSCVs. RSCV_2 accumulated significantly more biomass than RSCV_3 at 0.4 µg/mL, 0.6 µg/mL, and 0.8 µg/mL concentrations of colistin (Figure 3C and Figure S8A). When tobramycin when used instead of colistin, RSCV_1 accumulated significantly more biomass than RSCV_2 and RSCV_3 at 1.5 µM concentration of tobramycin (Figure 3D and Figure S8B). During the antibiotic biomass dissociation assays, all of the RSCVs were more tolerant to nearly all tested concentrations of colistin- and tobramycin-induced biofilm dispersion than WT PA14 (Figure 3E,F and Figure S9). When colistin-induced biomass dispersion was performed, RSCV_3 had significantly more dispersed biomass than both RSCV_1 and RSCV_2 at 30 µg/mL (Figure 3E and Figure S9A). Additionally, RSCV_2 was observed to be the most tolerant to tobramycin-induced biomass dispersion compared to RSCV_1 and RSCV_3 (Figure 3F and Figure S9B). RSCV_2 was significantly more tolerant to biomass dispersion than RSCV_1 at nearly all tested tobramycin concentrations (Figure 3F and Figure S9B). RSCV_2 was also more tolerant to biomass dispersion than RSCV_3 at 60 µM and 100 µM concentrations of tobramycin (Figure 3F and Figure S9B).

### 3.4. Bacterial Motility Differs Among the RSCVs

**Figure 4:**
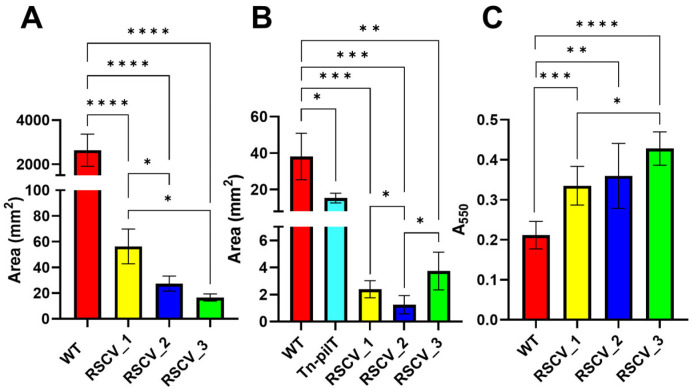
Swarming, twitching, and four-hour crystal violet assay results for RSCV_1, RSCV_2, RSCV_3, and stationary WT PA14. RSCV_3 had the lowest swarming motility and the fastest biofilm initiation. (**A**) ImageJ analysis of 0.25% agar swarming area for stationary WT, RSCV_1, RSCV_2, and RSCV_3 (n = 3–4). (**B**) Twitching motility assay results for stationary WT PA14, Tn-pilT mutant, RSCV_1, RSCV_2, and RSCV_3 (n = 3–5). (**C**) Four-hour crystal violet assay of stationary WT PA14, RSCV_1, RSCV_2, and RSCV_3 (n = 5–6). Welch’s two-tailed unpaired t-tests were performed for all statistical analyses shown. ****, *p* < 0.0001; ***, *p* < 0.001, **, *p* < 0.01, *, *p* < 0.05. The observed values are found in Table S4.

The cell motility of the RSCVs and stationary phase WT PA14 was studied through swarming and twitching LB agar assays [31,34,35]. Swarming motility is primarily a result of flagellum-mediated movement as well as secreted rhamnolipids and type IV pili-mediated movement [34]. On the other hand, twitching motility is primarily caused by type IV pili-mediated movement [34]. As a control for the twitching motility, a transposon mutant (Tn-*pilT*) containing an inserted transposable sequence in the gene that encodes for the twitching motility protein PilT (PA14_05180) was utilized [36]. Swarming was initially conducted at 0.4% (*w*/*v*) agar, and no swarming was observed for the RSCVs. Visible swarming of the RSCVs occurred at 0.25% agar (Figure S10). All of the RSCVs swarmed and twitched significantly less than stationary phase WT PA14 (Figure 4A,B and Figures S10 and S11, Table S4). Differences in the quantified swarming areas were also observed between the RSCVs. RSCV_1 had a significantly higher swarming motility than stationary phase WT PA14 (Figure 4A and Figure S10, Table S4). RSCV_3 displayed the lowest swarming motility but did not statistically differ from RSCV_2 (Figure 4A and Figure S10, Table S4). RSCV_2 also exhibited the lowest twitching motility area when compared to RSCV_1 and RSCV_3 (Figure 4B and Figure S11, Table S4).

To test for differences in biofilm initiation, after four hours of growth, biomass was stained with crystal violet [37]. Similar to previous biomass accumulation assays, all three of the RSCVs had more biomass adhered to the 96-well plate after four hours than stationary WT PA14 (Figure 4C, Table S4). Additionally, RSCV_3 had significantly more surface attached biomass compared to RSCV_1 after four hours (Figure 4C, Table S4).

### 3.5. RSCVs Were Found to Have Differing Amounts of Released Phenazine and Siderophore Virulence Factors

**Figure 5:**
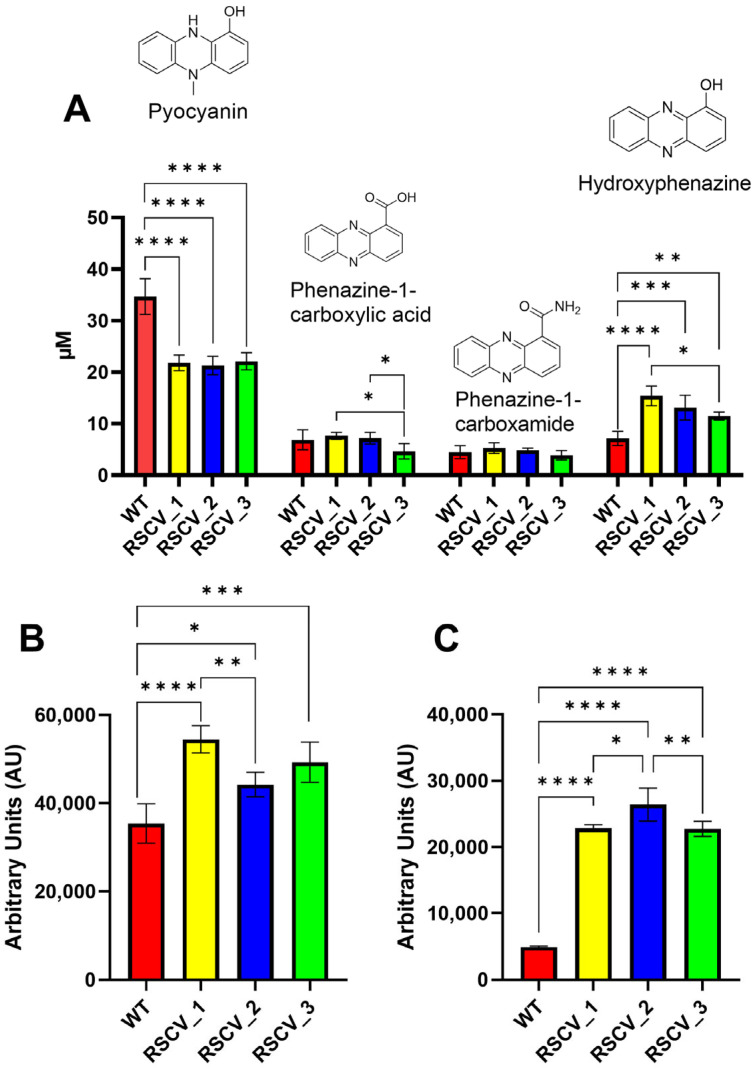
The quantification of four different phenazine and two siderophore molecules in the supernatant of stationary WT PA14 and the three RSCVs. (**A**) The quantification of pyocyanin, phenazine-1-carboxylic acid, phenazine-1-carboxamide, and hydroxyphenazine (left-to-right) in all three RSCVs and stationary WT PA14. Five or six biological replicates for each. (**B**) The quantification of the pyochelin in the supernatant of stationary WT, RSCV_1, RSCV_2, and RSCV_3 (n = 4–5). (**C**) The quantification of the pyoverdine in the supernatant of stationary WT PA14, RSCV_1, RSCV_2, and RSCV_3 (n = 4–6). Ordinary one-way ANOVA with Tukey’s multiple comparisons test used for all statistical analyses shown. ****, *p* < 0.0001; ***, *p* < 0.001, **, *p* < 0.01, *, *p* < 0.05. The observed values are in Tables S5 and S6.

Phenazines and siderophores were quantified from the supernatant of stationary phase of WT PA14 and the RSCVs using HPLC-UV and fluorescence spectroscopy respectively (Figure 5A–C, Tables S5 and S6) [39]. All three RSCVs secreted less pyocyanin significantly as compared to WT PA14 (Figure 5A, Table S5). However, the RSCVs produced higher levels of hydroxyphenazine compared to WT PA14 (Figure 5A, Table S5). RSCV_3 released significantly reduced concentrations of phenazine-1-carboxylic acid compared to RSCV_1 and RSCV_2 (Figure 5A, Table S5). Additionally, RSCV_3 secreted significantly reduced levels of hydroxyphenazine compared to RSCV_1 (Figure 5A, Table S5). The fluorescent siderophores, pyochelin and pyoverdine, were produced more by the RSCVs than WT PA14, and significant differences were also observed between the RSCVs (Figure 5B,C, Table S6). RSCV_1 produced the highest amount of pyochelin and was significantly higher than RSCV_2 (Figure 5B, Table S6). RSCV_2 produced more pyoverdine than RSCV_1 and RSCV_3 (Figure 5C, Table S6).

### 3.6. Detection of Unique Surface Cell Ions in RSCV_1, RSCV_2, and RSCV_3

Matrix-assisted laser desorption ionization (MALDI) mass spectrometry was utilized to identify differences in small molecules on the cell surfaces of stationary phase RSCV and WT PA14 cells. Overlaid mass spectra displayed significant differences between the RSCVs, particularly in positive ionization mode (Figures S12 and S13). RSCV_2, when compared to RSCV_1 and RSCV_3, showed a greater number of uniquely detected ions in positive ionization mode between 500 and 800 *m*/*z* (Figures S12C and S13E). Ions were identified as being unique if they were present in over half of the biological replicates and not present in the other samples. Some of the ions that were uniquely detected were selected for collision-induced dissociation (CID) for putative identification (Table 1 and Figures S14–S21). Rhamnolipids, Rha-C12:2 and Rha-C14:1-C14:1, were identified as unique ions in RSCV_2 and RSCV_1, respectively (Table 1). The quinolone alkyl hydroxyquinoline (AQNO) C6 was putatively identified in RSCV_1, and 2-alkyl-4-hydroxyquinoline (AHQ) C12:1 was identified in RSCV_2 (Table 1). The remaining ions were identified as two phospholipids and one lysophospholipid in negative mode (Table 1).

### 3.7. RNA Sequencing (RNA-Seq) Analysis Reveals Differentially Expressed Genes to Stationary Phase WT PA14 and Between RSCV_1 and RSCV_3

**Figure 6:**
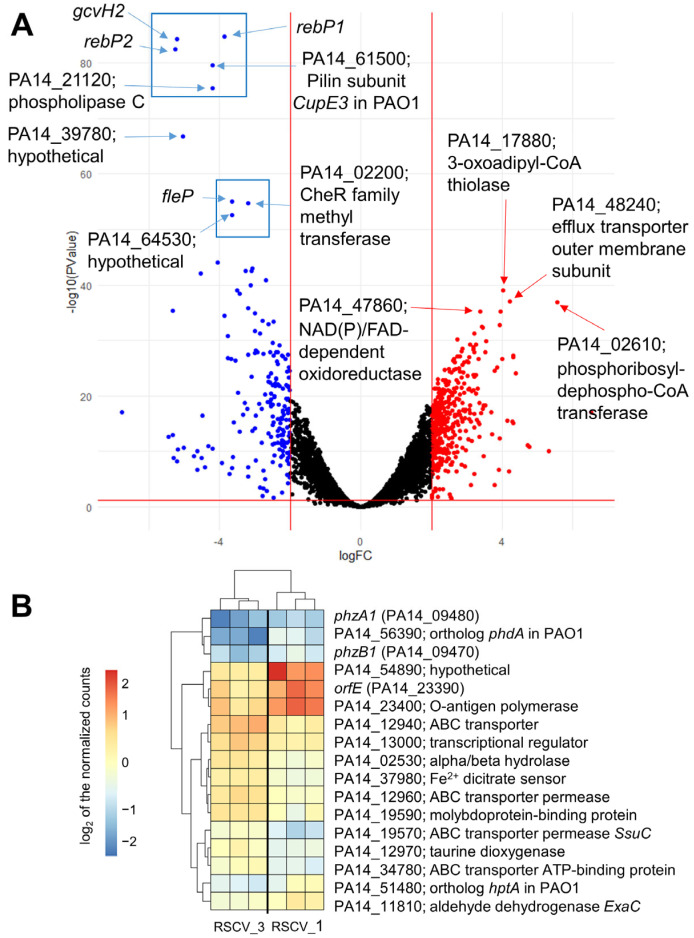
RNA sequencing (RNA-seq) analysis of stationary WT PA14, RSCV_1, and RSCV_3. The analysis of the top differentially expressed genes between RSCV_1, RSCV_3, and WT PA14 reveals differentially expressed genes related to biofilm formation, exopolysaccharide production, and antibiotic tolerance. (**A**) The volcano plot for the RNA-seq analysis of RSCV_1 and RSCV_3 as compared to stationary WT PA14. Upregulated transcripts are highlighted in red, and downregulated transcripts are highlighted in blue. Red lines are indicative of the statistical significance thresholds of *p*-values less than 0.05 and log2-fold-change greater than or equal to 2. (**B**) The heat map of the normalized raw counts for the top seventeen differentially expressed genes between RSCV_1 and RSCV_3. Scale bar is normalized to be centered around zero.

RNA-seq was performed on stationary phase WT PA14, RSCV_1, and RSCV_3. Over two thousand transcripts were identified as either upregulated or downregulated when comparing RSCV_1 and RSCV_3 to WT PA14 (Figure 6A and Supplemental Files S1 and S2). Clustering analyses of the normalized transcript levels displayed distinct clustering between RSCV_1 and RSCV_3 to WT PA14 (Figures S22 and S23). The KEGG (Kyoto Encyclopedia of Genes and Genomes) pathway analysis identified the gene pathways containing the highest number of differentially expressed genes (Figure S24). The ribosome, carbon metabolism, and flagellar assembly were some of the gene pathways with the most downregulated transcripts in the two RSCVs compared to WT PA14 (Figure S24B). In contrast, the biosynthesis of cofactors, the bacterial secretion system, and quorum sensing were among the gene pathways with the most upregulated transcripts (Figure S24A,C). The differentially expressed genes of the RSCVs were consistent with the known hyper-biofilm and low motility phenotypes of *P. aeruginosa* RSCVs [19,20,21,22]. These include the upregulation of genes related to biofilm formation and the downregulation of flagellar and pili motility pathway genes. For example, the flagellar basal body rod protein FlgG (PA14_50480) and the type IV pilin Flp (PA14_55940) were both significantly downregulated in the RSCVs (Supplemental Files S1–S3). Additionally, most of the *pel* operon, which is responsible for the biosynthesis of the polysaccharide Pel [65], was significantly upregulated in the RSCVs (Supplemental Files S1–S3). Some of the most upregulated genes were involved in the multidrug efflux pump systems (Supplemental Files S1–S3). An outer membrane component of the multidrug efflux pump (PA14_48240) was one of the most upregulated genes (Figure 6A, Supplemental Files S1–S3). Additionally, metabolism-related genes were among the most upregulated transcripts in the RSCVs (Supplemental Files S1–S3). A phosphoribosyl-dephospho-CoA transferase (PA14_02610) and a NAD(P)/FAD-dependent oxidoreductase (PA14_47860) were two of the most significantly upregulated genes (Figure 6A, Supplemental Files S1–S3). In contrast, some of the most significantly downregulated transcripts in the RSCVs were *rebP1* (PA14_27640), *rebP2* (PA14_27630), *gcvH2* (PA14_32985), a Csu type fimbrial protein (PA14_61500) PA14_02200 (CheR family methyltransferase), the pili length/flagellar attachment protein FleP (PA14_50240), phosphocholine-specific phospholipase C (PA14_21120), and two hypothetical proteins (Figure 6A, Supplemental Files S1–S3). Out of the 347 predicted transcriptional regulators in the *Pseudomonas* Genome Database [66], 152 transcriptional regulators were significantly differentially expressed between both of the RSCVs and stationary WT PA14 (Supplemental Files S1–S3). Some of the most significantly differentially expressed transcriptional regulators were *mvfR* (PA14_51340) and *mvaT* (PA14_56070), which were both downregulated in RSCV_1 and RSCV_3 compared to stationary WT PA14 (Supplemental Files S1–S3). Overall, the most significantly upregulated and downregulated transcripts match the expected phenotypic characteristics of RSCVs, including increased antibiotic tolerance and biofilm formation and drastically decreased bacterial motility [19,20].

RNA-seq analysis comparing RSCV_1 to RSCV_3 showed a total of seventy-five differentially expressed genes (Figure S25 and Supplemental File S3). Among the most significantly differentially expressed genes are the phenazine biosynthesis proteins *phzA1* (PA14_09470) and *phzB1* (PA14_09480), a polysaccharide biosynthesis protein *orfE* (PA14_23390), an Hpt domain-containing protein (PA14_51480), and an acetyltransferase family protein (PA14_56390) (Figure 6B, Supplemental Files S4 and S5). The gene expression levels of the phenazine biosynthesis proteins, *phzA1* and *phzB1*, were both downregulated in RSCV_3 compared to RSCV_1 (Figure 6B, Supplemental Files S4 and S5). The expression levels of *orfE*, the O-antigen polymerase PA14_23400, PA14_51480, and PA14_56390 were all upregulated in RSCV_1 compared to RSCV_3 (Figure 6B, Supplemental Files S4 and S5).

Genes within the cyclic-di-GMP signaling pathway were analyzed to investigate differences between RSCV_1 and RSCV_3 [67]. No genes known to be involved in cyclic-di-GMP signaling were significantly differentially expressed between RSCV_1 and RSCV_3. To investigate minor changes in cyclic-di-GMP signaling, RSCV_1 and RSCV_3 were compared individually to WT PA14. The log-fold-change of each RSCV to WT PA14 were compared to each other. Transcriptional differences related to cyclic-di-GMP signaling were observed between RSCV_1 to WT PA14 and RSCV_3 to WT PA14 (Figure S26) [67]. The largest cyclic-di-GMP-related transcriptional changes were three predicted phosphodiesterases and two putative diguanylate cyclases. These three cyclic-di-GMP phosphodiesterases are PA14_21190 (an ortholog of *nbdA* (PA3311) in PAO1), the EAL domain-containing protein PA14_36990, and *pvrR* (PA14_59790) (Figure S26, Supplemental Files S6–S9) [66,68]. RSCV_1 had a lower expression of PA14_21190 and PA14_36990 compared to WT PA14 than RSCV_3 compared to WT PA14 (Figure S26, Supplemental Files S6–S9). PA14_59790 appeared to be more expressed in RSCV_1 compared to WT PA14 than RSCV_3 to WT PA14 (Figure S26, Supplemental Files S6–S9). The two diguanylate cyclases, containing only GGDEF protein domains, were both observed to have a higher expression in RSCV_1. These two diguanylate cyclases were a GGDEF domain-containing response regulator (PA14_57140) and a GGDEF-only-containing sensory box protein (PA14_23130) (Figure S26, Supplemental Files S6–S9).

### 3.8. Whole-Genome Single-Molecule Real-Time (SMRT) Sequencing of Stationary WT PA14, RSCV_1, RSCV_2, and RSCV_3 Revealed Unique Single-Nucleotide Polymorphisms (SNPs)

Genomic SMRT sequencing and sequence alignments of the three RSCVs to stationary phase WT PA14 detected a number of DNA deletions and single-nucleotide polymorphisms (SNPs). Commonly detected DNA mutations in *P. aeruginosa* RSCVs are mutations in the Wsp pathway operon [23,28]. In our RSCVs, similar Wsp DNA mutations were detected. All of the RSCVs had a single adenine-to-guanosine DNA substitution in *wspF* (genomic base-pair location of 1,412,866) compared to WT PA14 (Tables S7–S9). Another shared DNA mutation detected in all three RSCVs was in PA14_61850 (Tables S7–S9). PA14_61580 is an ortholog of *chtA* in PAO1 (PA4675) and is an outer-membrane receptor for the transport of the xenosiderophore aerobactin [69,70]. Additionally, unique mutations were detected in the Wsp system of RSCV_1 and RSCV_2. RSCV_1 was the only RSCV that had a cytosine-to-thymine DNA substitution in *wspA* (Table 2). RSCV_2 had an additional SNP in *wspF* that was not present in the other RSCVs, which corresponded to a cytosine-to-thymine substitution (Table 2).

Other DNA mutations were detected in the RSCVs that were not related to the Wsp system (Table 2). All of the RSCVs contained several mutations in type IV pili- and flagellum-related genes (Table 2 and Table S10). RSCV_3 was the only RSCV that contained deletions in the gene that encodes for PhzB1 (PA14_09470) (Table 2). Another single-base deletion caused a frameshift in a penicillin binding protein (PA14_62200) in RSCV_1 (Table 2). This resulted in the addition of a stop codon near the beginning of the gene. Additionally, multiple deletions in RSCV_3 caused a frameshift in the motility regulator *morA* (PA14_60870). In the amino-acid alignment of the mutated MorA protein, an insertion of a stop codon prior to the GGDEF domain but after the EAL domain was observed (Figure S27).

## 4. Discussion

*Pseudomonas aeruginosa* biofilm subpopulations were previously isolated from a PA14 biofilm [29]. These biofilm subpopulations were identified to be transient, as they were observed to revert back to stationary phase phenotypes when re-grown after plating [29]. Small colony variants were observed to isolate with the transient biofilm subpopulations. The small colony variants were initially suspected to be RSCVs due to their small and wrinkled colony morphologies (Figure S2). It was essential to confirm that the isolated small colony variants were distinct from the biofilm subpopulations. It has been previously noted that small colony variants typically display a colony size roughly one-tenth of WT colonies [71]. This was also true for our RSCVs. Our WT PA14 colony diameters were approximately ten times the colony diameters of RSCV_1, RSCV_2, and RSCV_3 (Figure 1A). While all of the WT biofilm subpopulations had slightly smaller colony diameters than stationary WT PA14, they had significantly larger colony diameters than any of the RSCVs (Figure 1A). Changes in colony morphology were also observed to exist between the RSCVs and the biofilm subpopulations. All of the RSCVs were hyper-wrinkled, irregular colony form and had crateriform colony elevations (Figure S2) [29,62,63]. In contrast, none of the biofilm subpopulations had crateriform colony elevations (Figure S3) [29,62,63]. Significant differences in the cell surface charges and hydrodynamic radii were also observed between the RSCVs and the biofilm subpopulations (Figure 1C,D). All of this evidence suggests that the isolated RSCVs were in fact distinct from the BF, ECM, and SP biofilm subpopulations. The isolated RSCVs displayed known RSCV phenotypes [19,22]. The RSCVs have decreased colony diameters and growth rates (Figure 1A,B). The RSCVs also had higher cellular hydrodynamic radii, exopolysaccharide production, cyclic-di-GMP production, and biofilm formation compared to WT PA14 (Figure 1D, Figure 2A and Figure 3A,B). While a higher hydrodynamic radius is not typically associated with RSCVs, the increase in the hydrodynamic radii of our isolated RSCVs to WT is similar to the RSCVs from the Gram-positive bacteria *Enterococcus faecalis* (Figure 1D) [72]. The analysis of the RNA and DNA sequencing of stationary phase RSCV_1, RSCV_2, RSCV_3, and WT PA14 also supported the existence of many of the known RSCV phenotypes. For example, the entire Pel operon (*pelA*/PA14_24480–*pelG*/PA14_24560) was upregulated in RSCV_1 and RSCV_3 compared to WT PA14 (Supplemental Files S1–S3). Additionally, a number of motility-related genes were downregulated in RSCV_1 and RSCV_3, with one of the most downregulated gene pathways being flagellar assembly (Figure S24B, Supplemental Files S1–S3). The DNA sequencing analysis confirmed the existence of an identical DNA mutation in wspF for all three RSCVs (Tables S7–S10). Mutations in *wspF* have been associated with the RSCV phenotype by locking the *wspR* diguanylate cyclase into a permanently active state [19,22,73]. Furthermore, all three of the RSCVs had mutations in type IV pili-related genes (Table 2 and Table S10). This matches the RSCV phenotype, as RSCVs are known to be deficient in twitching motility [23,74].

Significant differences were noticed between the RSCVs themselves. One of the most important differences was in the intracellular levels of cyclic-di-GMP. While RSCV_1 and RSCV_2 had similar levels of cyclic-di-GMP, RSCV_3 produced significantly less cyclic-di-GMP compared to RSCV_1 and RSCV_2 (Figure 2A). Cyclic-di-GMP is a signaling molecule known to regulate the transition from motile planktonic cells to biofilm cells and exopolysaccharide production [10,26]. Thus, the reduced levels of cyclic-di-GMP, exopolysaccharide production, biofilm formation, and swarming motility of RSCV_3 compared to RSCV_2 are consistent (Figure 2A, Figure 3A,B and Figure 4A). The explanations for the reduced levels of cyclic-di-GMP in RSCV_3 are the two DNA mutations unique to RSCV_3 in the *morA* (PA14_60870) gene. *MorA* encodes for a motility regulator protein that contains both a diguanylate cyclase motif (GGDEF) and a phosphodiesterase motif (EAL). It has been determined to be a bi-functional cyclic-di-GMP regulator with enzymatic assays experimentally showing both diguanylate cyclase and phosphodiesterase activity [75]. These two single-nucleotide deletions in morA result in a frameshift mutation that causes a stop codon to be introduced between the EAL and GGDEF amino acid domains, resulting in the loss of the diguanylate cyclase motif (Figure S27). This likely results in the loss of diguanylate cyclase activity but not phosphodiesterase activity. RSCV_2, which was by far the highest biofilm-producing variant (Figure 3B), displayed the highest hyper-biofilm-associated phenotypes compared to the other RSCVs. RSCV_2 was the highest exopolysaccharide producer, which is an essential component of the extracellular matrix, and had the lowest twitching motility (Figure 3A and Figure 4B).

The gene expression analysis corroborated many of the cyclic-di-GMP and phenotypic changes seen in the RSCVs. Analyzing cyclic-di-GMP-related transcripts revealed differences between RSCV_3 and RSCV_1 (Figure S26). Two putative diguanylate cyclases, a GGDEF domain containing a two-component response regulator (PA14_57140), and a sensory box protein containing only a GGDEF motif (PA14_23130) appeared to be less expressed in RSCV_3 to WT PA14 than RSCV_1 to WT PA14 (Figure S26). Additionally, three predicted cyclic-di-GMP-specific phosphodiesterases (PA14_21190, PA14_36990, and PA14_59790) were observed to have a higher expression in RSCV_3 than RSCV_1 when both were compared to WT PA14 (Figure S26, Supplemental Files S6–S9). This suggests that transcriptional changes could also be causing the decreased production of cyclic-di-GMP observed in RSCV_3. Furthermore, RSCV_3 was the lowest exopolysaccharide producer compared to the other RSCVs, which was also supported by the RNA-seq analysis (Figure 3A and Figure 6B). The polysaccharide biosynthesis protein *orfE* (PA14_23390) and the O-antigen polymerase PA14_23400 were downregulated in RSCV_3 compared to RSCV_1 (Figure 6B). PA14_23400 is predicted to be responsible for O-antigen polysaccharide biosynthesis [66]. Additionally, both *orfK* (PA14_23370) and *orfH* (PA14_23380), which are a predicted UDP-N-acetyl-D-glucosamine 6-dehydrogenase and a UDP-N-acetyl-D-mannosaminuronate dehydrogenase, respectively, are directly involved in polysaccharide biosynthesis and had lower gene expression levels in RSCV_3 compared to RSCV_1 (Supplemental Files S4 and S5) [66,76]. In the RNA-seq analysis, we detected 152 transcriptional regulators that were differentially expressed between RSCV_1 and RSCV_3 to WT PA14. (Supplemental File S2) [66]. Of these 152 differentially expressed transcriptional regulators, 138 were significantly upregulated in the RSCVs compared to WT PA14. Among the detected 14 downregulated transcriptional regulators were *mvfR* (PA14_51340) and *mvaT* (PA14_56070) (Supplemental File S2). *MvfR* has been shown to be involved in the regulation of multiple quorum sensing genes in *P. aeruginosa* [77]. Furthermore, a genetic knockout of *mvaT* caused an increase in pyocyanin production and antibiotic tolerance through the regulation of multidrug efflux systems [78]. The decreased levels of pyocyanin in the RSCVs as compared to WT PA14 (Figure 5A) could be due to the lowered expression of the virulence factor *vfr* (PA14_08370) (Supplemental File S2). Cyclic-di-GMP has been reported to inhibit *vfr* and is believed to cause the lower acute virulence factor production of RSCVs [79]. The differential expression of quorum sensing and virulence-related regulators helps to explain the high variation between the specific types of virulence factors (Figure 5A,B). Other RSCVs are known to undergo metabolic changes that result in changes in secondary metabolite production [19,71].

The RSCVs secreted secondary metabolites in different levels. While the virulence of many other small colony variants and RSCVs are lower than WT cells [80,81], certain strains of *P. aeruginosa* small colony variants have been shown to have increased virulence compared to WT [82]. The reported higher concentrations of several virulence factors in RSCV_1, RSCV_2, and RSCV_3 compared to stationary phase WT PA14, therefore, is still consistent with the scientific literature (Figure 5A–C). The RSCVs were observed to have higher concentrations of hydroxyphenazine, pyochelin, and pyoverdine virulence factors than stationary phase WT PA14 (Figure 5A–C). While virulence was never directly measured, the overall increase in virulence factor production suggests that the RSCVs are more virulent than *P. aeruginosa* WT PA14 cells. Virulence-related changes were also observed between the RSCVs. RSCV_3 produced less phenazine-1-carboxylic acid compared to RSCV_1 and RSCV_2 (Figure 5A). RSCV_3 made less hydroxyphenazine than RSCV_1 (Figure 5A). As phenazines have been implicated in pathogenicity in animal models and crucial for lung infection in mice, the reduced concentrations of phenazines detected in RSCV_3 implies reduced virulence compared to RSCV_1 and RSCV_2 [83,84]. The reduced phenazine production in RSCV_3 could be attributed to the downregulation of two phenazine biosynthesis proteins compared to RSCV_1: *phzA1* (PA14_ 09480) and *phzB1* (PA14_09470) (Figure 6B). In the DNA sequencing analysis, RSCV_3 was the only RSCV that had a single-base DNA deletion in *phzB1* (Table 2). The swarming motility assay was also consistent with RSCV_3 having reduced phenazine production (Figure 4A). Increases in swarming motility have been linked to an increase in virulence in many bacterial species such as *Salmonella typhimurium* and *Proteus mirabilis* [85,86]. In *P. aeruginosa*, a previously published RNA-seq analysis of cells with increased swarming showed the upregulation of many phenazine-related genes [87]. Therefore, RSCV_3 having lower swarming motility and phenazine production is consistent (Figure 4A and Figure 5A).

Antibiotic tolerance differences were observed between WT PA14, RSCV_1, RSCV_2, and RSCV_3 (Figure 3C–F and Figures S8 and S9). The RSCVs accumulated more biomass in the presence of tobramycin as compared to WT PA14 at all tested concentrations (Figure 3D). Additionally, it was more difficult to disperse biomass in the RSCVs with colistin and tobramycin compared to WT PA14 (Figure 3E,F). This data is supported by previously reported studies that have found similar increases in the antibiotic tolerance of *P. aeruginosa* RSCVs [88,89]. Consistent with the antibiotic assays, two of the most upregulated genes in RSCV_1 and RSCV_3 compared to WT PA14 were the multidrug efflux system proteins PA14_48240 and PA14_38395 (Figure 6A and Supplemental File S2). Since the elevated levels of cyclic-di-GMP result in the increased gene expression of multidrug efflux pumps [90,91], higher cyclic-di-GMP levels could have increased the expression of these two multidrug efflux system genes. Additional antibiotic tolerance changes were observed between the RSCVs. Overall, RSCV_2 is more antibiotic tolerant than the other RSCVs. In the presence of colistin, RSCV_2 accumulated more biomass, and the biomass produced by RSCV_2 was harder to disperse with colistin and tobramycin than the other RSCVs (Figure 3C–F and Figures S8 and S9). Interestingly, the antibiotic tolerance varied between RSCV_1 and RSCV_2 depending on which antibiotic was used. While it was not significantly harder to disperse RSCV_2 than RSCV_1 with colistin (Figure S9A), RSCV_2 was significantly more difficult to disperse with tobramycin than RSCV_1 (Figure S9B).

To study the heterogeneous cells of a *P. aeruginosa* biofilm, we separated and isolated three distinct subpopulations and three RSCVs. The isolated RSCVs were significantly different than the WT biofilm subpopulations in terms of colony diameter, growth rates, cell surface charge, hydrodynamic radii, and colony morphology (Figure 1A–D and Figures S2 and S3). The three RSCVs showed significant differences to one another in terms of antibiotic tolerance; biofilm formation; and the production of cyclic-di-GMP, exopolysaccharides, phenazines, and siderophores (Figure 2A, Figure 3A–F, Figure 5A–C and Figure S6). The production of phenazines was inconsistent compared to RSCVs and WT PA14 cells, but siderophore production was significantly higher in all of the RSCVs compared to WT PA14. This could suggest that the RSCVs have increased virulence compared to WT PA14. While it is commonly accepted that increased levels of cyclic-di-GMP causes many of the RSCV phenotypes, very little research has investigated the differences in cyclic-di-GMP between RSCVs derived from the same biofilm. RSCV_3 displayed the most differences among the three RSCVs with the lowest intracellular levels of cyclic-di-GMP, exopolysaccharide and phenazine production, and swarming motility (Figure 2A, Figure 3A, Figure 4A, Figure 5A and Figure S6). In contrast, RSCV_2 had the highest exopolysaccharide production, biofilm formation, and the highest overall antibiotic tolerance (Figure 3A–F and Figures S6, S8 and S9). DNA and RNA sequencing analysis revealed genetic and transcriptional changes between the RSCVs that supported many of the reported phenotypic changes (Figure 6A, Table 1, Supplemental Files S1–S9). As RSCVs have high medical relevance, with most chronic infections being attributed to RSCVs [92], insights into the differences between RSCVs from the same biofilm could prove useful in combating RSCV infections.

## Figures and Tables

**Table 1:** Table of identified unique ions from MALDI CID for positive and negative ionization modes.

Ionization Mode	Unique Ion	Variant	Molecule	Exact Mass	Adduct
Positive	290.11 *m*/*z*	RSCV_1	AQNO C_6_	245.14	[M + 2Na − H]
	381.09 *m*/*z*	RSCV_2	Rha-C_12:2_	358.20	[M + Na]
	658.90 *m*/*z*	RSCV_1	Rha-C_14:1-C14:1_	612.42	[M + 2Na]
	760.45 *m*/*z*	RSCV_3	PE (18:1/16:1)	715.52	[M + 2Na − H]
	802.20 *m*/*z*	RSCV_2	PG (18:2/17:1)	757.50	[M + 2Na − H]
Negative	270.56 *m*/*z*	RSCV_1	AHQ C_9_	271.19	[M − H]
	350.99 *m*/*z*	RSCV_2	AHQ C_12:1_	327.22	[M + Na]
	426.16 *m*/*z*	RSCV_1	LPA (14)	381.20	[M + 2Na − H]

**Table 2:** List of relevant unique SNPs in the whole-genome DNA sequencing analysis.

Sample	Base-Pair Location (vs. WT)	SNP Type (vs. WT)	Locus Tag	Gene Name/Function
RSCV_1	1,406,791	Substitution (C to T)	PA14_16430	*wspA*
	5,373,709	Deletion	PA14_60310	*pilY1*, type 4 fimbrial biogenesis protein
	5,375,954	Deletion	PA14_60310	*pilY1*, type 4 fimbrial biogenesis protein
	5,550,044	Deletion	PA14_62200	*mrcB*, penicillin-binding protein 1B
RSCV_2	1,412,599	Substitution (C to T)	PA14_16480	*wspF*
	2,116,846	Deletion	PA14_24350	two-component response regulator
	4,622,456	Deletion	PA14_52130	Hypothetical protein; ortholog of *wzz2* in PAO1
RSCV_3	812,449	Deletion	PA14_09470	*phzB1*, phenazine biosynthesis protein
	812,449	Deletion	PA14_09470	*phzB1*, phenazine biosynthesis protein
	4,568,205	Deletion	PA14_51410	*pqsC*, quorum sensing
	5,278,506	Deletion	PA14_59250	*pilN2*, pilus assembly
	5,280,695	Deletion	PA14_59270	*pilO2*, type IV b pilus protein
	5,431,207	Deletion	PA14_60870	*morA*, motility regulator
	5,432,209	Deletion	PA14_60870	*morA*, motility regulator
	5,278,506	Deletion	PA14_59250	*pilN2*, pilus assembly

## Data Availability

The RNA-seq raw and processed data is available at the NCBI GEO Omnibus under the accession number GSE293895 (https://www.ncbi.nlm.nih.gov/geo/query/acc.cgi?acc=GSE293895, accessed on 21 October 2025). The DNA sequencing raw files have been uploaded to the NCBI GenBank database under BioProject PRJNA1244416 (accession numbers SAMN47652032, SAMN47652033, and SAMN47652034) https://www.ncbi.nlm.nih.gov/bioproject/?term=PRJNA1244416, accessed on 31 March 2025).

## Supplementary Materials

The following supporting information can be downloaded at https://www.mdpi.com/article/10.3390/microorganisms13112550/s1, Figure S1: A diagram of the isolation of RSCV_1, RSCV_2, and RSCV_3; Figure S2: Olympus light microscope images of WT PA14, RSCV_1, RSCV_2, and RSCV_3 colonies; Figure S3: Olympus light microscope images of WT BF, WT ECM, and WT SP colonies; Figure S4: An analysis of the rate of growth in M9 minimal media for RSCV_1, RSCV_2, RSCV_3, and WT PA14. Figure S5: Cell surface charge measurements for cells grown to the mid-log phase. Figure S6: Quantifications of exopolysaccharide production for the RSCVs and WT PA14 in either Luria–Bertani (LB) broth or M9 minimal media (MM). Figure S7: SEM images of RSCV_1, RSCV_2, RSCV_3, and stationary WT PA14; Figure S8: The statistical analysis of the accumulation of biomass in the presence of colistin and tobramycin; Figure S9: The statistical analysis of the dispersion of biofilms with colistin and tobramycin; Figure S10: Representative swarming images of stationary WT PA14, RSCV_1, RSCV_2, and RSCV_3 with 0.25% agar; Figure S11: Additional twitching data for RSCV_1, RSCV_2, RSCV_3, and stationary WT PA14; Figure S12: Overlaid MALDI reflectron positive ionization mode scan mass spectra of the three RSCVs and stationary WT PA14; Figure S13: Overlaid MALDI reflectron negative ionization mode scan mass spectra of the RSCVs and WT PA14 cells; Figure S14: CID-positive ionization mode fragmentation of 290 *m*/*z* in RSCV_1; Figure S15: CID-positive ionization mode fragmentation of 381 *m*/*z* in RSCV_2; Figure S16: CID-positive ionization mode fragmentation of 659 *m*/*z* in RSCV_1; Figure S17: CID-positive ionization mode fragmentation of 760 *m*/*z* in RSCV_3; Figure S18: CID-positive ionization mode fragmentation of 802 *m*/*z* in RSCV_2; Figure S19: CID-negative ionization mode fragmentation of 271 *m*/*z* for RSCV_1; Figure S20: CID-negative ionization mode fragmentation of 351 *m*/*z* for RSCV_2; Figure S21: CID-negative ionization mode fragmentation of 426 *m*/*z* for RSCV_1; Figure S22: The fuzzy clustering analysis (FCA) of the RNA-seq normalized raw counts for RSCV_1, RSCV_3, and stationary WT PA14; Figure S23: The principal component analysis of the clustered RNA-seq normalized raw counts for RSCV_1 (blue), RSCV_3 (black), and WT PA14 (purple); Figure S24: The KEGG (Kyoto Encyclopedia of Genes and Genomes) pathway analysis for stationary WT PA14 compared to RSCV_1 and RSCV_3; Figure S25: The volcano plot of the RNA-seq analysis for RSCV_1 compared to RSCV_3; Figure S26: A heat map showing the log2-fold-changes of genes known to be related to the signaling molecule cyclic-di-GMP in *P. aeruginosa*; Figure S27: The amino acid alignment for the protein MorA (PA14_60870) in WT and RSCV_3; Table S1: Observed values (average ± standard deviation, n = number of biological replicates) for the colony diameter, growth curve slope, hydrodynamic radius, and zeta potential data; Table S2: Observed values (average ± standard deviation, n = number of biological replicates) for the cyclic-di-GMP and pGpG quantification data in picomoles per 10^9^ CFUs; Table S3: Observed values (average ± standard deviation, n = number of biological replicates) for the Congo red and crystal violet data; Table S4: Observed values (average ± standard deviation, n = number of biological replicates) for swarming motility, twitching motility, and biofilm initiation; Table S5: Observed values (average ± standard deviation, n = number of biological replicates) for the phenazine quantification data; Table S6: Observed values (average ± standard deviation, n = number of biological replicates) for the pyochelin and pyoverdine quantification data; Table S7: Table of gene mutations shared between RSCV_1 and RSCV_2; Table S8: Table of gene mutations shared between RSCV_1 and RSCV_3; Table S9: Table of gene mutations shared between RSCV_2 and RSCV_3; Table S10: Full list of detected mutations in motility-related genes in RSCV_1, RSCV_2, or RSCV_3; Table S11: Selected locus tag conversions for locus tags discussed in this work; Supplemental File S1: RNA-seq normalized raw count file for RSCV_1, RSCV_3, and stationary WT PA14; Supplemental File S2: A file containing all of the differentially expressed genes between WT PA14 compared to both RSCV_1 and RSCV_3; Supplemental File S3: A file containing the full gene analysis for the stationary WT PA14 versus RSCV_1 and RSCV_3 RNA-seq comparison; Supplemental File S4: A file containing all of the differentially expressed genes between RSCV_1 and RSCV_3; Supplemental File S5: A file containing the full gene analysis for the RSCV_1 versus RSCV_3 RNA-seq comparison; Supplemental File S6: A file containing all of the differentially expressed genes between WT PA14 and RSCV_1; Supplemental File S7: A file containing all of the differentially expressed genes between WT PA14 and RSCV_3; Supplemental File S8: A file containing the full gene analysis for the stationary WT PA14 versus RSCV_1 RNA-seq comparison; Supplemental File S9: A file containing the full gene analysis for the stationary WT PA14 versus RSCV_3 RNA-seq comparison.

## Abbreviations

The following abbreviations are used in this manuscript:

RSCV	Rugose Small Colony Variant
KEGG	Kyoto Encyclopedia of Genes and Genomes
ECM	Extracellular Matrix Cells
BF	Biofilm Cells
SP	Supernatant Cells
GEO	Gene Expression Omnibus
Cyclic-di-GMP	bis-(3′,5′)-cyclic-dimeric-guanosine monophosphate
CF	Cystic fibrosis
pGpG	5′-phosphoguanylyl-(3′,5′)-guanosine
SMRT	Single-Molecule Real-Time
RNA-seq	RNA sequencing
MALDI	Matrix-Assisted Laser Desorption Ionization
CFU	Colony Forming Unit
FCA	Fuzzy Clustering Analysis
