# Supplementary material for: Identification of Three New Rugose Small Colony Variants from a Pseudomonas aeruginosa Biofilm
Source: Microorganisms. 2025 Nov 7;13(11):2550. doi: 10.3390/microorganisms13112550 (PMC12654532; doi:10.3390/microorganisms13112550)
Supplement: Supplementary file 1 [file microorganisms-13-02550-s001.zip › microorganisms-3900109-supplementary/microorganisms-3900109-supplementary.pdf]

Supporting Information

Identification of Three New Rugose Small Colony Variants from a *Pseudomonas aeruginosa* Biofilm

Benjamin K. Smartnick[1], Eric A. Carlson[1], Chase N. Morse[1], Taylor A. Dodson[1‡], Nathan C. Wamer[1§], Avery M. Horne[1], Erin G. Prestwich[1*]

[1] Department of Medicinal and Biological Chemistry, University of Toledo, Toledo, Ohio, USA 43606

* Correspondence: erin.prestwich@utoledo.edu, 419-530-1944

Current address:
‡ Saint Martin's University, Department of Natural Sciences, Lacey, WA 98530
§ Shimadzu Scientific Instruments, Inc. 243 W. Roosevelt Road, Building 7, Suite 49, Chicago, IL, 60185

Table of Contents:

Figure S1 - Diagram of the isolation for RSCV_1, RSCV_2, and RSCV_3..p. 4

Figure S2 - Olympus light microscope images of WT PA14, RSCV_1, RSCV_2, and RSCV_3 colonies……………………………………………………………………………………………………..p. 5

Figure S3 - Olympus light microscope images of WT BF, WT ECM, and WT SP colonies……………………………………………………………………………………………………..p. 6

Figure S4 - Growth Curves in M9 minimal media and LB for RSCV_1, RSCV_2, RSCV_3, and WT PA14……………………………………………………………………………………..p. 7

Figure S5 - Cell surface charge measurments for cells grown to mid-log phase………………………………..p. 8

Figure S6 - Quantifications of exopolysaccharide production for the RSCVs and WT PA14 in either Luria-Bertani (LB) broth or M9 minimal media (MM)………………………………………………p. 9

Figure S7 - SEM images of RSCV_1, RSCV_2, RSCV_3, and stationary WT PA14…………………………………………………………………………………………………...p. 10-11

Figure S8 - Statistical analysis of the accumulation of biomass in the presence of colistin and tobramycin.…………………………………………………………………………..p. 12

Figure S9 - Statistical analysis of the dispersion of biofilms with colistin and tobramycin……………………………………………………………………………………………...p. 13

Figure S10 - Representative swarming images of stationary WT PA14, RSCV_1, RSCV_2, and RSCV_3 with 0.25% agar………………………………………………………………...........................p. 14

Figure S11 - Additional twitching data for RSCV_1, RSCV_2, RSCV_3, and stationary WT PA14……………………………………………………………………………………...p. 15

Figure S12 - Overlaid MALDI reflectron positive ionization mode scans mass spectra of the three RSCVs and stationary WT PA14…………...p. 16

Figure S13 - Overlaid MALDI reflectron negative ionization mode scans mass spectra of the RSCVs and WT PA14 cells……………………………………………………………………p. 17

Figure S14 - CID negative ionization mode fragmentation of 271 *m/z* for RSCV_1……………………………………………………………………………………….............................p. 18

Figure S15 - CID negative ionization mode fragmentation of 351 *m/z* for RSCV_2……………………………………………………………………………………….............................p. 19

Figure S16 - CID negative ionization mode fragmentation of 426 *m/z* for RSCV_1……………………………………………………………………………………….............................p. 20

Figure S17 - CID positive ionization mode fragmentation of 290 *m/z* in RSCV_1……………………………………………………………………………………….............................p. 21

Figure S18 - CID positive ionization mode fragmentation of 381 *m/z* in RSCV_2……………………………………………………………………………………….............................p. 22

Figure S19 - CID positive ionization mode fragmentation of 659 *m/z* in RSCV_1………………………………………………………………………………………………………...p. 23

Figure S20 - CID positive ionization mode fragmentation of 760 *m/z* in RSCV_3……………………………………………………………………………………….............................p. 24

Figure S21 - CID positive ionization mode fragmentation of 802 *m/z* in RSCV_2……….............................p. 25

Figure S22 - Fuzzy Clustering Analysis (FCA) of the RNA-seq normalized raw counts for RSCV_1, RSCV_3, and stationary WT PA14………………………………........................p. 26

Figure S23 - Principal component analysis of the clustered RNA-seq normalized raw counts for RSCV_1 (blue), RSCV_3 (black), and WT PA14 (purple)……………………………………………p.27

Figure S24 - KEGG (Kyoto Encyclopedia of Genes and Genomes) pathway analysis for stationary WT PA14 compared to RSCV_1 and RSCV_3…………………….............................p. 28

Figure S25 - Volcano plot of the RNA-seq analysis for RSCV_1 compared to RSCV_3……………………………………………………………………………………….............................p. 29

Figure S26 - Heat map showing the $\log_2$-fold-changes of genes known to be related to the signaling molecule cyclic-di-GMP in *P. aeruginosa*..p. 30

Figure S27 - Amino acid alignment for the protein MorA (PA14_60870) in WT and RSCV_3………………………………………………………………………………………………………...p. 31

Table S1: Observed values (average ± standard deviation, n = number of biological replicates) for the colony diameter, growth curve slope, hydrodynamic radius, and zeta potential data…………………...p. 32

Table S2: Observed values (average ± standard deviation, n = number of biological replicates) for the cyclic-di-GMP and pGpG quantification data in picomoles per 10^9 CFUs……………………………...p. 33

Table S3: Observed values (average ± standard deviation, n = number of biological replicates) for the Congo red and crystal violet data……………………………………………..……........................p. 34

Table S4: Observed values (average ± standard deviation, n = number of biological replicates) for swarming motility, twitching motility, and biofilm initiation…………………………………………………p. 35

Table S5: Observed values (average ± standard deviation, n = number of biological replicates) for the phenazine quantification data…………………………………………………………………………p. 36

Table S6: Observed values (average ± standard deviation, n = number of biological replicates) for the pyochelin and pyoverdine quantification data……………………………………………………..……...p.37

Table S7 - Table of gene mutations shared between RSCV_1 and RSCV_2………………………………………………………………………………………………………p. 38

Table S8 - Table of gene mutations shared between RSCV_1 and RSCV_3………………………………………………………………………………………………………...p. 39

Table S9 - Table of gene mutations shared between RSCV_2 and RSCV_3………………………………………………………………………………………………………...p. 40

Table S10 - Full list of detected mutations in motility related genes in RSCV_1, RSCV_2, or RSCV_3……………………………………………………………………………………………….p. 41

Table S11: Selected locus tag conversions for locus tags discussed in this work………………………………..p. 42-44

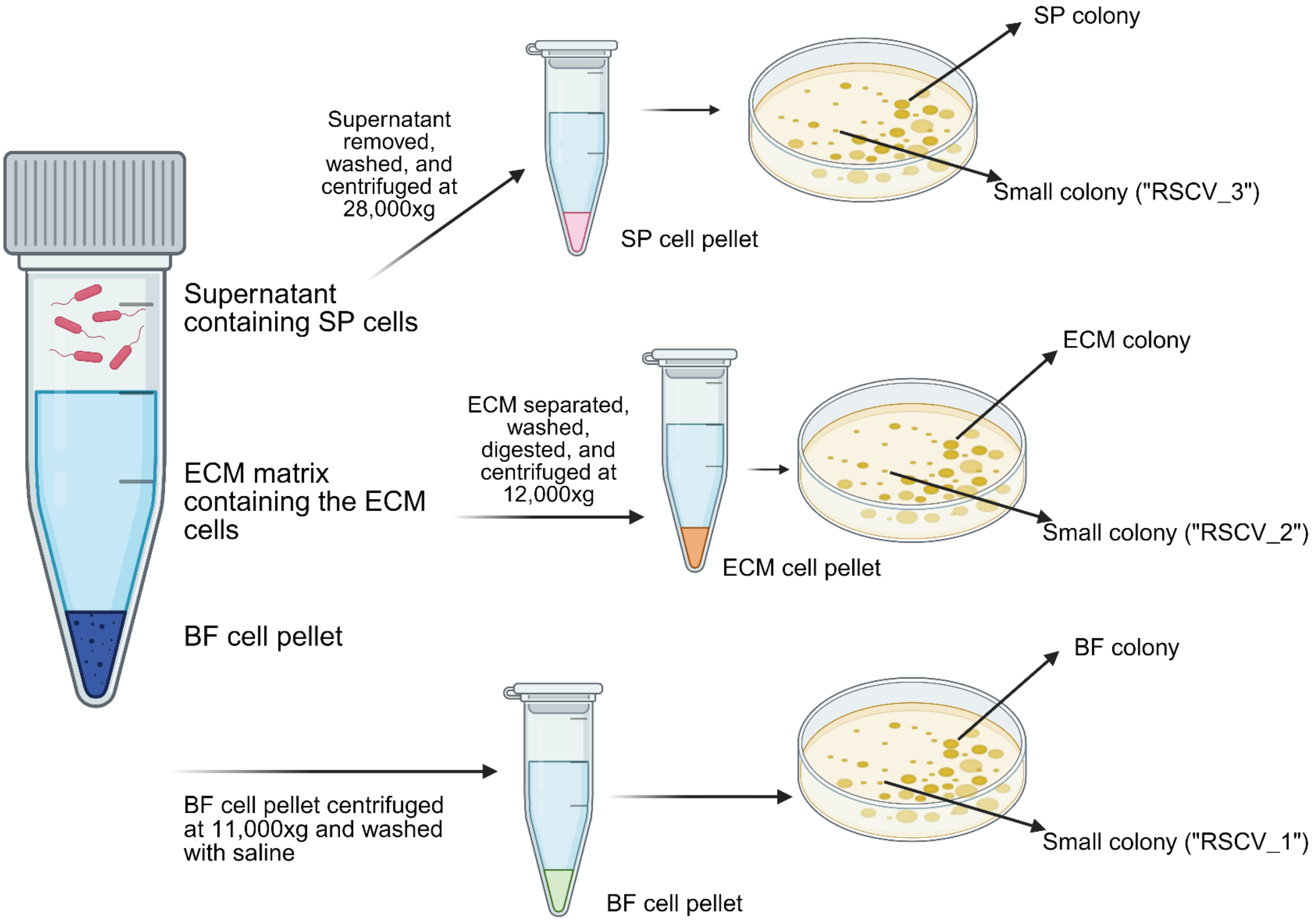

Figure S1: Diagram of the isolation for RSCV_1, RSCV_2, and RSCV_3. Created in BioRender. Smartnick, B. (2025) https://BioRender.com/43rbrcb

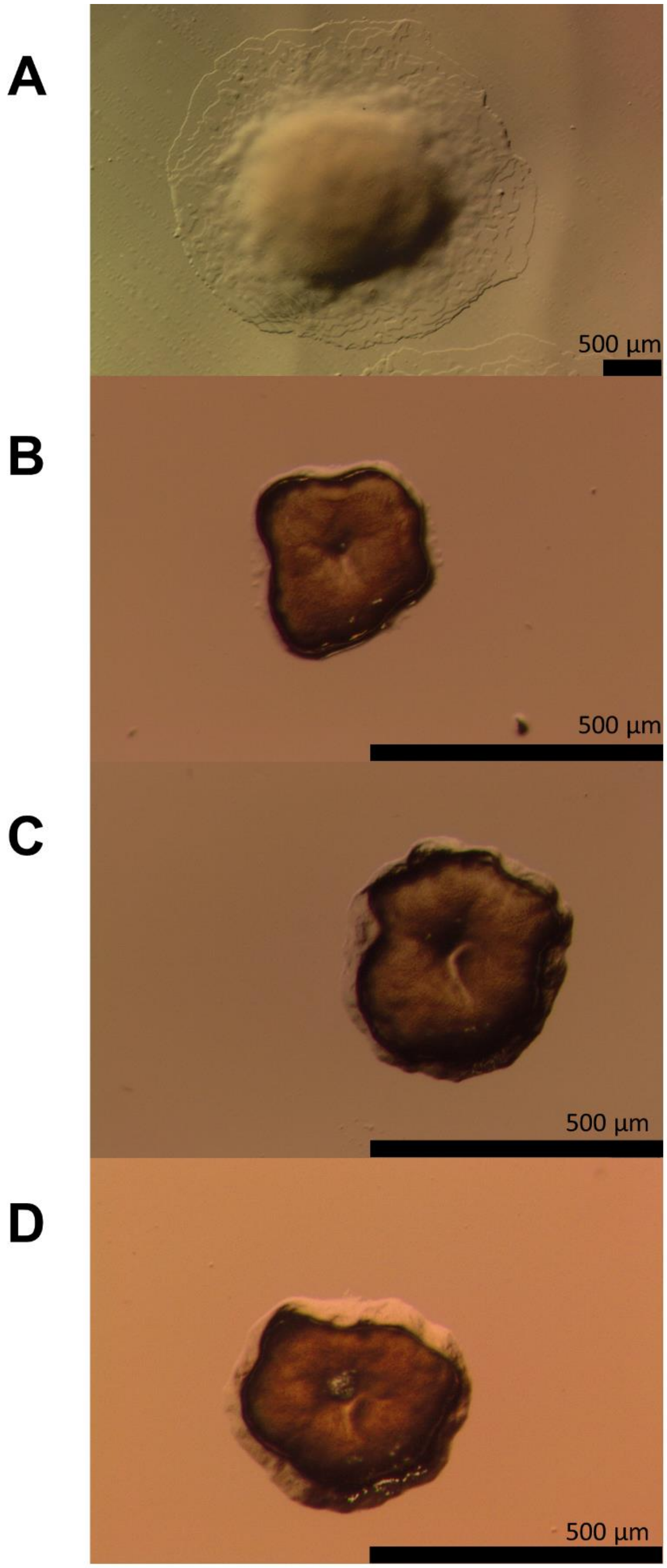

Figure S2: Olympus light microscope images of WT PA14 (A), RSCV_2 (B), RSCV_1 (C), and RSCV_3 (D) colonies.

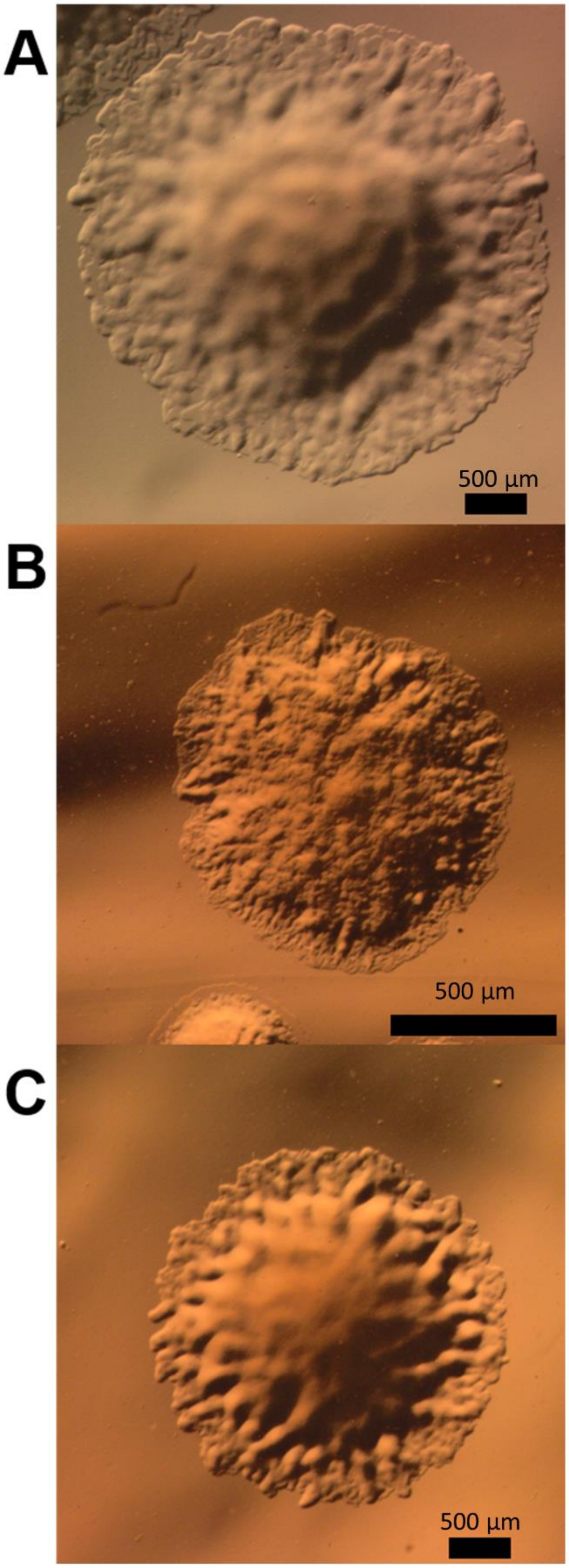

Figure S3: Olympus light microscope images of WT BF (A), WT ECM (B), and WT SP (C) colonies.

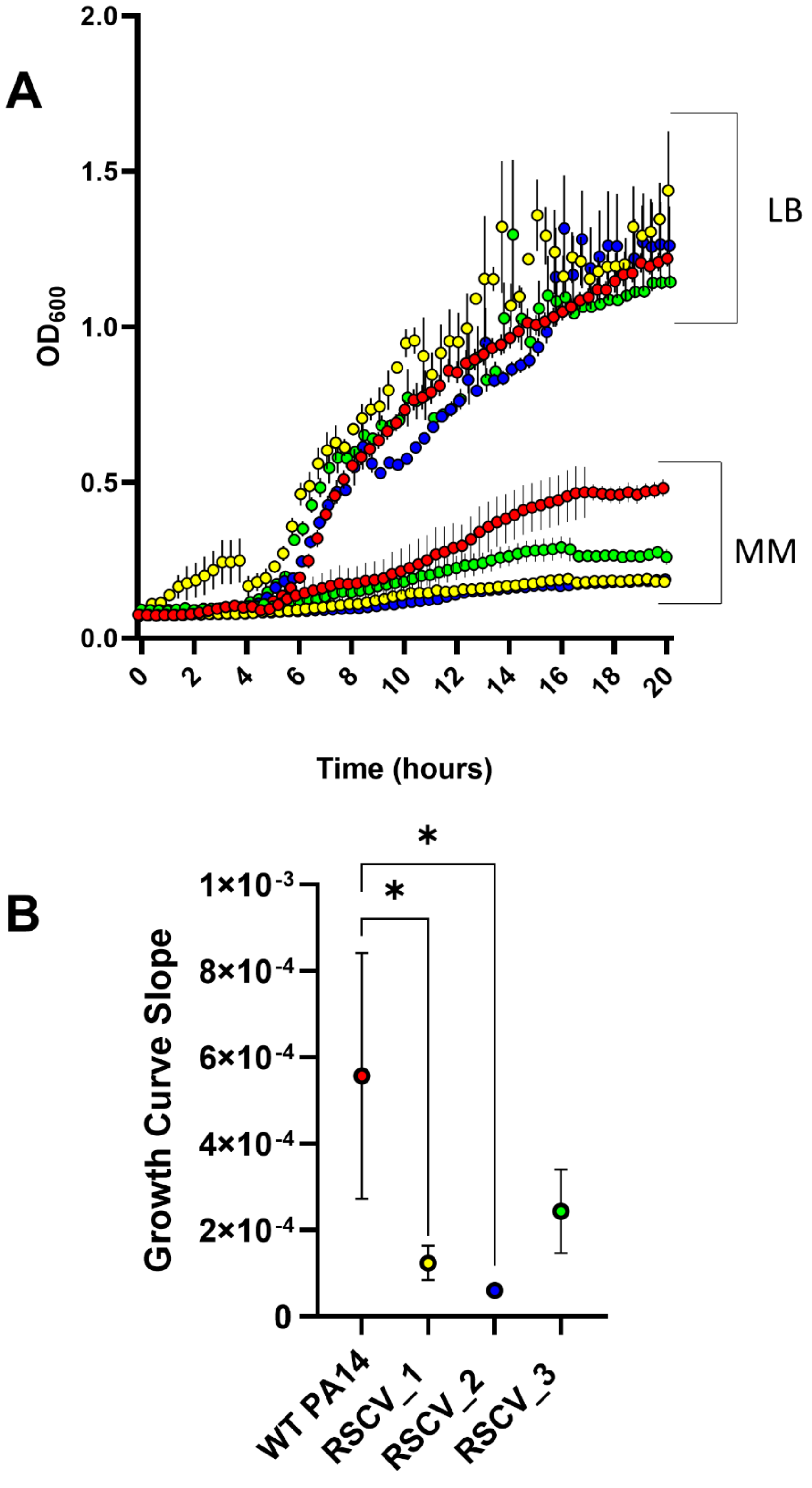

Figure S4: Growth curves in M9 minimal media and LB for RSCV_1, RSCV_2, RSCV_3, and WT PA14. (A) Growth curves in M9 minimal media and LB for WT PA14 (red, n = 4), RSCV_1 (yellow, n = 3), RSCV_2 (blue, n = 3), and RSCV_3 (green, n = 3). Data shown in the LB growth curves is not the same that was used in the growth curve analysis in Figure 1B and was used as a control on the same 96 well-plate. (B) Growth curve slope analysis during M9 minimal media exponential growth phase for RSCV_1 (n = 3), RSCV_2 (n = 3), RSCV_3 (n = 3), and WT PA14 (n = 4). ****, P< 0.0001; ***, P< 0.001, **, P< 0.01, *, P< 0.05.

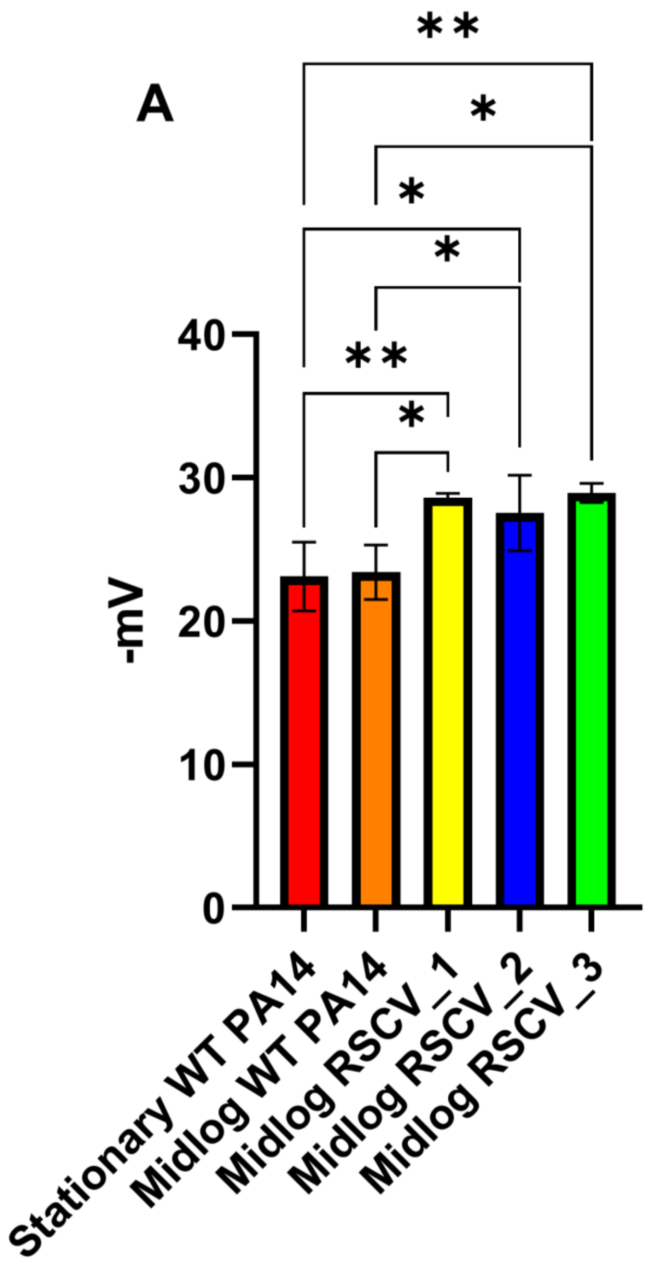

Figure S5: Cell surface charge measurements for cells grown to mid-log phase. (A) Zeta potential measurments for stationary phase WT PA14 (n = 5), mid-log WT PA14 (n = 3), mid-log RSCV_1 (n = 4), mid-log RSCV_2 (n = 5), and mid-log RSCV_3 (n = 5). ****, P< 0.0001; ***, P< 0.001, **, P< 0.01, *, P< 0.05.

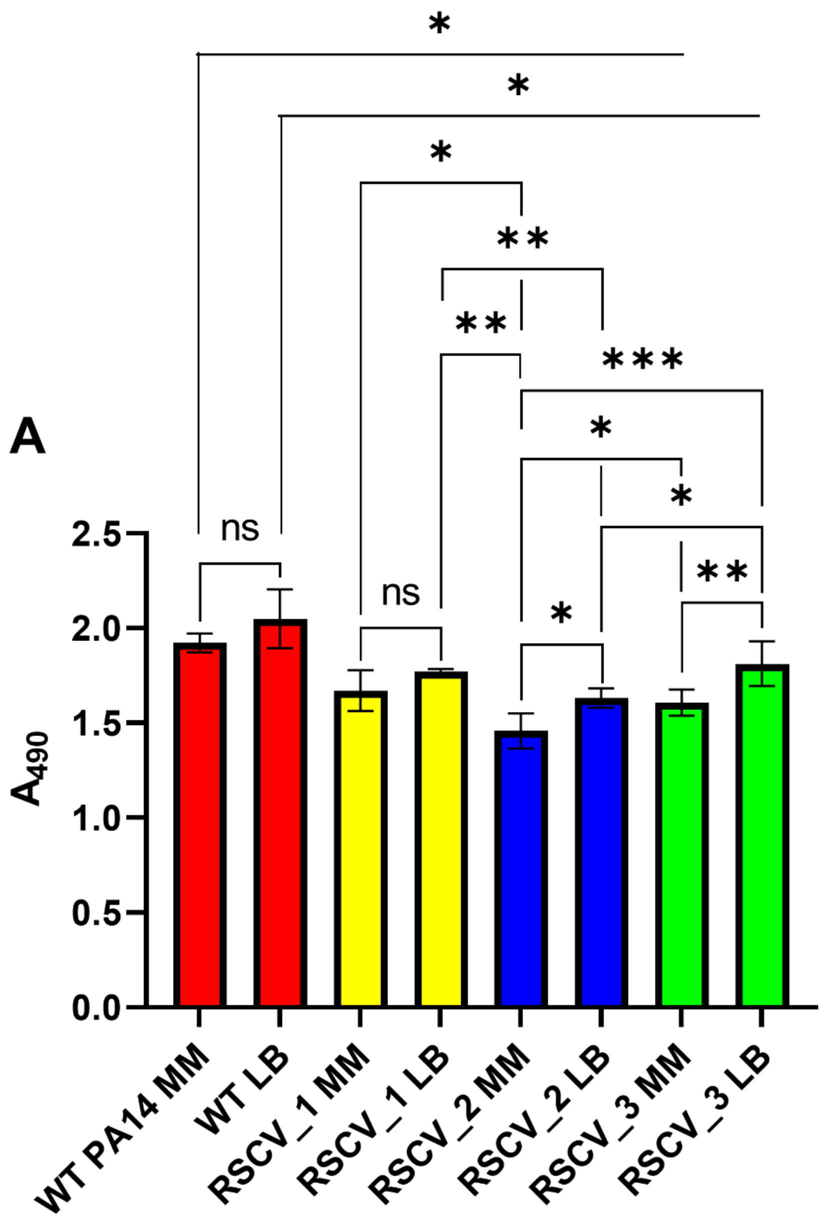

Figure S6: Quantifications of exopolysaccharide production for the RSCVs and WT PA14 in either Luria-Bertani (LB) broth or M9 minimal media (MM). (A) Congo red measurments at an absorbance of 490 nm for WT PA14, RSCV_1, RSCV_2, and RSCV_3 in either LB or M9 minimal media (MM). Number of biological replicates per sample between 3 and 6. LB Congo red data is the same as is shown in Figure 3A. ****, P< 0.0001; ***, P< 0.001, **, P< 0.01, *, P< 0.05.

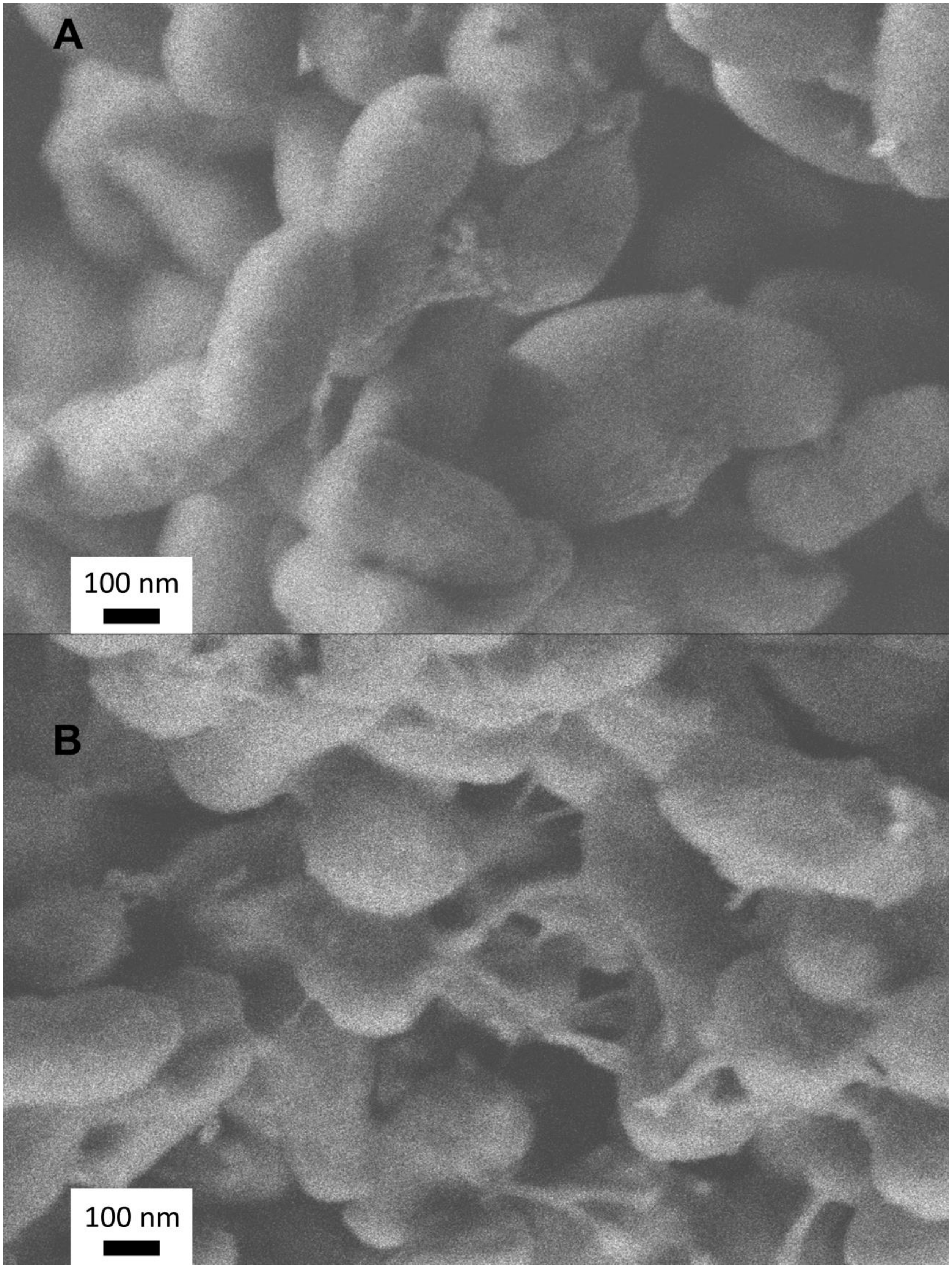
A
100 nm
B
100 nm

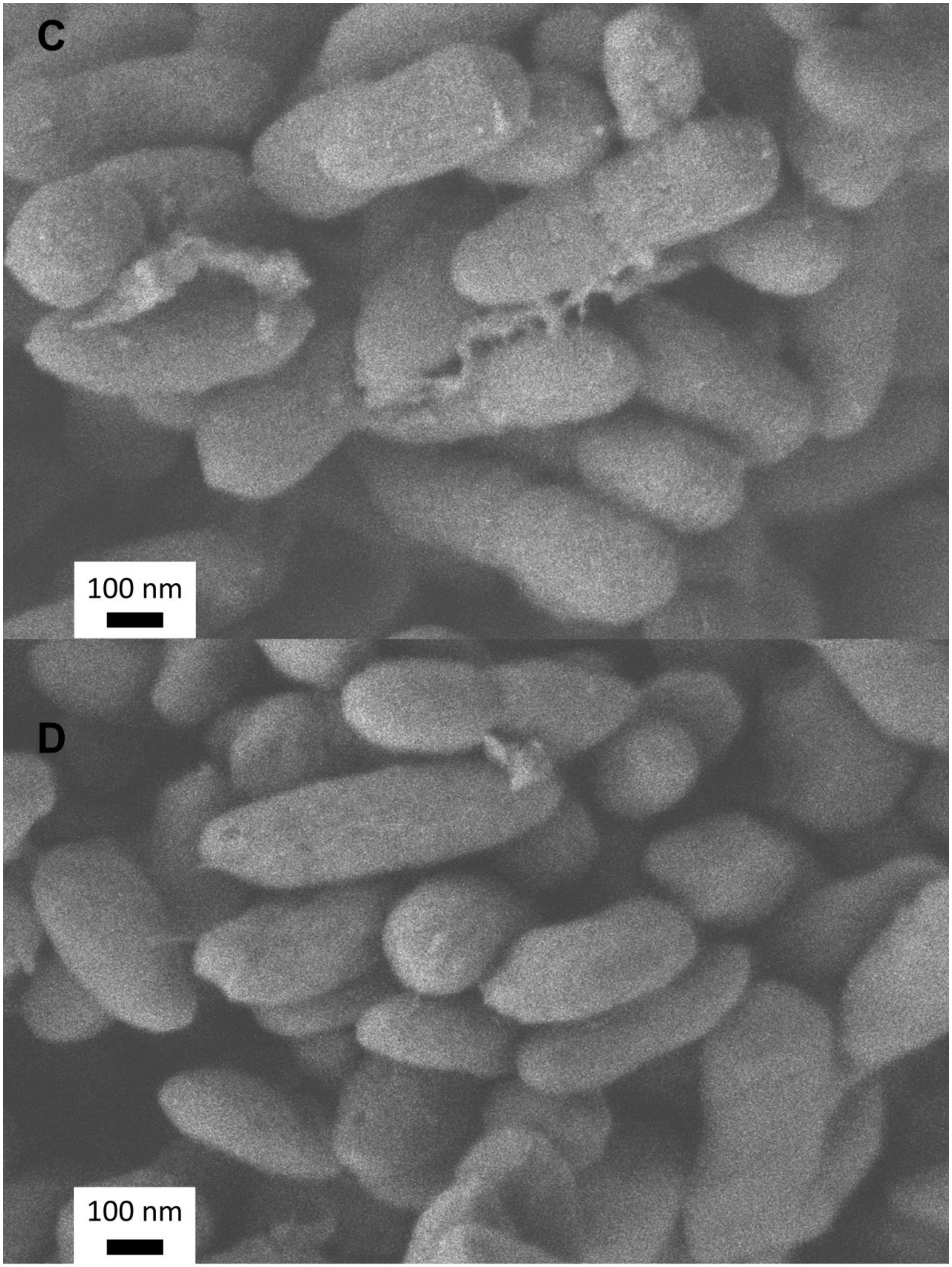

Figure S7: SEM images of RSCV_1 (A), RSCV_2 (B), RSCV_3 (C), and stationary WT PA14 (D).

A

	RSCV_1 vs Stationary WT	RSCV_2 vs Stationary WT	RSCV_3 vs Stationary WT	RSCV_1 vs RSCV_2	RSCV_1 vs RSCV_3	RSCV_2 vs RSCV_3
0.2 µg/mL	<0.0001	0.00220	<0.0001	0.531	0.999	0.401
0.4 µg/mL	0.00120	0.0331	<0.0001	0.702	0.138	0.00860
0.6 µg/mL	0.290	0.999	<0.0001	0.227	0.056	<0.0001
0.8 µg/mL	0.438	0.997	0.00810	0.570	0.330	0.0157
1.0 µg/mL	0.620	0.946	0.161	0.911	0.838	0.426

B

	RSCV_1 vs Stationary WT	RSCV_2 vs Stationary WT	RSCV_3 vs Stationary WT	RSCV_1 vs RSCV_2	RSCV_1 vs RSCV_3	RSCV_2 vs RSCV_3
1.0 µM	0.692	0.965	0.259	0.949	0.914	0.633
1.5 µM	<0.0001	0.00210	<0.0001	<0.0001	0.0231	0.353
2.0 µM	0.093	0.625	0.0199	0.746	0.949	0.415
2.5 µM	0.068	0.214	0.0193	0.964	0.973	0.802
3.0 µM	0.129	0.337	0.0281	0.968	0.944	0.740

Figure S8: Statistical analysis of the accumulation of biomass in the presence of colistin and tobramycin. (A) Table of all p-values for the colistin antibiotic crystal violet accumulation assay (n = 5 or 6 for each sample's colistin concentration). (B) Table of all p-values for the tobramycin antibiotic biofilm crystal violet accumulation assay (n = 3 or 6 for each sample's tobramycin concentration). Two-way ANOVA with Tukey's multiple comparisons was used to calculate all p-values shown in the antibiotic tolerance assays.

A

	RSCV_1 vs Stationary WT	RSCV_2 vs Stationary WT	RSCV_3 vs Stationary WT	RSCV_1 vs RSCV_2	RSCV_1 vs RSCV_3	RSCV_2 vs RSCV_3
30 µg/mL	<0.0001	<0.0001	<0.0001	0.991	0.0272	0.0118
50 µg/mL	<0.0001	<0.0001	<0.0001	0.540	0.796	0.975
70 µg/mL	0.00100	<0.0001	0.00110	0.366	1.000	0.342
100 µg/mL	0.0383	0.00320	0.536	0.770	0.600	0.156
150 µg/mL	0.0315	0.0253	0.00460	0.997	0.903	0.971

B

	RSCV_1 vs Stationary WT	RSCV_2 vs Stationary WT	RSCV_3 vs Stationary WT	RSCV_1 vs RSCV_2	RSCV_1 vs RSCV_3	RSCV_2 vs RSCV_3
20 µM	3.00e-4	<0.0001	<0.0001	0.00670	0.057	0.884
40 µM	0.00330	<0.0001	<0.0001	0.00920	0.082	0.863
60 µM	0.463	<0.0001	0.00580	<0.0001	0.256	0.0121
100 µM	0.133	<0.0001	0.0421	0.00120	0.928	0.0152
150 µM	0.052	<0.0001	0.0182	0.149	0.979	0.306

Figure S9: Statistical analysis of the dispersion of biofilms with colistin and tobramcyin. (A) Table of all p-values for the colistin antibiotic crystal violet dissociation assay (n = 5 or 6 for each sample's tobramycin concentration). (B) Table of all p-values for the tobramycin antibiotic biofilm crystal violet dissociation assay (n = 5 or 6 for each sample's colistin concentration). Two-way ANOVA with Tukey's multiple comparisons was used to calculate all p-values shown in the antibiotic tolerance assays.

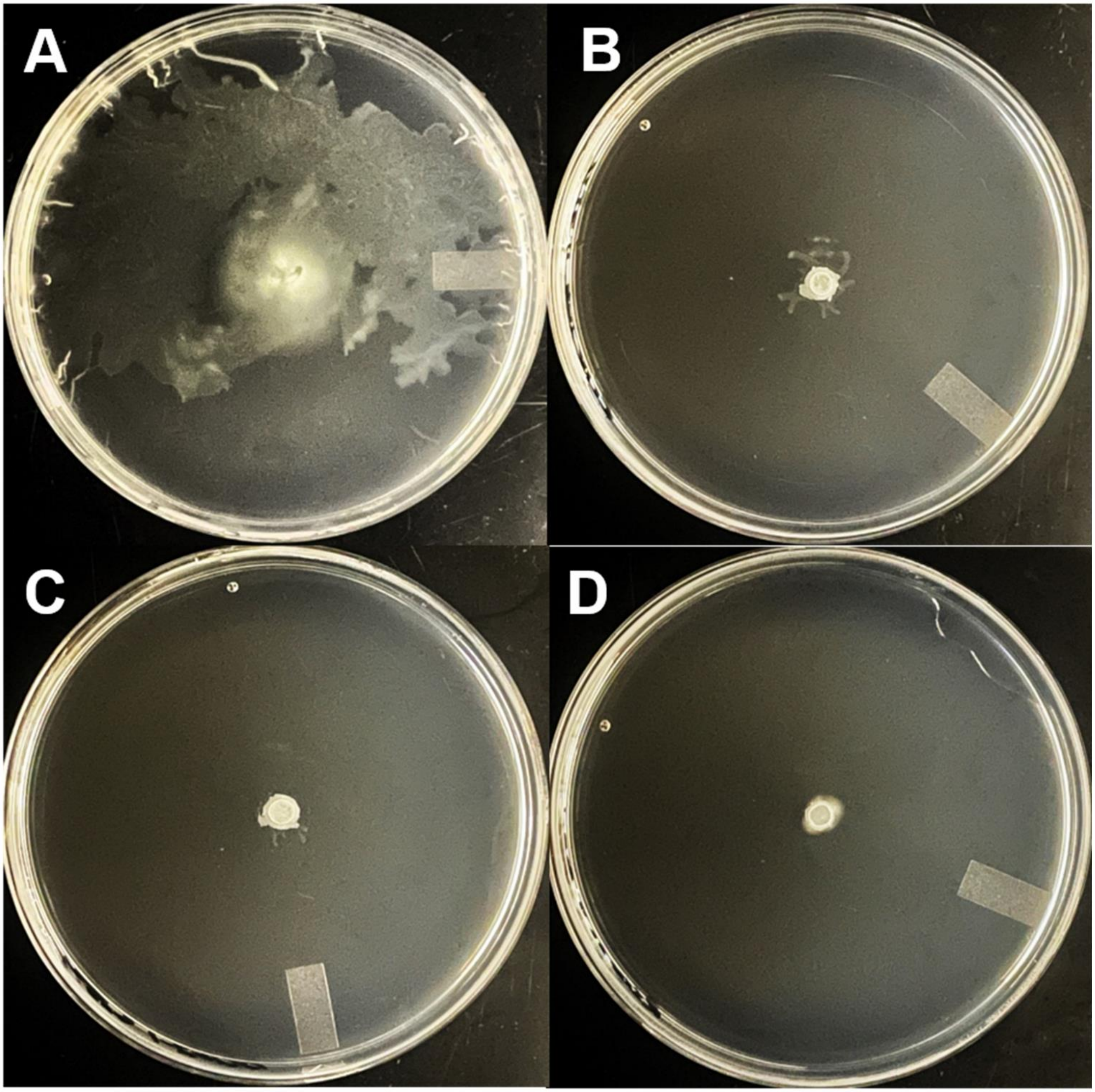

Figure S10: Representative swarming images of stationary WT PA14 (A), RSCV_1 (B), RSCV_2 (C), and RSCV_3 (D) with 0.25% agar.

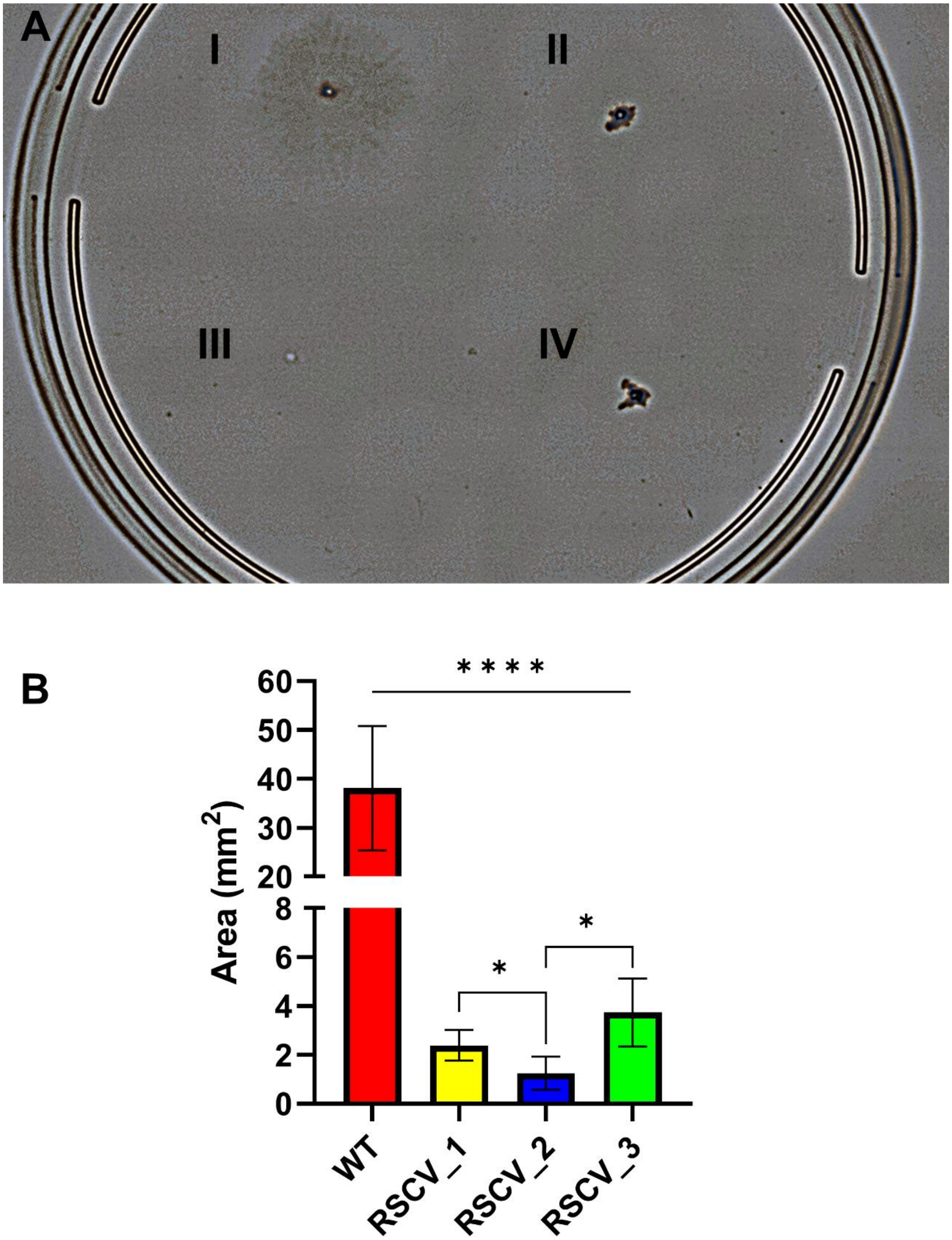

Figure S11: Additional twitching data for RSCV_1, RSCV_2, RSCV_3, and stationary WT PA14. (A) Representative twitching image of stationary WT PA14 (I), RSCV_1 (II), RSCV_2 (III), and RSCV_3 (IV). (B) Quantification of the twitching areas for stationary WT (n = 5), RSCV_1 (n = 5), RSCV_2 (n = 4), and RSCV_3 (n = 4). Welch's Unpaired two-tailed t-test was used for statistical analysis. ****, P< 0.0001; ***, P< 0.001, **, P< 0.01, *, P< 0.05.

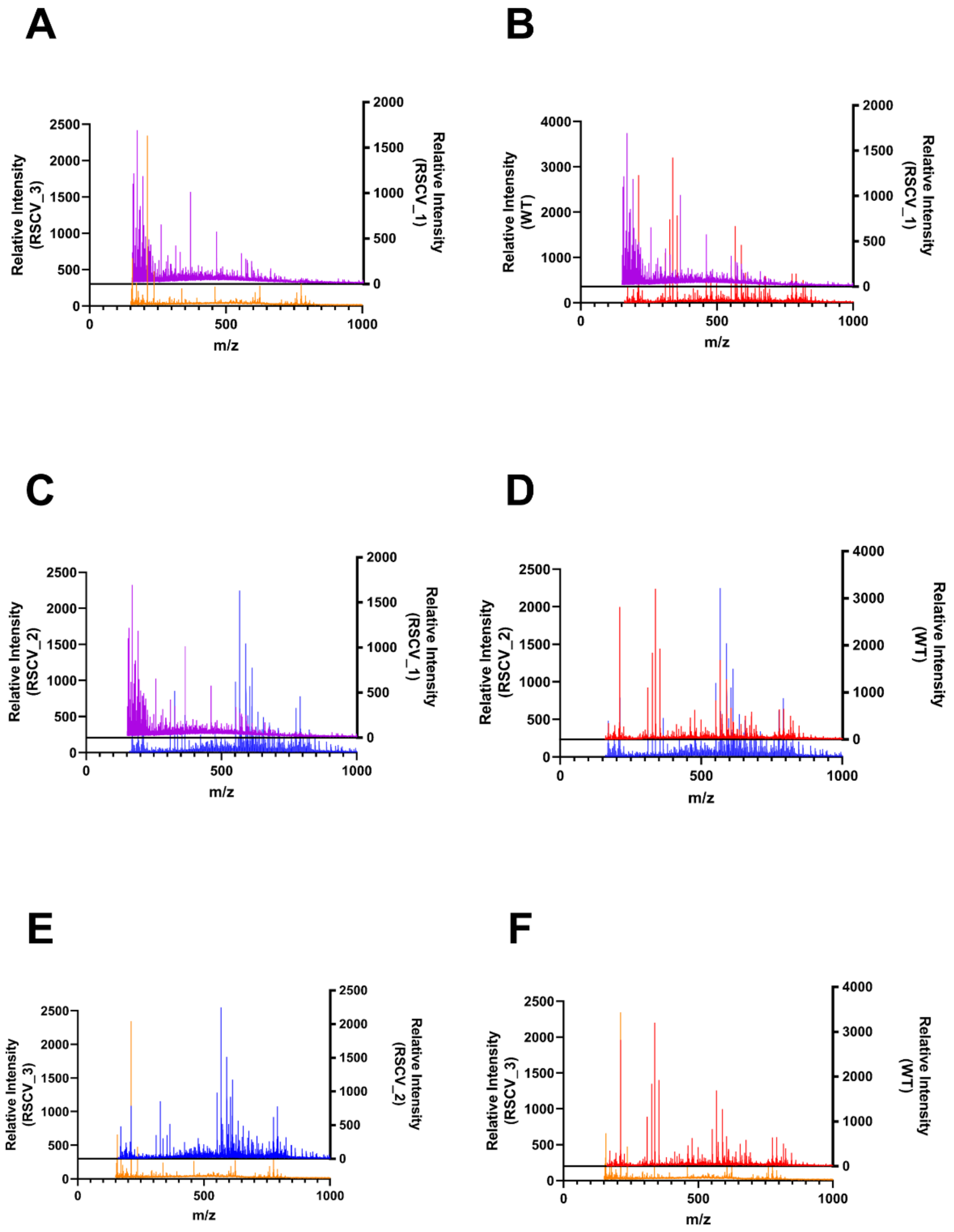

Figure S12: Overlaid MALDI reflectron positive ionization mode scans mass spectra of the three RSCVs and stationary WT PA14. (A) Reflectron positive mode overlaid spectra of RSCV_1 (purple) and RSCV_3 (yellow). (B) Reflectron positive mode overlaid spectra of RSCV_1 (purple) and stationary WT PA14 cells (red). (C) Reflectron positive mode overlaid spectra of RSCV_2 (blue) and RSCV_1 (purple). (D) Reflectron positive mode overlaid spectra of RSCV_2 (blue) and stationary WT PA14 cells (red). (E) Reflectron positive mode overlaid spectra of RSCV_3 (yellow) and RSCV_2 (blue). (F) Reflectron positive mode overlaid spectra of RSCV_3 (yellow) and stationary WT PA14 cells (red).

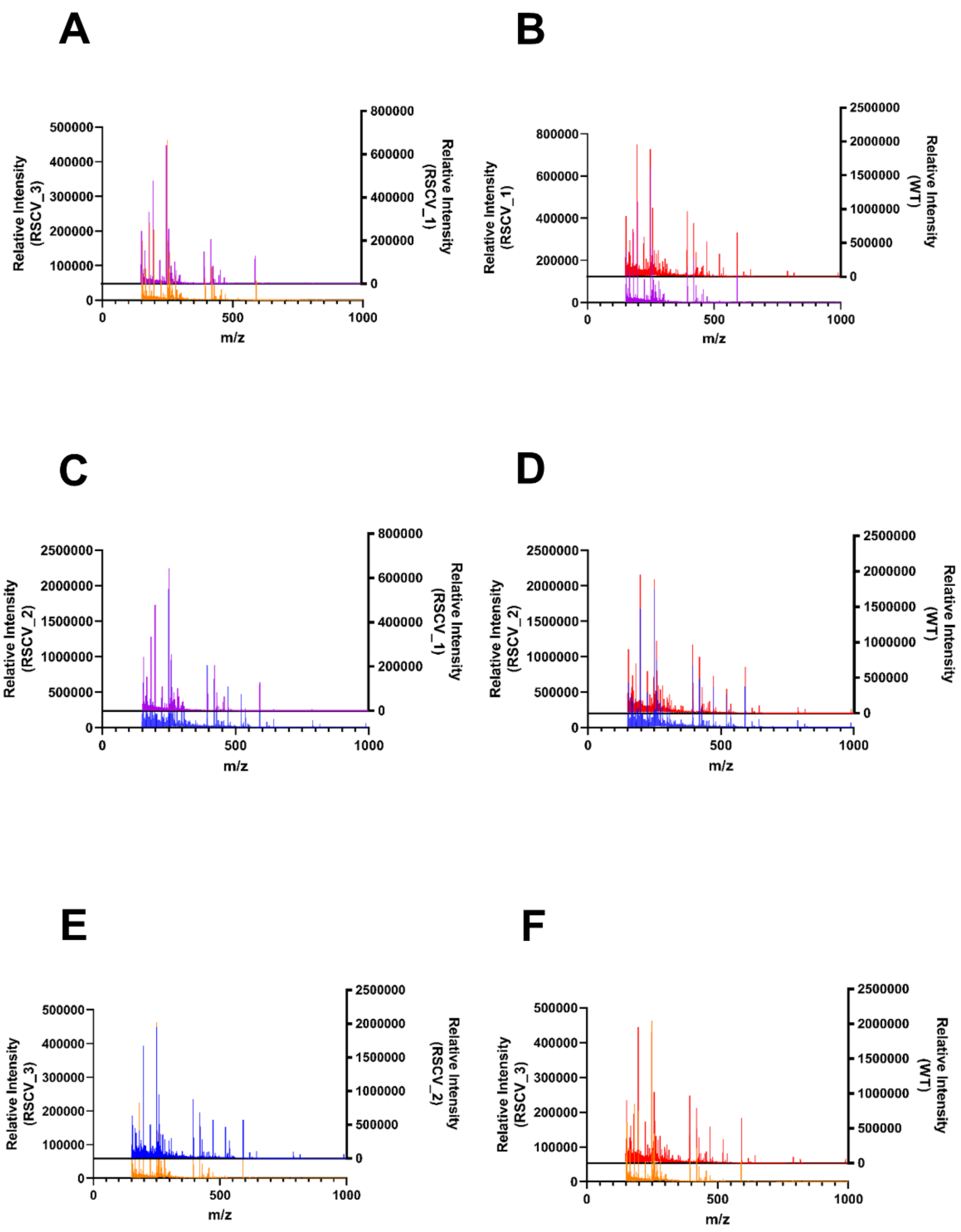

Figure S13: Overlaid MALDI reflectron negative ionization mode scans mass spectra of the RSCVs and WT PA14 cells. (A) Reflectron negative overlaid spectra of RSCV_1 (purple) and RSCV_3 (yellow). (B) Reflectron negative overlaid spectra of RSCV_1 (purple) and RSCV_2 (blue). (C) Reflectron negative overlaid spectra of RSCV_1 (purple) and RSCV_2 (blue). (D) Reflectron negative overlaid spectra of WT stationary PA14 cells (red) and RSCV_2 (blue). (E) Reflectron negative overlaid spectra of RSCV_2 (blue) and RSCV_3 (yellow). (F) Reflectron negative overlaid spectra of RSCV_3 (yellow) and WT stationary PA14 cells (red).

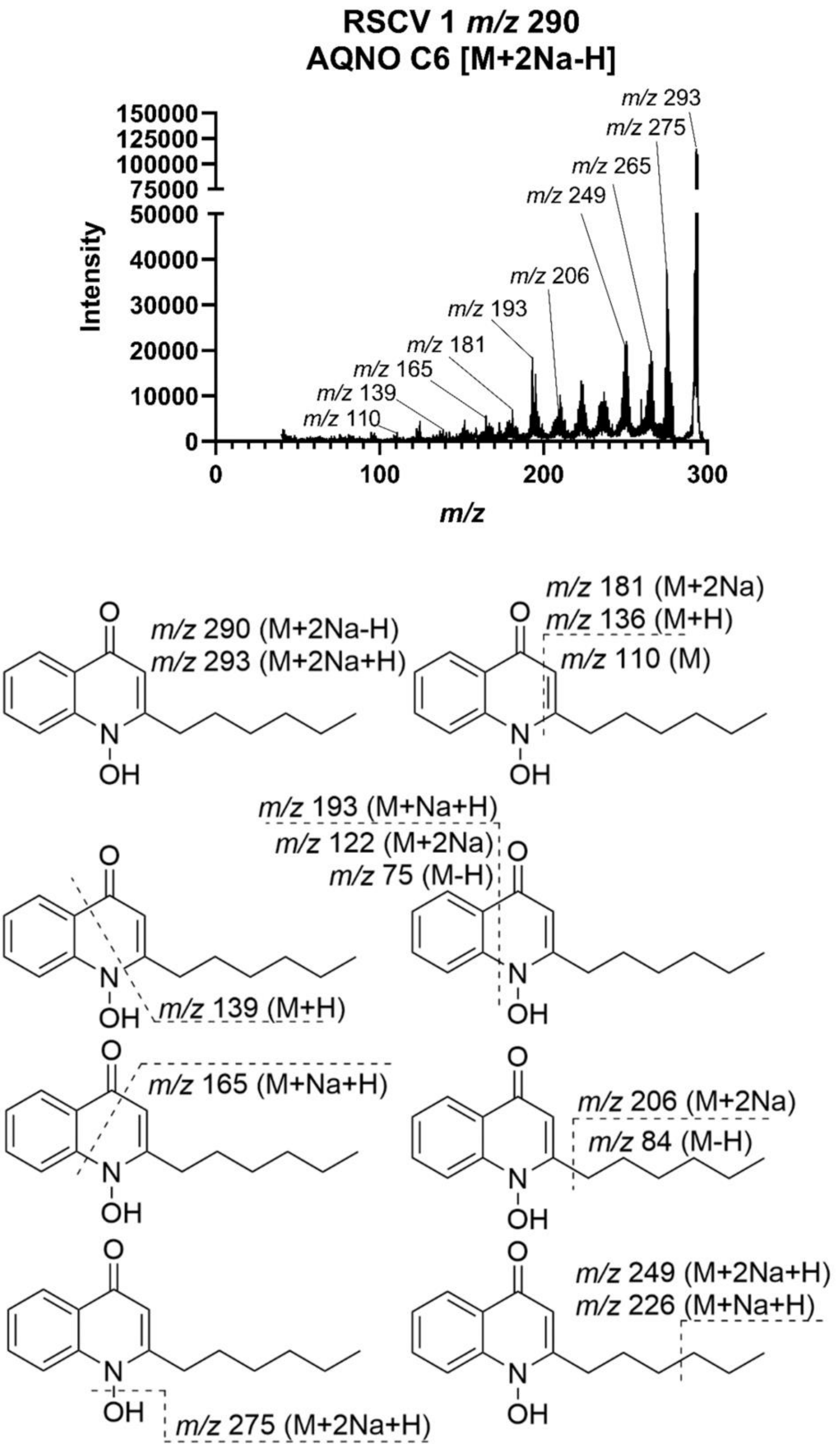

Figure S14: CID positive ionization mode fragmentation of 290 *m/z* in RSCV_1.

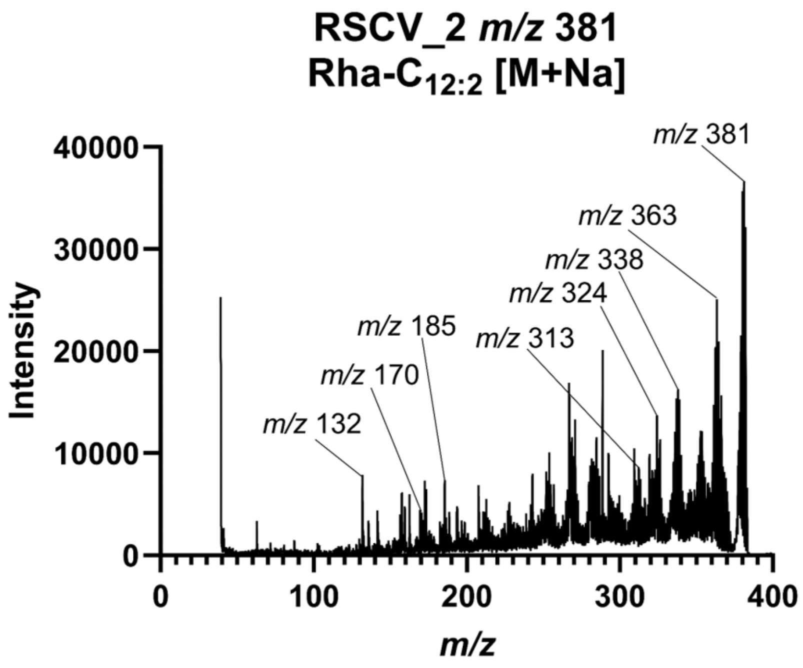

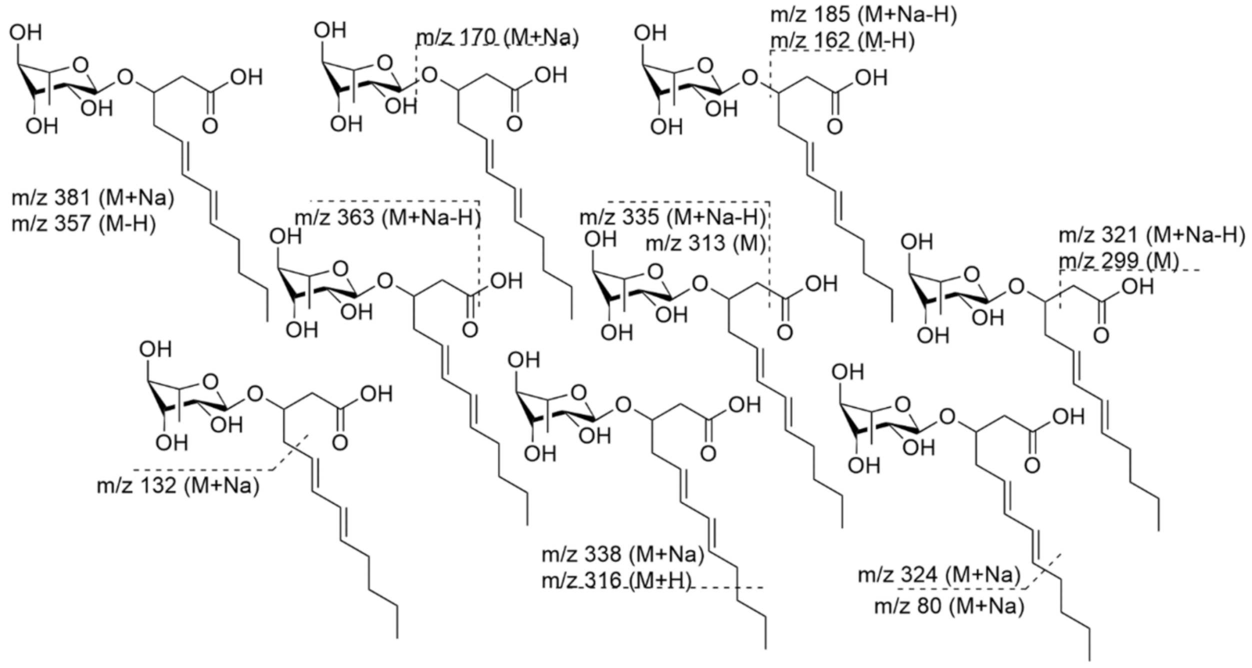

Figure S15: CID positive ionization mode fragmentation of 381 *m/z* in RSCV_2.

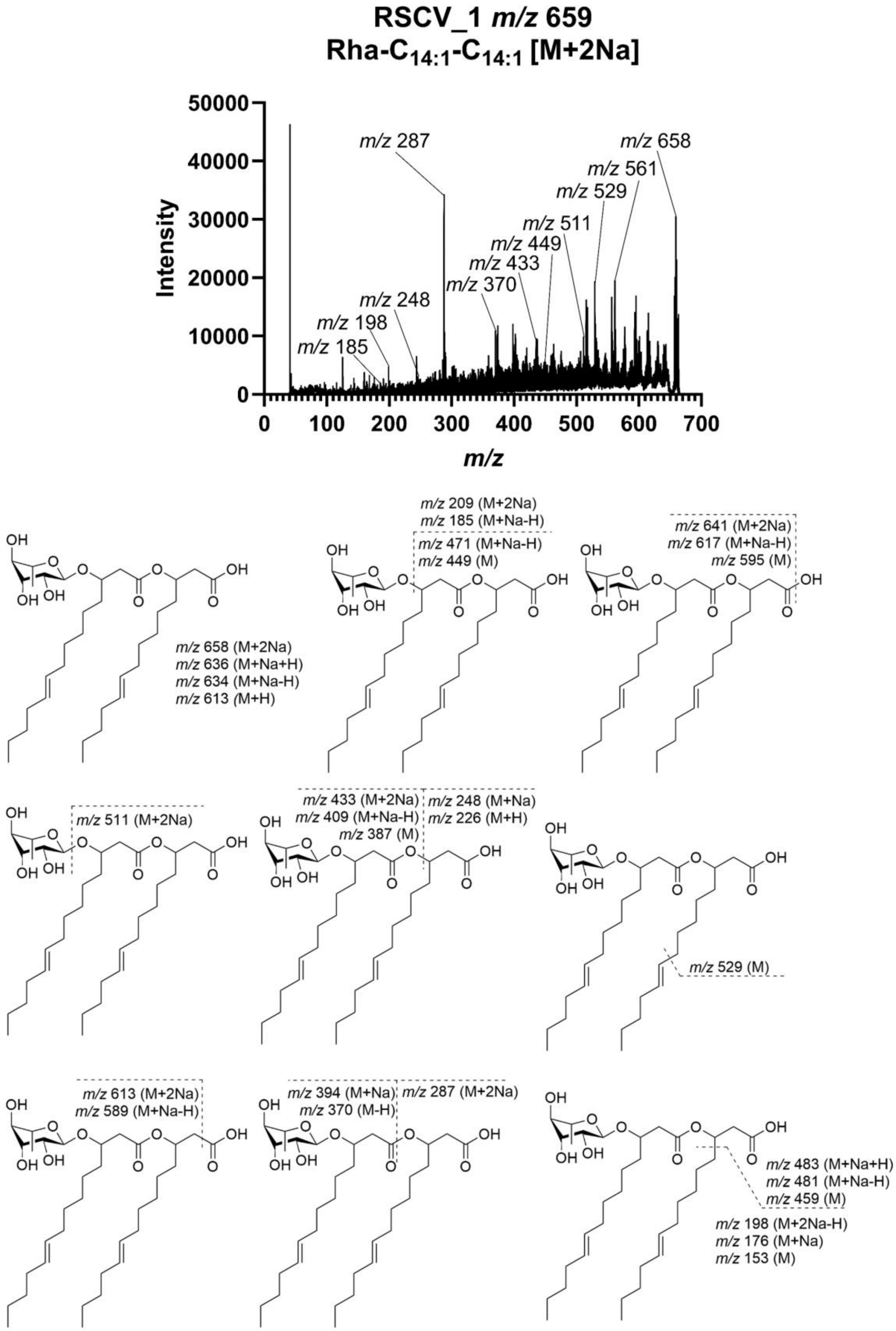

Figure S16: CID positive ionization mode fragmentation of 659 *m/z* in RSCV_1.

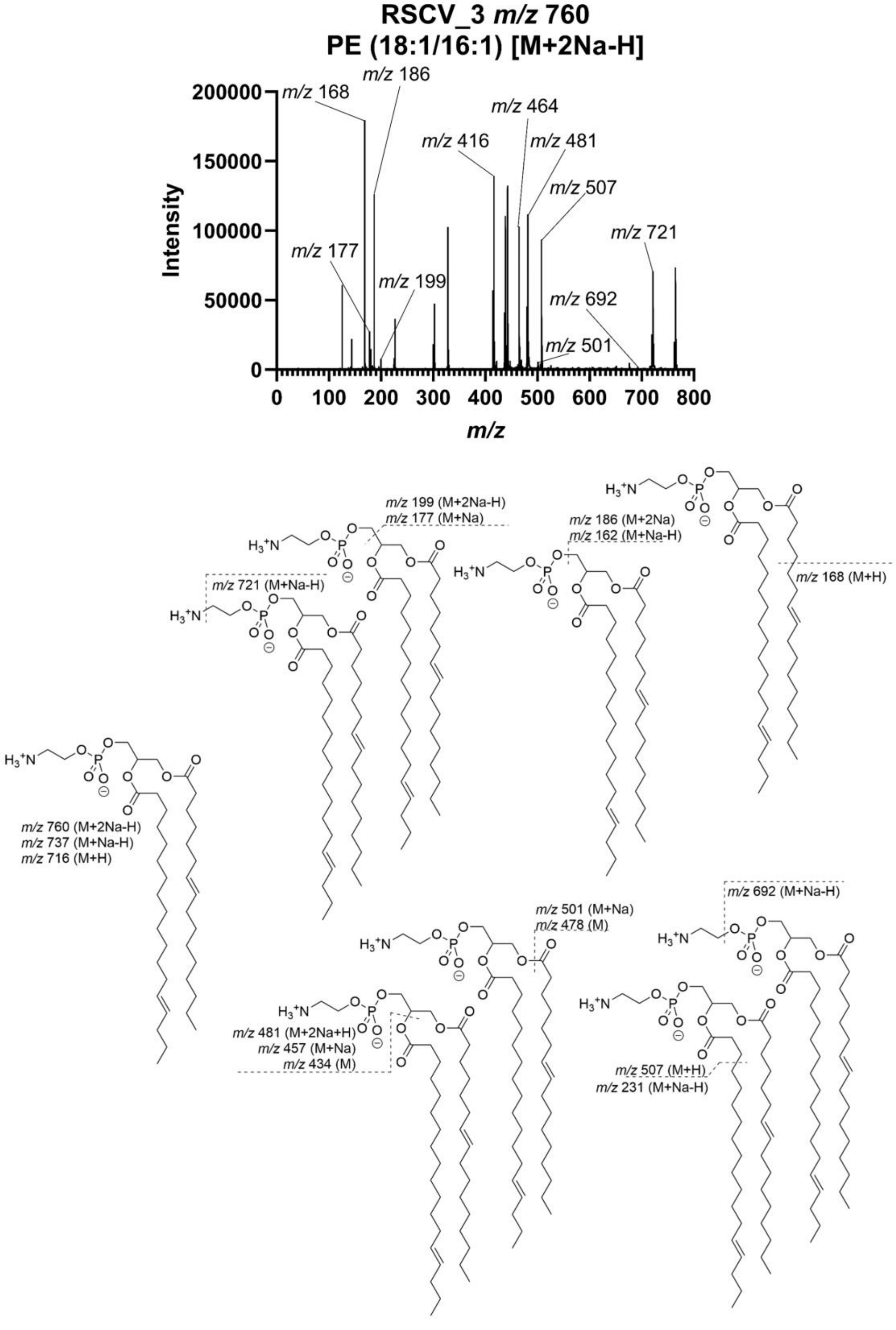

Figure S17: CID positive ionization mode fragmentation of 760 *m/z* in RSCV_3.

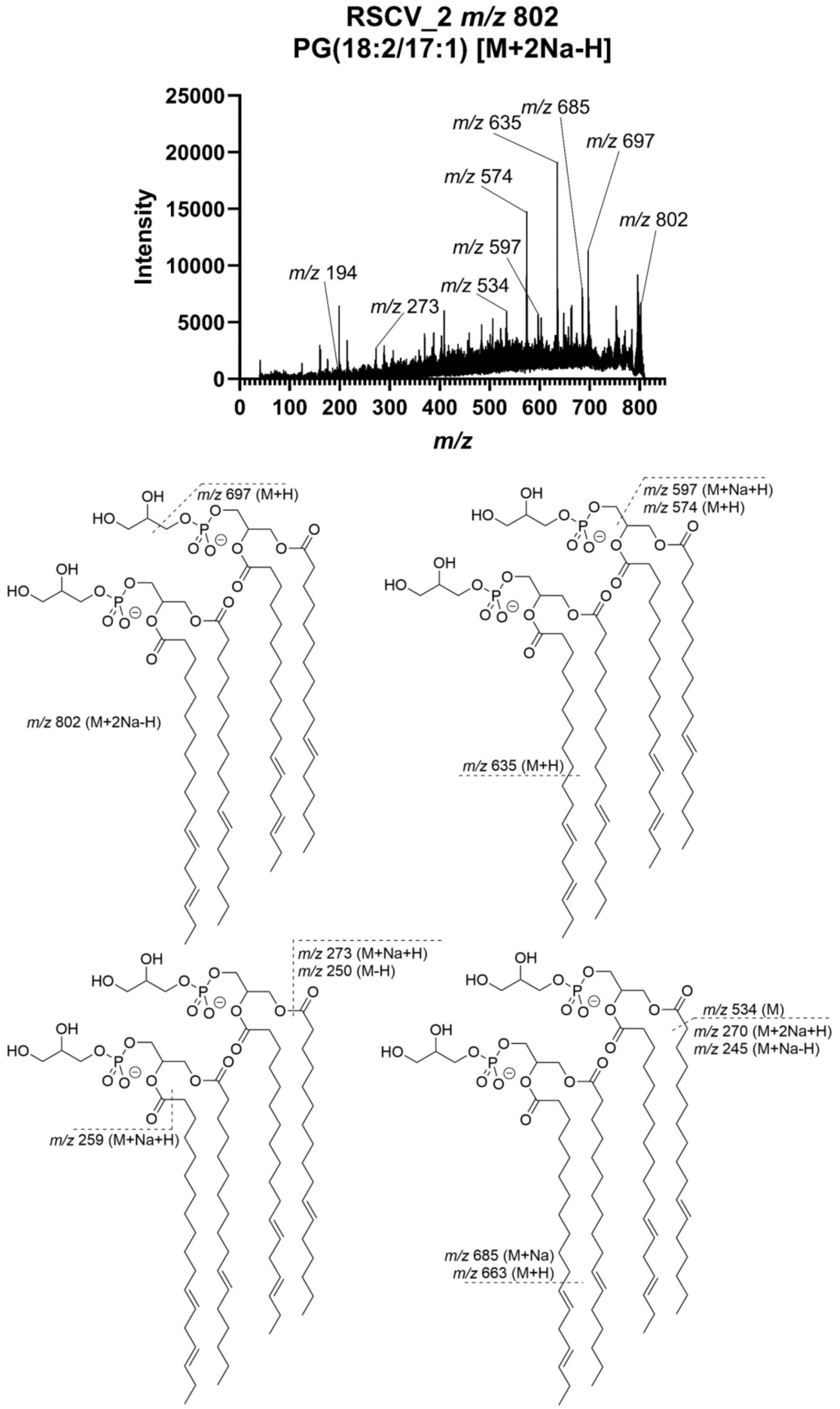

Figure S18: CID positive ionization mode fragmentation of 802 *m/z* in RSCV_2.

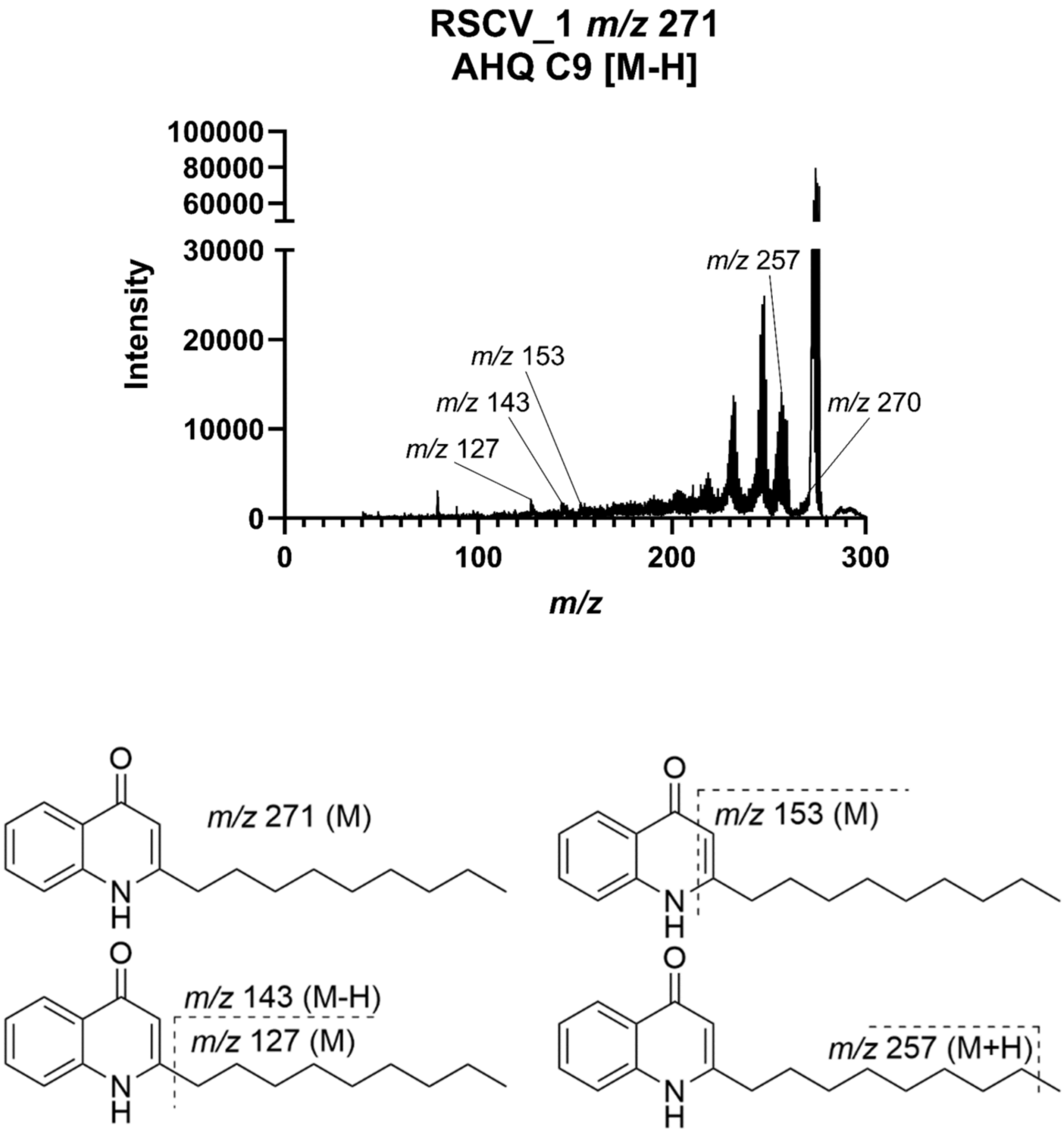

Figure S19: CID negative ionization mode fragmentation of 271 *m/z* for RSCV_1.

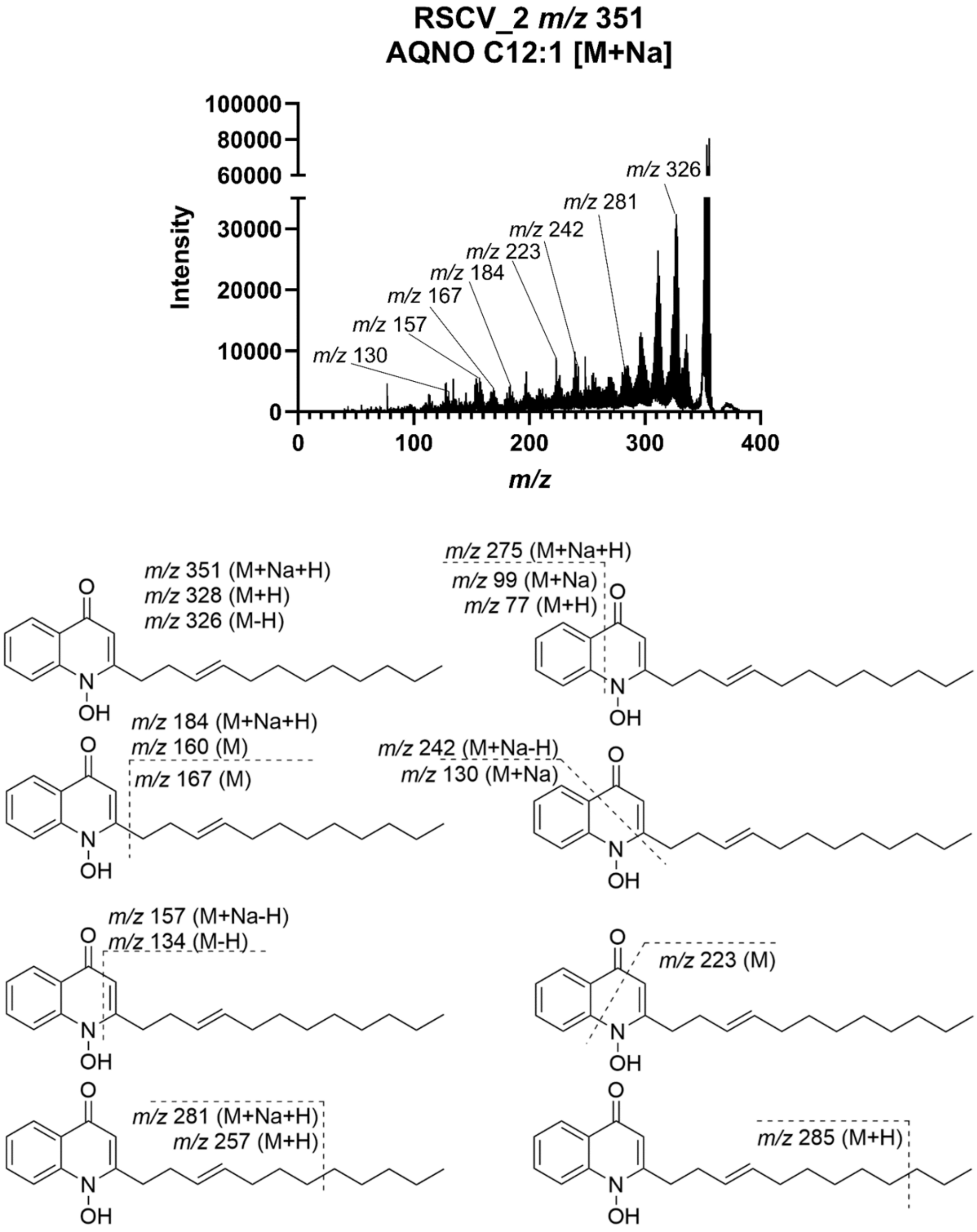

Figure S20: CID negative ionization mode fragmentation of 351 *m/z* for RSCV_2.

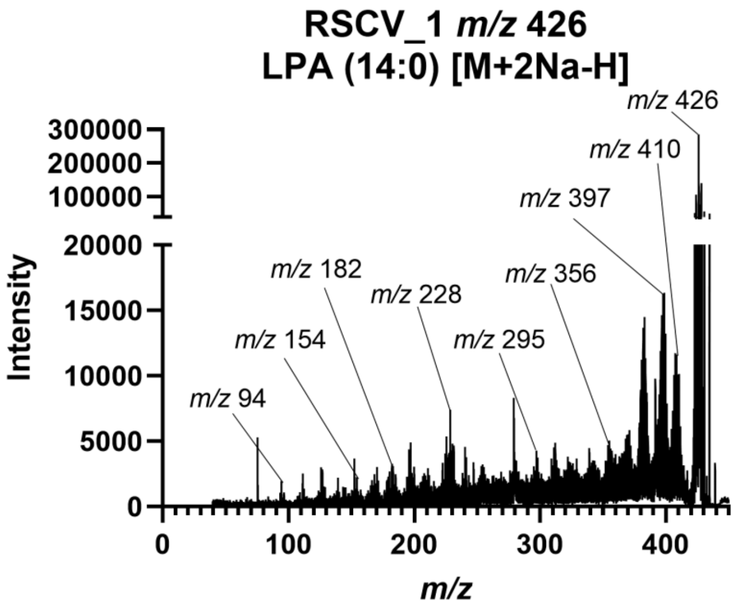

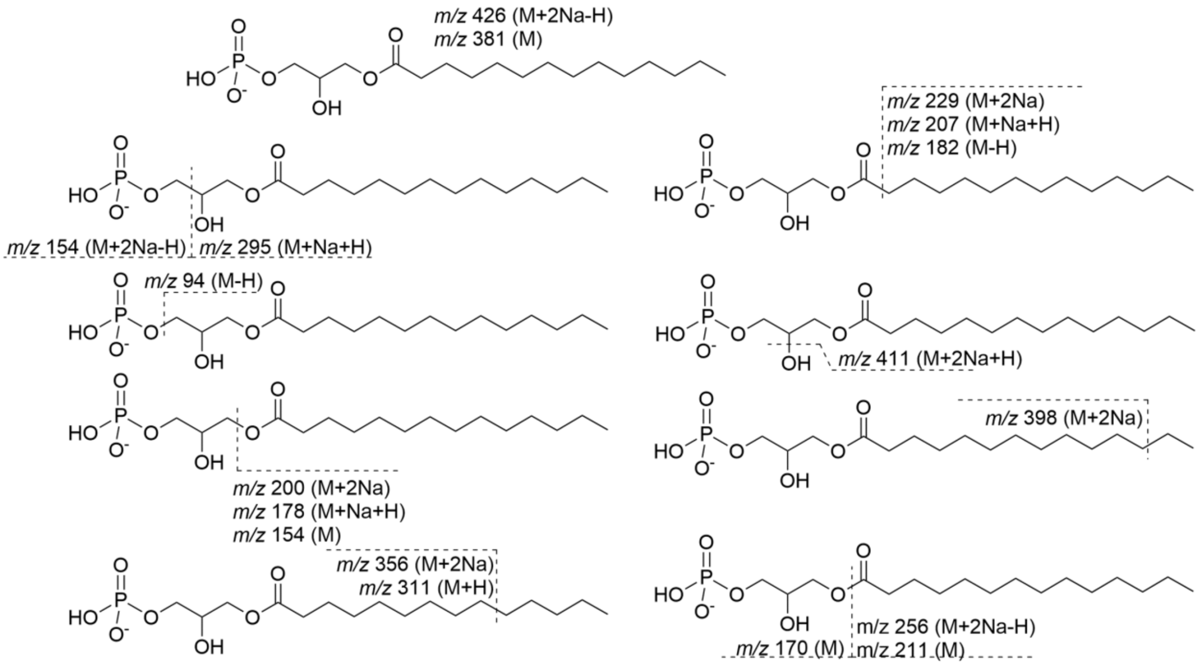

Figure S21: CID negative ionization mode fragmentation of 426 *m/z* for RSCV_1.

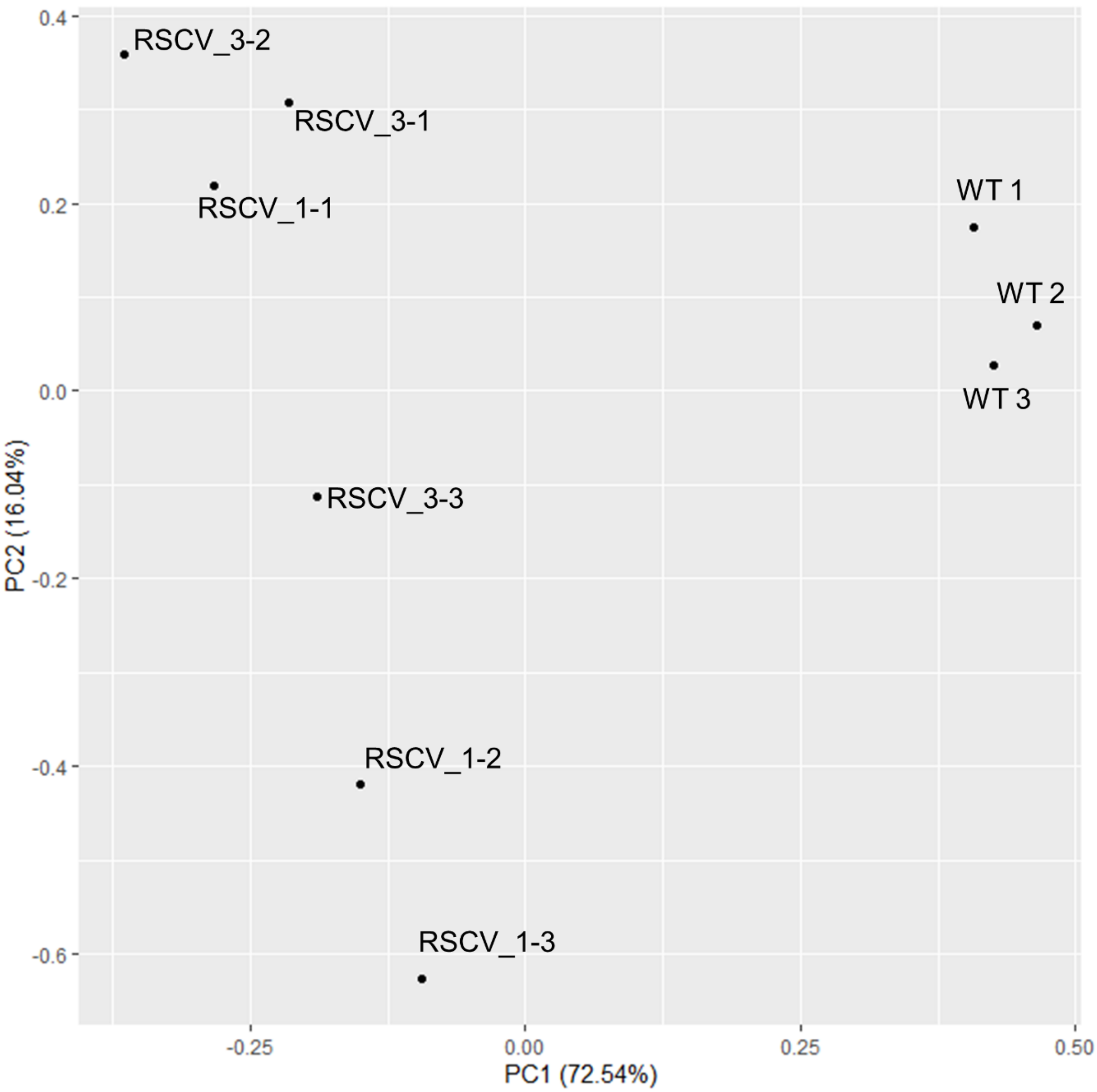

Figure S22: Fuzzy Clustering Analysis (FCA) of the RNA-seq normalized raw counts for RSCV_1, RSCV_3, and stationary WT PA14.

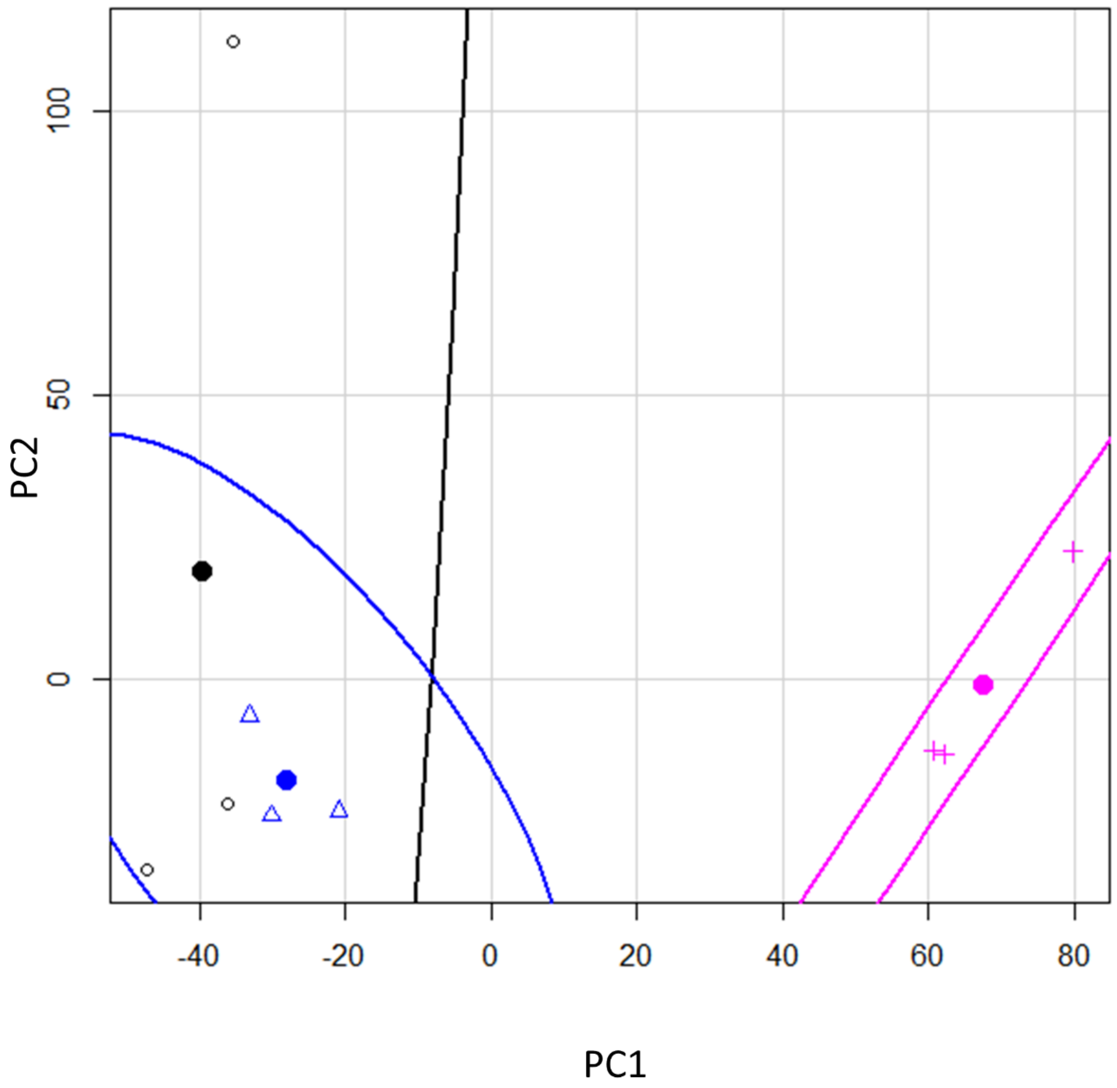

Figure S23: Principal component analysis of the clustered RNA-seq normalized raw counts for RSCV_1 (blue), RSCV_3 (black), and WT PA14 (purple). Lines represent the calculated 95% confidence interval for each cluster.

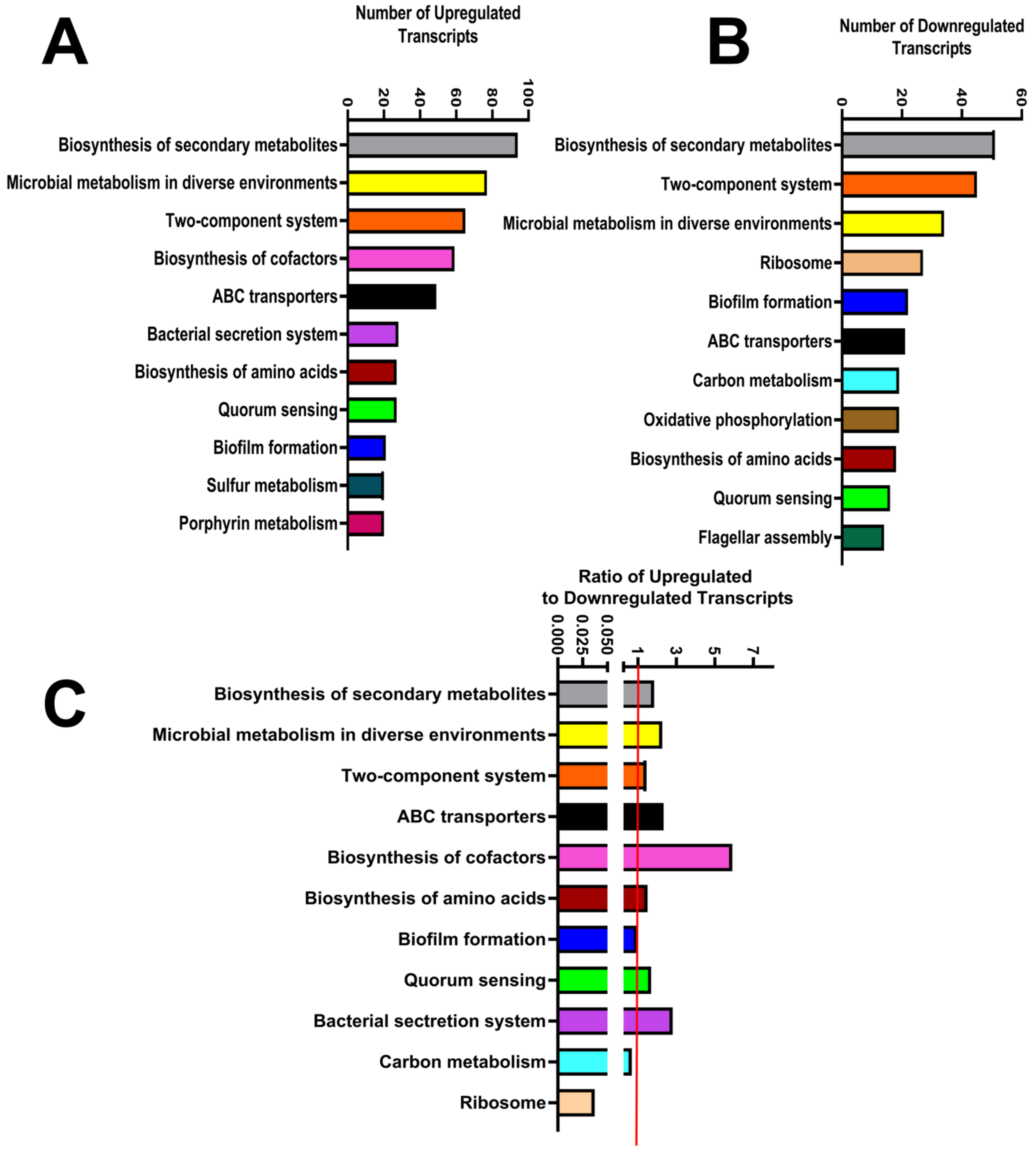

Figure S24: KEGG (Kyoto Encyclopedia of Genes and Genomes) pathway analysis for stationary WT PA14 compared to RSCV_1 and RSCV_3. (A) The eleven KEGG pathways with the highest number of upregulated transcripts between WT PA14 and both of the RSCVs. (B) The eleven KEGG pathways with the highest number of downregulated transcripts between WT PA14 and both of the RSCVs. (C) The ratio of upregulated to downregulated transcripts for the eleven KEGG pathways with the highest number of differentially expressed genes between WT PA14 and both of the RSCVs.

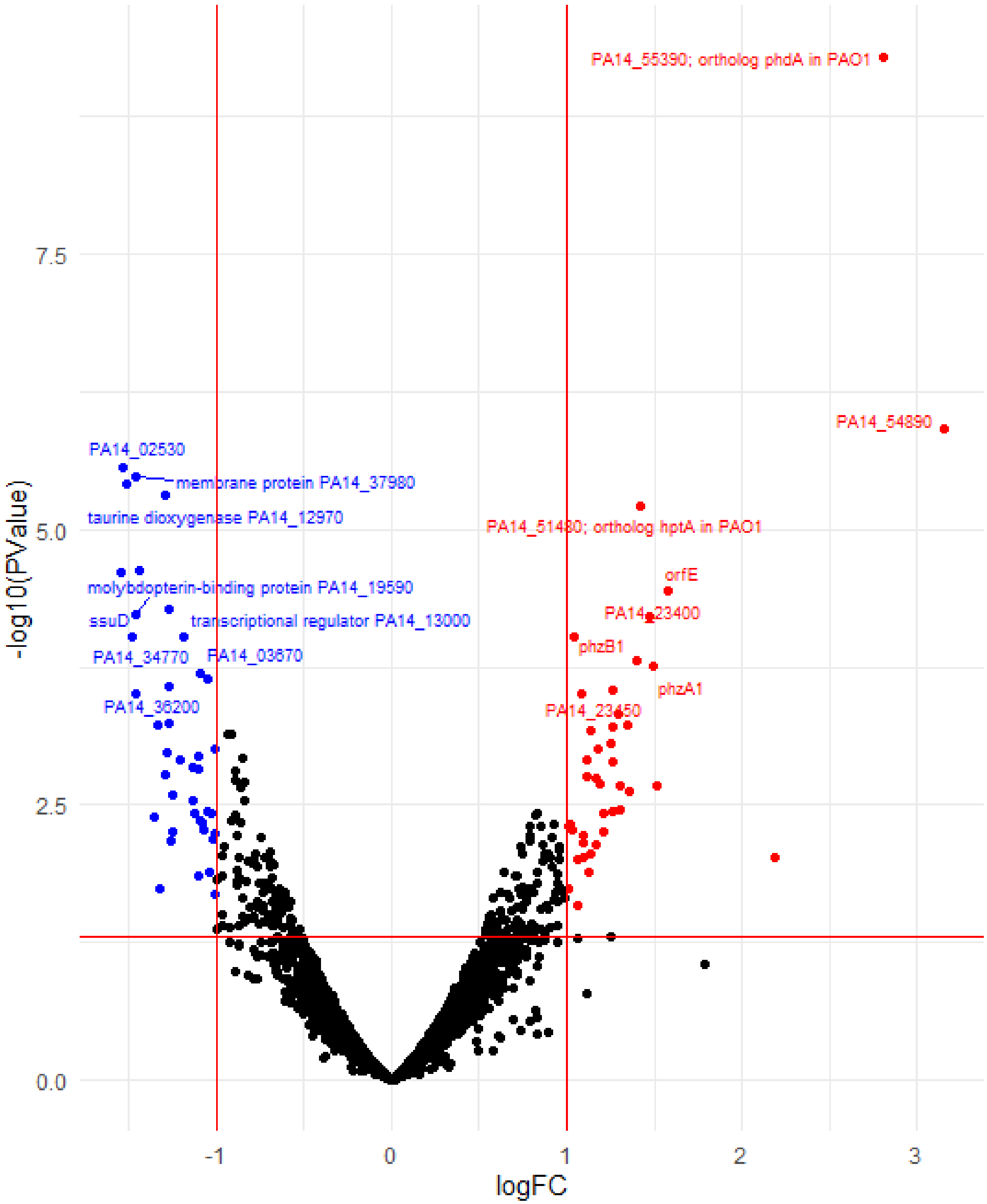

Figure S25: Volcano plot of the RNA-seq analysis for RSCV_1 compared to RSCV_3.

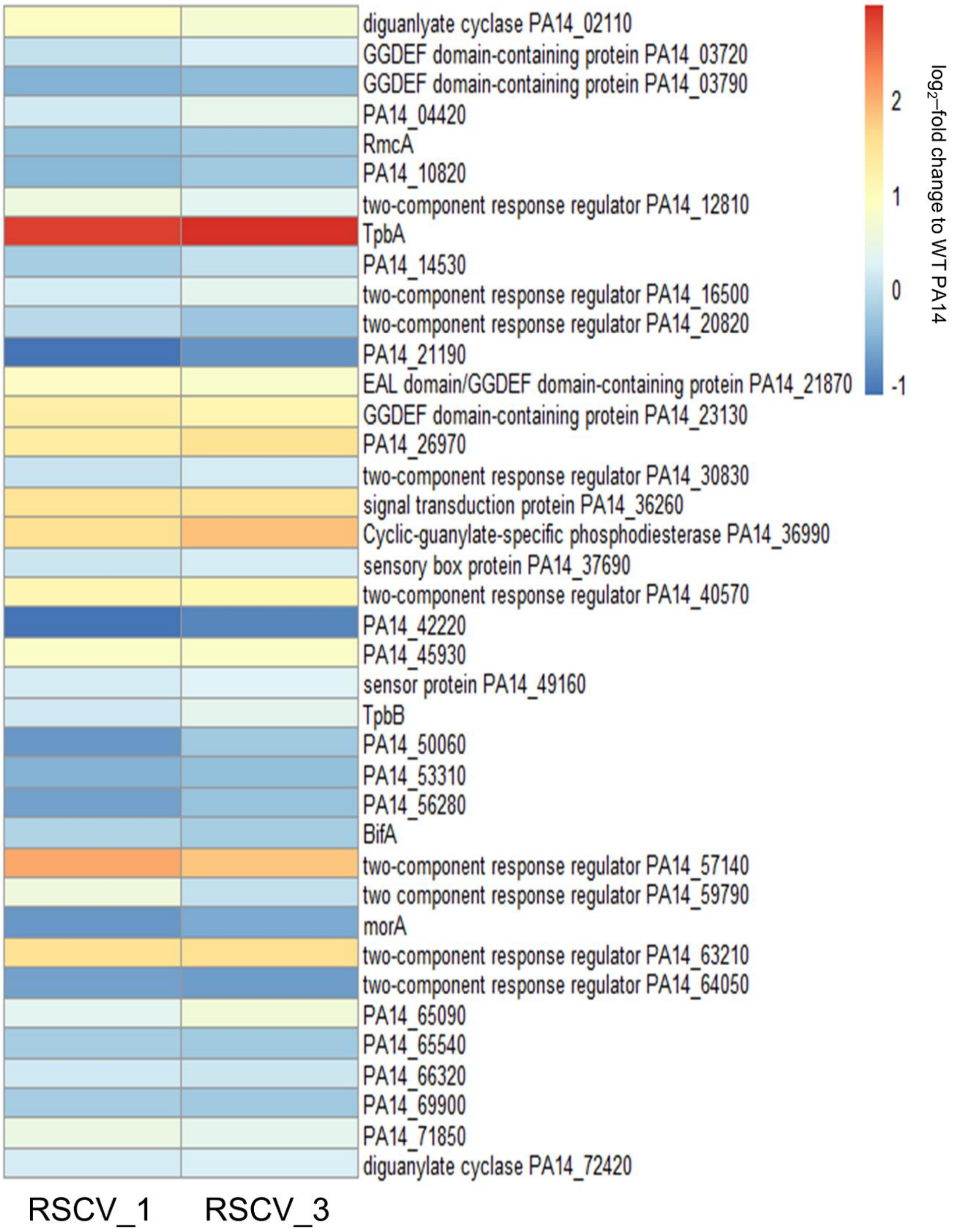

Figure S26: Heat map showing the log_2-fold-changes of genes known to be related to the signaling molecule cyclic-di-GMP in *P. aeruginosa*.

```
WT PA14/1-1416 VSTPSLTPSPPPAGDPLTVPDRPAAKAGLLLLVLLMLGLLLWQMSQELRQQERFEHERAA
RSCV_3/1-1415  VSTPSLTPSPPPAGDPLTVPDRPAAKAGLLLLVLLMLGLLLWQMSQELRQQERFEHERAA

WT PA14/1-1416 AQLDRLNDRLSLTLELKARTALALLPGMPPSERGEIQGRLLPRISDALPQVRQLQWVDSA
RSCV_3/1-1415  AQLDRLNDRLSLTLELKARTALALLPGMPPSERGEIQGRLLPRISDALPQVRQLQWVDSA

WT PA14/1-1416 PQTGSPSPETTLPEQLRQHAGSGLYHYCLDPRDGESLYLTLREPGSRRDSGFWLLHLASD
RSCV_3/1-1415  PQTGSPSPETTLPEQLRQHAGSGLYHYCLDPRDGESLYLTLREPGSRRDSGFWLLHLASD

WT PA14/1-1416 SLAQWSPDLPTGNLLWRLEDQYAGRVLWHTPGSTALLDDMQTLGVEPLRNSDWQLRGLYD
RSCV_3/1-1415  SLAQWSPDLPTGNLLWRLEDQYAGRVLWHTPGSTALLDDMQTLGVEPLRNSDWQLRGLYD

WT PA14/1-1416 STRVRLGLLPGIGGELAIFLLLVGVTVYMLLRLHREQQGLRAMTLASQRSLRQAATALAA
RSCV_3/1-1415  STRVRLGLLPGIGGELAIFLLLVGVTVYMLLRLHREQQGLRAMTLASQRSLRQAATALAA

WT PA14/1-1416 IDERVLVTRADGRLSYLNPQAERLFGISSAQARQHHLLGLLPDLEPGWLTDAGGDGETRS
RSCV_3/1-1415  IDERVLVTRADGRLSYLNPQAERLFGISSAQARQHHLLGLLPDLEPGWLTDAGGDGETRS

WT PA14/1-1416 ELLPLRVRGEPRTFTLSRHPLASYPALADQAWTLREQILQGGQVWVLRDVTEEQHALGVL
RSCV_3/1-1415  ELLPLRVRGEPRTFTLSRHPLASYPALADQAWTLREQILQGGQVWVLRDVTEEQHALGVL

WT PA14/1-1416 EETRRRYQDIFEGVGVALCVLDLSALRQALVEQGLDSCAALRAWLAGEPGRQQALLERIR
RSCV_3/1-1415  EETRRRYQDIFEGVGVALCVLDLSALRQALVEQGLDSCAALRAWLAGEPGRQQALLERIR

WT PA14/1-1416 ITEINDVSRRLLQIESTEQAWQRLLDHGPLQPDSLRLQVIEALIERRPVLELEATLRCGK
RSCV_3/1-1415  ITEINDVSRRLLQIESTEQAWQRLLDHGPLQPDSLRLQVIEALIERRPVLELEATLRCGK

WT PA14/1-1416 STPRHLWLQLRLPENPGEWHAVTLSLSDVTSRKEVELSLIEREKFWSDAVQAVPDTLYIH
RSCV_3/1-1415  STPRHLWLQLRLPENPGEWHAVTLSLSDVTSRKEVELSLIEREKFWSDAVQAVPDTLYIH

WT PA14/1-1416 DLHARRVIFSNHHLGQQLGYNQEELARMGENFWETLLHPDDQEYYWRIRNLQHVVGDGLL
RSCV_3/1-1415  DLHARRVIFSNHHLGQQLGYNQEELARMGENFWETLLHPDDQEYYWRIRNLQHVVGDGLL

WT PA14/1-1416 LDSQLRWRHRDGRWHWFDIREQAFSRDRSGRVARLIGVAKDITFTVEANNALRENGRRYR
RSCV_3/1-1415  LDSQLRWRHRDGRWHWFDIREQAFSRDRSGRVARLIGVAKDITFTVEANNALRENGRRYR

WT PA14/1-1416 MLAENISDVIFSTDAELNASYVSPSVQHVFGYSPEWALLNGLHQTATNPRQLGRLNGLLR
RSCV_3/1-1415  MLAENISDVIFSTDAELNASYVSPSVQHVFGYSPEWALLNGLHQTATNPRQLGRLNGLLR
WT PA14/1-1416 RVR-H-A-I-G-D-R-Q-K-L-A-E-L-R-E-N-P-G-Q-H-L-F-A-L-D-C-L-R-A-
RSCV_3/1-1415  RV-G-T-P-S-A-T-A-R-S-S-P-S-C-G-K-T-L-A-S-I-C-S-P-S-T-A-C-A-P

WT PA14/1-1416 D-G-R-K-I-P-I-E-L-R-I-V-L-M-W-D-E-H-D-R-F-E-G-L-L-G-I-A-R-D-
RSCV_3/1-1415  -T-D-A-R-F-P-S-N-C-G-S-S-*-C-G-T-S-T-T-A-S-K-A-C-S-A-S-P-A-T

WT PA14/1-1416 I-S-Q-Q-R-R-A-E-R-E-L-R-M-A-A-T-V-F-E-H-S-T-A-A-I-M-V-T-D-P-
RSCV_3/1-1415  -S-A-S-S-A-A-P-N-A-S-C-G-W-R-R-R-Y-S-N-T-P-R-R-R-S-W-S-P-T-R

WT PA14/1-1416 A-G-Y-I-V-Q-V-N-D-S-F-S-R-L-T-G-Y-S-P-A-D-V-L-D-Q-Q-P-R-L-L-
RSCV_3/1-1415  -P-A-T-S-S-R-S-T-I-P-S-R-A-*-P-A-T-A-R-P-T-C-S-T-S-S-R-A-C-S

WT PA14/1-1416 T-A-D-R-Q-E-A-N-Q-L-K-H-V-L-A-S-L-Q-H-S-G-S-W-E-G-E-I-L-Q-K-
RSCV_3/1-1415  -P-P-T-A-R-K-P-T-S-S-S-T-C-S-P-A-C-S-T-A-A-A-G-K-A-R-S-C-R-S

WT PA14/1-1416 R-K-T-G-E-L-Y-P-S-W-V-G-I-T-A-V-R-D-E-E-G-D-L-V-S-F-V-C-F-F-
RSCV_3/1-1415  -A-R-P-A-N-S-I-R-P-G-S-A-S-P-R-C-A-T-R-K-A-T-W-S-A-S-S-A-S-S

WT PA14/1-1416 S-D-I-S-E-R-K-A-S-E-R-R-I-H-R-L-A-Y-Y-D-A-L-T-H-L-P-N-R-T-L-
RSCV_3/1-1415  -A-T-S-A-S-A-R-P-A-S-G-A-S-T-A-W-P-T-T-T-P-S-P-T-C-P-T-A-R-C

WT PA14/1-1416 F-Q-D-R-L-H-T-A-L-Q-Q-A-E-R-N-G-Q-W-V-V-L-M-F-L-D-L-D-R-F-K-
RSCV_3/1-1415  -S-R-T-V-C-I-P-P-C-S-R-P-N-A-T-A-S-G-W-C-*-C-S-S-T-S-T-A-S-S

WT PA14/1-1416 P-I-N-D-S-L-G-H-A-A-G-D-R-M-L-Q-E-V-A-T-R-L-S-A-C-V-S-Q-D-D-
RSCV_3/1-1415  -R-S-T-T-P-S-A-T-P-P-A-T-A-C-C-R-K-W-R-P-A-*-A-P-A-S-A-R-T-I

WT PA14/1-1416 T-V-A-R-M-G-G-D-E-F-T-L-L-L-P-S-Q-G-D-R-E-I-A-L-K-R-A-I-Q-V-
RSCV_3/1-1415  -P-W-R-A-W-V-A-T-N-S-P-C-C-C-L-P-R-A-T-A-R-S-P-*-S-G-R-S-R-S
```

Figure S27: Amino acid alignment for the protein MorA (PA14_60870) in WT and RSCV_3. The EAL and GGDEF domains are highlighted in cyan and green respectively. The mutated stop codon in the amino acid sequence of RSCV_3 is highlighted in orange.

Table S1: Observed values (average ± standard deviation, n = number of biological replicates) for the colony diameter, growth curve slope, hydrodynamic radius, and zeta potential data.

Figure	RSCV_1	RSCV_2	RSCV_3	WT PA14	WT BF	WT ECM	WT SP
Figure 1A: Colony Diameters	537.8 ± 46.7 µm, n = 33	479.2 ± 46.8 µm, n = 34	628.7 ± 27.2 µm, n = 21	4837.3 ± 674.0 µm, n = 9	3517.3 ± 692.4 µm, n = 40	1584.0 ± 439.8 µm, n = 77	2870.1 ± 645.2 µm, n = 73
Figure 1B: Growth Curve Slopes	0.0549 ± 0.0024, n = 3	0.0451 ± 0.0058, n = 3	0.0545 ± 0.0024, n = 3	0.0943 ± 0.0022, n = 3	0.1002 ± 0.0027, n = 3	0.0910 ± 0.0053, n = 4	0.0870 ± 0.0034, n = 3
Figure 1C: Zeta Potentials	-18.91 ± 0.43 mV, n = 4	-18.57 ± 0.41 mV, n = 4	-17.81 ± 0.76 mV, n = 4	-17.22 ± 0.84 mV, n = 22	-18.10 ± 0.63 mV, n = 11	-15.70 ± 0.48 mV, n = 11	-23.42 ± 2.93 mV, n = 12
Figure 1D: Hydrodynamic radii	377.83 ± 22.71 nm, n = 4	428.78 ± 47.89 nm, n = 4	439.29 ± 15.67 nm, n = 4	256.78 ± 44.48 nm, n = 12	502.90 ± 43.14 nm, n = 11	471.14 ± 52.05 nm, n = 11	127.52 ± 13.65 nm, n = 9

Table S2: Observed values (average ± standard deviation, n = number of biological replicates) for the cyclic-di-GMP and pGpG quantification data in picomoles per 10^9 CFUs.

Figure	RSCV_1	RSCV_2	RSCV_3	WT PA14	Mid-log WT
Figure 2A: Cyclic-di-GMP Levels	16.08 ± 9.12, n = 9	16.38 ± 7.80, n = 9	8.53 ± 2.95, n = 10	5.88 ± 1.97, n = 9	3.12 ± 2.29, n = 4
Figure 2B: pGpG Levels	12.31 ± 8.16, n = 6	6.11 ± 3.52, n = 7	5.16 ± 1.86, n = 7	4.79 ± 0.99, n = 6	N/A

Table S3: Observed values (average ± standard deviation, n = number of biological replicates) for the Congo red and crystal violet data.

Figure	RSCV_1	RSCV_2	RSCV_3	WT PA14
Figure 3A: Congo Red Measurements	1.21 ± 0.0777 A.U., n = 5	1.03 ± 0.0762 A.U., n = 5	1.35 ± 0.118 A.U., n = 5	1.54 ± 0.188 A.U., n = 22
Figure 3B: Crystal Violet Biofilm Assay	0.605 ± 0.238 A.U., n = 18	1.48 ± 0.381 A.U., n = 18	0.669 ± 0.352 A.U., n = 18	0.114 ± 0.019 A.U., n = 18

Table S4: Observed values (average ± standard deviation, n = number of biological replicates) for swarming motility, twitching motility, and biofilm initiation.

Figure	RSCV_1	RSCV_2	RSCV_3	WT PA14	Tn-pilT
Figure 4A: Swarming Motility	56.3 ± 13.5 mm^2 , n = 3	27.4 ± 5.9 mm^2, n = 3	16.6 ± 2.8 mm^2, n = 3	2638.4 ± 729.2 mm^2 , n = 4	N/A
Figure 4B: Twitching Motility	2.39 ± 0.629 mm^2, n = 5	1.25 ± 0.672 mm^2, n = 4	3.74 ± 1.39 mm^2, n = 4	38.1 ± 12.7 mm^2, n = 5	15.3 ± 2.69 mm^2, n = 3
Figure 4C: Biofilm Initation	0.335 ± 0.0483 A.U., n = 5	0.360 ± 0.0811 A.U., n = 6	0.428 ± 0.0417 A.U., n = 5	0.212 ± 0.0344 A.U., n = 6	N/A

Table S5: Observed values (average ± standard deviation, n = number of biological replicates) for the phenazine quantification data.

Sample	Pyocyanin	Phenazine-1-carboxylic acid	Phenazine-1-carboxamide	Hydroxyphenazine
WT PA14	34.69 ± 3.46 µM, n = 5	6.87 ± 1.95 µM, n = 6	4.49 ± 1.24 µM, n = 6	7.14 ± 1.38 µM, n = 5
RSCV_1	21.83 ± 1.53 µM, n = 5	7.65 ± 0.66 µM, n = 5	5.28 ± 1.04 µM, n = 6	15.41 ± 1.91 µM, n = 5
RSCV_2	21.31 ± 1.79 µM, n = 5	7.20 ± 1.12 µM, n = 5	4.87 ± 0.40 µM, n = 5	13.12 ± 2.41 µM, n = 5
RSCV_3	22.15 ± 1.66 µM, n = 5	4.65 ± 1.52 µM, n = 6	3.91 ± 0.92 µM, n = 5	11.50 ± 0.79 µM, n = 5

**Table S6:** Observed values (average ± standard deviation, n = number of biological replicates) for the pyochelin and pyoverdine quantification data.				
**Figure**	**RSCV_1**	**RSCV_2**	**RSCV_3**	**WT PA14**
Figure 5B: Pyochelin Quantification	54484.3 ± 3071.5 A.U., n = 4	44248.6 ± 2768.6 A.U., n = 5	49283.8 ± 4575.5 A.U., n = 4	35430.4 ± 4455.6 A.U., n = 5
Figure 5C: Pyoverdine Quantification	22855.5 ± 515.7 A.U., n = 4	26408.8 ± 2478.1 A.U., n = 6	22749.2 ± 1125.4 A.U., n = 5	4922.6 ± 115.1 A.U., n = 5

Table S7: Table of gene mutations shared between RSCV_1 and RSCV_2.

Locus Tag	SNP Type (vs. WT)	Base-pair Location in RSCV_1 (vs. WT)	Base-pair Location in RSCV_2 (vs. WT)	Gene Name/Protein Function
PA14_00980	Deletion	96,482	96,484	*fha1*
PA14_08600	Deletion	737,371	737,385	23S rRNA
PA14_16480	Substitution (A to G)	1,412,866	1,412,866	*wspF*
PA14_18380	Deletion	1,579,775	1,579,276	mannose-1-phosphate guanylyltransferase
PA14_20000	Substitution (C to A)	1,721,529	1,721,558	transmembrane sensor
PA14_27180	Deletion	2,361,635	2,361,661<br>2,361,672	putative L,D-transpeptidase
PA14_35740	Deletion	3,177,433<br>3,177,947	3,179,055	transposase
PA14_41420	Deletion	3,693,691	3,693,706<br>3,693,991	Hypothetical protein
PA14_61850	Deletion	5,516,922	5,516,913	TonB-dependent receptor
PA14_67210	Deletion	5,999,179	5,999,034	type VI secretion lipase immunity protein, Tli5b1

Table S8: Table of gene mutations shared between RSCV_1 and RSCV_3.

Locus Tag	SNP Type (vs. WT)	Base-pair Location in RSCV_1 (vs. WT)	Base-pair Location in RSCV_3 (vs. WT)	Gene Name/Protein Function
PA14_16480	Substitution (A to G)	1,412,866	1,412,866	*wspF*
PA14_23450	Deletion	2,039,334	2,039,363	NAD dependent epimerase/dehydratase
PA14_51960	Deletion	4,613,353	4,613,363	ribonuclease
PA14_59830	Deletion	5,329,853	5,329,841	helicase
PA14_61850	Deletion	5,516,922	5,516,907	TonB-dependent receptor
PA14_64110	Deletion	5,713,828	5,713,813	acetyl-CoA carboxylase biotin carboxylase subunit
PA14_65540	Deletion	5,837,778	5,837,765	*FimX*
PA14_72490	Deletion	6,457,345	6,458,538	DNA polymerase I
PA14_73230	Deletion	6,521,351	6,521,354	F0F1 ATP synthase subunit epsilon

Table S9: Table of gene mutations shared between RSCV_2 and RSCV_3.

Locus Tag	SNP Type (vs. WT)	Base-pair Location in RSCV_2 (vs. WT)	Base-pair Location in RSCV_3 (vs. WT)	Gene Name/Protein Function
PA14_16480	Substitution (A to G)	1,412,866	1,412,866	*wspF*
PA14_28020	Deletion	2,425,762	2,425,780	Hypothetical protein
PA14_36370	Deletion	3,239,817	3,239,692	carboxylate-amine ligase
PA14_38860	Deletion	3,465,687	3,466,961	quinoprotein alcohol dehydrogenase
PA14_48160	Deletion	4,287,538	4,287,552	sensor/response regulator hybrid
PA14_51320	Deletion	4,559,581	4,559,589	Putative beta-barrel assembly-enhancing protease
PA14_52440	Deletion	4,653,723	4,653,737	Hypothetical protein
PA14_54930	Deletion	4,873,425	4,873,442	non-ribosomal peptide synthetase
PA14_61850	Deletion	5,516,913	5,516,907	TonB-dependent receptor
PA14_62580	Deletion	5,583,701	5,583,694<br>5,584,114	3-methyl-2-oxobutanoate hydroxymethyltransferase
PA14_62760	Deletion	5,600,269	5,600,259	translation initiation factor IF-2
PA14_70070	Deletion	6,249,354	6,248,267	Hypothetical protein
PA14_70880	Deletion	6,315,270	6,315,265	23S rRNA

Table S10: Full list of detected mutations in motility related genes in RSCV_1, RSCV_2, or RSCV_3.

Locus Tag	SNP Type	Sample	Base-pair Location	Gene Name/Protein Function
PA14_60310	Deletion	RSCV_1	5,373,709	type 4 fimbrial biogenesis protein *PilY1*
PA14_60320	Deletion	RSCV_1	5,375,954	type 4 fimbrial biogenesis protein *PilE*
PA14_37000	Deletion	RSCV_2	3,295,550	chaperone *CupA5*
PA14_37010	Deletion	RSCV_2	3,296,206	fimbrial subunit *CupA4*
PA14_37030	Deletion	RSCV_2	3,297,441	*cupA3*
PA14_60290	Deletion	RSCV_2	5,371,989	type 4 fimbrial biogenesis protein *PilW*
PA14_67560	Deletion	RSCV_2	6,036,023	regulation of biofilm formation and flagellum cell motility *typA*
PA14_59250	Deletion	RSCV_3	5,278,506	*pilN2*
PA14_59270	Deletion	RSCV_3	5,280,695	type IV b pilus protein

Table S11: Selected locus tag conversions for locus tags discussed in this work

Old Tags	New Tags
PA14_02110	PA14_RS00865
PA14_02200	PA14_RS00895
PA14_02530	PA14_RS01020
PA14_02610	PA14_RS01050
PA14_03670	PA14_RS01505
PA14_03720	PA14_RS01525
PA14_03790	PA14_RS01550
PA14_04420	PA14_RS01800
PA14_05180	PA14_RS02100
PA14_08370	PA14_RS03365
PA14_08600	PA14_RS03465
PA14_09470	PA14_RS03840
PA14_09480	PA14_RS03845
PA14_10820	PA14_RS04360
PA14_11810	PA14_RS04780
PA14_12810	PA14_RS05160
PA14_12940	PA14_RS05210
PA14_12960	PA14_RS05215
PA14_12970	PA14_RS05220
PA14_13000	PA14_RS05235
PA14_14530	PA14_RS05815
PA14_16430	PA14_RS06585
PA14_16480	PA14_RS06610
PA14_16500	PA14_RS06615
PA14_17880	PA14_RS07180
PA14_18380	PA14_RS07380
PA14_19570	PA14_RS07830
PA14_19590	PA14_RS07840
PA14_20000	PA14_RS08005
PA14_20820	PA14_RS08370
PA14_21120	PA14_RS08495
PA14_21190	PA14_RS08530
PA14_21870	PA14_RS08830
PA14_23130	PA14_RS09370
PA14_23370	PA14_RS09465
PA14_23380	PA14_RS09470
PA14_23390	PA14_RS09475
PA14_23400	PA14_RS09480
PA14_23450	PA14_RS29920
PA14_24350	PA14_RS09860
PA14_26970	PA14_RS10910
PA14_27180	PA14_RS10990
PA14_27630	PA14_RS11185
PA14_27640	PA14_RS11190
PA14_28020	PA14_RS11365

PA14_30830	PA14_RS12580
PA14_32985	PA14_RS13450
PA14_34770	PA14_RS14150
PA14_34780	PA14_RS14155
PA14_35740	PA14_RS14530
PA14_36200	PA14_RS14725
PA14_36260	PA14_RS14745
PA14_36370	PA14_RS14800
PA14_36990	PA14_RS15060
PA14_37000	PA14_RS15065
PA14_37010	PA14_RS15070
PA14_37030	PA14_RS15075
PA14_37690	PA14_RS15335
PA14_37980	PA14_RS15440
PA14_38395	PA14_RS15605
PA14_38860	PA14_RS15790
PA14_39780	PA14_RS16115
PA14_40570	PA14_RS16450
PA14_41420	PA14_RS16795
PA14_42220	PA14_RS17130
PA14_45930	PA14_RS18640
PA14_47860	PA14_RS19450
PA14_48160	PA14_RS19565
PA14_48240	PA14_RS19590
PA14_49160	PA14_RS19965
PA14_50060	PA14_RS20335
PA14_50240	PA14_RS20390
PA14_50480	PA14_RS20485
PA14_51320	PA14_RS20865
PA14_51340	PA14_RS20875
PA14_51410	PA14_RS20900
PA14_51480	PA14_RS20935
PA14_51960	PA14_RS21155
PA14_52130	PA14_RS21225
PA14_52440	PA14_RS21350
PA14_53310	PA14_RS21705
PA14_54890	PA14_RS22355
PA14_54930	PA14_RS22375
PA14_55940	PA14_RS22830
PA14_56070	PA14_RS22880
PA14_56280	PA14_RS22965
PA14_56390	PA14_RS22995
PA14_57140	PA14_RS23295
PA14_59250	PA14_RS24185
PA14_59270	PA14_RS24190
PA14_59790	PA14_RS24390
PA14_59830	PA14_RS24405
PA14_60290	PA14_RS24615
PA14_60310	PA14_RS24625
PA14_60320	PA14_RS24635
PA14_60870	PA14_RS24870
PA14_61500	PA14_RS25125

PA14_61850	PA14_RS25270
PA14_62200	PA14_RS25425
PA14_62580	PA14_RS25575
PA14_62760	PA14_RS25650
PA14_63210	PA14_RS25850
PA14_64050	PA14_RS26180
PA14_64110	PA14_RS26205
PA14_64530	PA14_RS26375
PA14_65090	PA14_RS26615
PA14_65540	PA14_RS26775
PA14_66320	PA14_RS27070
PA14_67210	PA14_RS31055
PA14_67560	PA14_RS27565
PA14_69900	PA14_RS28490
PA14_70070	PA14_RS28550
PA14_70880	PA14_RS28865
PA14_71850	PA14_RS29255
PA14_72420	PA14_RS29480
PA14_72490	PA14_RS29510
PA14_73230	PA14_RS29810

Supplemental File 1: RNA-seq normalized raw count file for RSCV_1, RSCV_3, and stationary WT PA14.

Supplemental File 2: File containing all of the differentially expressed genes between WT PA14 compared to both RSCV_1 and RSCV_3.

Supplemental File 3: File containing the full gene analysis for the stationary WT PA14 versus RSCV_1 and RSCV_3 RNA-seq comparison.

Supplemental File 4: File containing all of the differentially expressed genes between RSCV_1 and RSCV_3.

Supplemental File 5: File containing the full gene analysis for the RSCV_1 versus RSCV_3 RNA-seq comparison.

Supplemental File 6: File containing all of the differentially expressed genes between WT PA14 and RSCV_1.

Supplemental File 7: File containing all of the differentially expressed genes between WT PA14 and RSCV_3.

Supplemental File 8: File containing the full gene analysis for the stationary WT PA14 versus RSCV_1 RNA-seq comparison.

Supplemental File 9: File containing the full gene analysis for the stationary WT PA14 versus RSCV_3 RNA-seq comparison.
